# Poly(ADP-ribose) glycohydrolase enforces p21 degradation via dePARylation to promote gastric cancer progression

**DOI:** 10.1172/JCI195538

**Published:** 2026-01-15

**Authors:** Yangchan Hu, Qimei Bao, Yixing Huang, Yan Wang, Xin Zhao, Junjun Nan, Yuxin Meng, Mingcong Deng, Yuancong Li, Zirui Zhuang, Hanyi He, Dan Zu, Yuke Zhong, Chunkai Zhang, Bing Wang, Ran Li, Yanhua He, Qihan Wang, Min Liu, John A. Tainer, Yin Shi, Xiangdong Cheng, Ji Jing, Zu Ye

**Affiliations:** 1 Zhejiang Cancer Hospital, Hangzhou Institute of Medicine (HIM), Chinese Academy of Sciences, Hangzhou, Zhejiang, China.; 2 College of Pharmaceutical Science, Zhejiang University of Technology, Hangzhou, China.; 3 Department of Biochemistry and Department of Pulmonology, Children’s Hospital, Zhejiang University School of Medicine, National Clinical Research Center for Children and Adolescents’ Health and Diseases, Hangzhou, China.; 4 Collaborative Innovation Center of Yangtza River Delta Region Green Pharmaceuticals, Zhejiang University of Technology, Hangzhou, China.; 5 School of Molecular Medicine, Hangzhou Institute for Advanced Study, University of Chinese Academy of Sciences (UCAS), Hangzhou, China.; 6 School of Life Sciences, Tianjin University, Tianjin, China.; 7 College of Biological Sciences, University of California, Davis, California, USA.; 8 Departments of Molecular and Cellular Oncology and Cancer Biology, The University of Texas MD Anderson Cancer Center, Houston, Texas, USA.; 9 Key Laboratory of Prevention, Diagnosis and Therapy of Gastrointestinal Cancer of Zhejiang Province, Hangzhou, China.; 10 Zhejiang Provincial Research Center for Upper Gastrointestinal Tract Cancer, Zhejiang Cancer Hospital, Hangzhou, China.; 11 Guangxi Key Laboratory of Early Prevention and Treatment for Regional High Frequency Tumor, Nanning, China.; 12 Key Laboratory of Early Prevention and Treatment for Regional High Frequency Tumor (Guangxi Medical University), Ministry of Education, Nanning, China.

**Keywords:** Cell biology, Gastroenterology, Oncology, Drug screens, Gastric cancer, Ubiquitin-proteosome system

## Abstract

Dysregulation of cell cycle checkpoints is a cancer hallmark, with ubiquitination-controlled protein stability playing a pivotal role. Although p21, a key cyclin-dependent kinase inhibitor, is tightly regulated by ubiquitin-mediated degradation, the key upstream modulators of its ubiquitination remain incompletely defined. Here, we identify poly(ADP-ribose) glycohydrolase (PARG) as a regulator of p21 stability in gastric cancer (GC) cells. We show that PARG expression is markedly upregulated in GC tissues and correlates with poor patient prognosis. Functional assays revealed that genetic depletion of PARG triggers G2/M phase arrest and impairs GC cell proliferation. Mechanistically, we demonstrate that PARG loss enhances p21 PARylation, which disrupts its association with E3 ubiquitin ligase, thereby reducing K48-linked ubiquitination and leading to p21 protein stabilization. Moreover, we identify lysine residues K161 and K163 as critical sites for PARG-mediated regulation of p21 ubiquitination. Our findings reveal a posttranslational regulatory axis in which PARG governs cell cycle progression by modulating the PARylation-dependent ubiquitination of p21. These results broaden the understanding of p21 regulation in cancer and highlight PARG as a potential therapeutic target for GC treatment.

## Abstract 2

PARG enforces p21 proteasomal degradation via dePARylation to promote gastric cancer progression. Targeting PARG offers a new therapeutic strategy for gastric cancer.

## Introduction

Gastric cancer (GC), a highly malignant neoplasm of the gastrointestinal tract, represents a substantial global health burden, ranking fifth in both global incidence and mortality among all malignancies in 2022 (1). This disease is characterized by its aggressive biological behavior and remarkable molecular heterogeneity (2). Due to nonspecific early clinical manifestations, limited screening implementation, and insufficient reliable biomarkers, the majority of patients present with advanced-stage disease at diagnosis, resulting in unfavorable clinical outcomes (3–5). Currently, surgical resection remains the cornerstone of curative treatment for early-stage GC, offering the only potential for complete remission (6). For terminal patients, comprehensive treatment strategies incorporating surgery, radiotherapy, chemotherapy, immunotherapy, and targeted therapies are implemented based on pathological characteristics and clinical staging (7). However, the limited availability of therapeutic targets and the frequent development of drug resistance substantially compromise treatment efficacy in GC management (8, 9). Therefore, identification of novel therapeutic targets, in-depth exploration of molecular pathogenesis, and development of innovative treatment modalities are crucial directions in GC research and clinical practice.

Genomic instability, a hallmark of gastric tumorigenesis, is closely associated with dysregulation of the DNA damage response (DDR) (10–12). Among key DDR mediators, poly(ADP-ribose) polymerase (PARP) catalyzes the formation of poly(ADP-ribose) (PAR) chains at sites of DNA damage, orchestrating repair processes through PARylation signaling (13). While PARP inhibitors have been explored in cancer therapy, their clinical efficacy in GC remains limited, highlighting the complexity of PARylation dynamics beyond classical DNA repair functions (14). In this context, increasing attention has been directed toward enzymes involved in PAR turnover, such as poly(ADP-ribose) glycohydrolase (PARG), to better understand their biological roles in cancer.

PARG functions as the primary enzyme responsible for degrading PAR chains by hydrolyzing ribose-ribose bonds, thereby regulating the reversibility of PARylation modifications (15). Through modulating PAR levels, PARG plays a critical role not only in the DDR pathway but also in maintaining broader cellular homeostasis (16, 17). Consequently, PARG structures have been determined and preclinical inhibitors have been developed that kill cancer cells with potential for targeting GC (15, 18, 19). Recent studies have implicated PARG in multiple malignancies, including breast (20), ovarian (21, 22), hepatocellular (23), and colorectal carcinomas (24). Beyond cancer, PARG has been linked to immune regulation (24), ischemic injury (25, 26), neurological disorders (27), and inflammatory diseases (28). Nevertheless, the functional relevance and regulatory mechanisms of PARG in GC remain largely unexplored.

Evidence suggests that PARG inhibition or deficiency stalls replication forks (18), disrupts cell cycle progression (22, 29), and promotes mitotic catastrophe (30, 31); however, the underlying molecular mechanisms remain to be fully elucidated. We reasoned that PARG impacts could reflect an interaction with p21 (CDKN1A), also designated as p21^WAF1/CIP1^. p21 (CDKN1A) is a cyclin-dependent kinase inhibitor (CKI) that regulates cell cycle progression by inhibiting CDK4/6-cyclin D complexes at the G1/S transition and CDK2-cyclin E complexes at the G2/M transition (32). The regulated degradation of p21 is crucial for proper cell cycle progression and cellular proliferation (33, 34). The primary p21 degradation pathways involve both the ubiquitin-proteasome system (35) and the autophagy-lysosomal pathway (36). Several E3 ubiquitin ligases, including SKP2 (37), CRL4 (Cdt2) (38), CUL4B (39), RNF126 (40), and UHRF2 (41), are regulators of p21 ubiquitination. Besides regulating the cell cycle, p21 participates in diverse cellular processes, including DNA replication and repair (42), cellular proliferation (43) and differentiation, senescence, and apoptosis, thereby playing critical roles in tumorigenesis and cancer progression (44, 45). Notably, the repression of p21 expression promoted the development of GC (46), and the upregulation of p21 expression led to the inhibition of the proliferation of GC cells (47).

Our findings show that genetic ablation of PARG induces G2/M phase arrest in GC cells, consequently suppressing cellular proliferation. Moreover, PARG deficiency significantly increased genomic instability in GC cells. At the molecular level, we established a direct protein-protein interaction between PARG and p21. PARG deficiency resulted in elevated p21 PARylation, which subsequently impaired its binding to the E3 ubiquitin ligase, ultimately reducing p21 ubiquitination and enhancing its protein stability. We further characterized the specific ubiquitination patterns and critical lysine residues involved in PARG-mediated regulation of p21 ubiquitination. Through high-throughput screening, we discovered that ginsenoside C-K (G C-K) significantly enhanced the sensitivity of PARG-deficient GC cells, potentially through p21-mediated mechanisms. Collectively, our findings demonstrate that elevated PARG expression facilitates GC cell proliferation and modulates p21 protein expression via ubiquitination-mediated regulation. These results provide insights into PARG and p21 biology and GC pathogenesis, identify PARG as a potential therapeutic target, and offer mechanistically informative research directions for clinical intervention strategies in GC treatment.

## Results

### PARG drives cell proliferation by orchestrating cell cycle progression.

**Figure 1:**
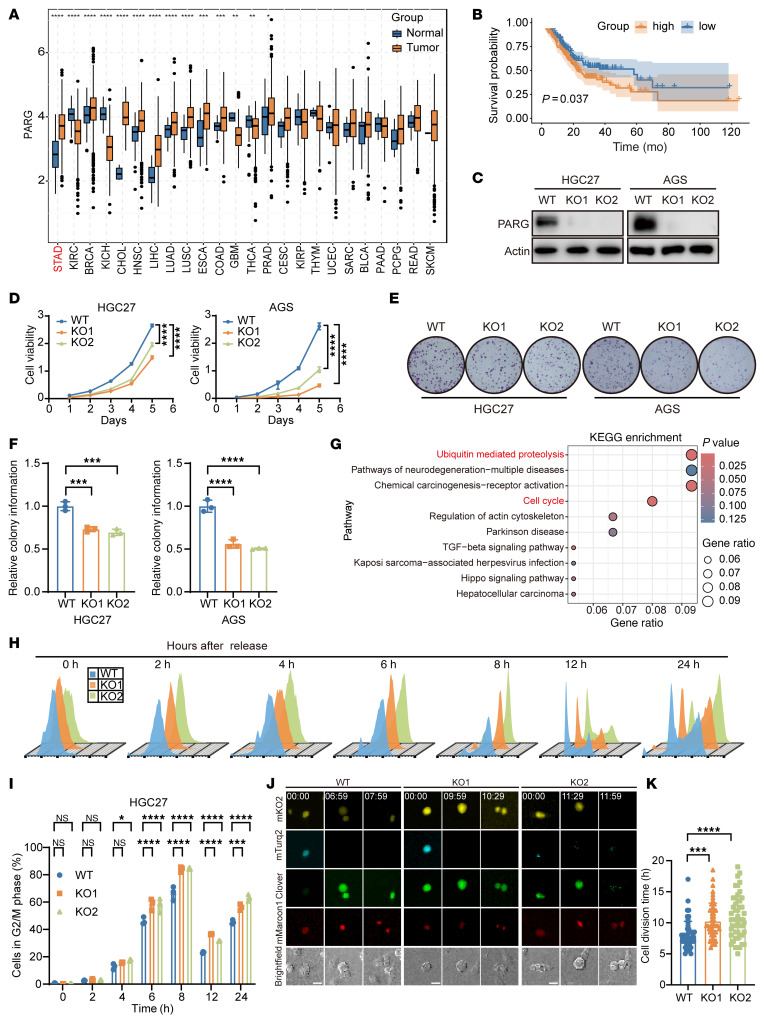
PARG promotes cell proliferation and orchestrates cell cycle progression. (**A**) TCGA database analysis of PARG expression in pancancer. (**B**) Survival analysis of patients with GC, *P* values were determined by 2-sided log-rank test. (**C**) Western blot detection of PARG protein expression in PARG KO HGC27 and AGS cells. (**D**) Proliferation profiles of WT and PARG KO HGC27 and AGS cells detected by a CCK-8 assay, *n* = 5. (**E**) Colony formation graphs of WT and PARG KO AGS, HGC27 cells detected via a colony formation assay. (**F**) Statistical graphs of colony formation by HGC27 and AGS cells, *n* = 3. (**G**) Bubble map of the transcriptomics KEGG enrichment analysis. (**H**) Cell cycle distribution of WT and PARG KO HGC27 cells detected by the thymidine double-block assay. (**I**) Statistics of HGC27 cells in G2/M phase, *n* = 3. (**J**) FUCCI4 system virus-infected WT and PARG KO AGS cells were synchronized via the thymidine double-block method, followed by live-cell imaging via the high-quality imaging analysis system; scale bar: 50 μm. (**K**) Statistical plots of the division times of WT and PARG KO AGS cells, *n* = 50. (*P ≤ 0.05, **P ≤ 0.01, ***P ≤ 0.001, ****P ≤ 0.0001, NS, not significant, **D** and **I** by 2-way ANOVA, **F** and **K** by 1-way ANOVA. Error bars represent the mean ± SD).

Analysis of The Cancer Genome Atlas (TCGA) database showed that PARG expression is significantly elevated in most tumor types compared with their normal counterparts, particularly in GC (Figure 1A). Importantly, high PARG expression was strongly correlated with an unfavorable prognosis in patients with GC (Figure 1B). Western blot analysis of 9 GC cell lines (MKN-1, MKN-45, MKN-74, NUGC3, NUGC4, HGC27, AZ521, AGS, and KATO-3) versus normal gastric mucosal cells (GES-1) showed markedly higher PARG expression in 8 of the GC cell lines, particularly in HGC27 and AGS cells (Supplemental Figure 1A; supplemental material available online with this article; https://doi.org/10.1172/JCI195538DS1). To elucidate the biological function of PARG in GC, we generated PARG knockout (KO) HGC27 and AGS cell lines via CRISPR-Cas9 genome editing (Figure 1C). The results of functional assays revealed that PARG ablation significantly impaired cellular proliferation, as evidenced by the results of CCK-8 assays (Figure 1D) and colony formation experiments (Figure 1, E and F).

To systematically investigate the cellular consequences of PARG loss, we performed a series of phenotypic and transcriptomic analyses without preselecting specific pathways. Confocal microscopy analysis revealed that PARG KO induced chromosomal bridge formation and chromosome lagging during anaphase, resulting in defective chromosome segregation (Supplemental Figure 1B). Micronuclei (MCNs) formation assays revealed a significantly increased MCN frequency in PARG-deficient GC cells (Supplemental Figure 1C). Moreover, multinucleation assays showed increased multinucleated cell formation in PARG KO cells (Supplemental Figure 1D). Cytogenetic analysis further identified elevated endogenous chromosomal aberrations, including premature sister chromatid separation (PCS), dicentric chromosomes (Dic), ring chromosomes (Ring), and chromosomal fragments (Frag) (Supplemental Figure 2A). These results suggest that PARG KO induces genomic instability in GC cells.

We subsequently conducted transcriptome-wide profiling to identify differentially expressed genes between WT and PARG KO cells. Kyoto Encyclopedia of Genes and Genomes (KEGG) analysis highlighted significant enrichment in cell cycle–related pathways (Figure 1G). This observation suggested a potential link between PARG function and cell cycle regulation. Guided by this finding, we next focused on assessing the impact of PARG on cell cycle progression.

Cell cycle analysis under serum starvation conditions revealed a G2/M phase arrest in PARG KO cells (Supplemental Figure 2, B and C). This finding was corroborated via thymidine double-block synchronization followed by cell cycle profiling (Figure 1, H and I, and Supplemental Figure 3, A and B). Furthermore, real-time cell cycle tracking via the FUCCI4 reporter system confirmed pronounced mitotic delay in PARG-deficient cells (Figure 1, J and K). Collectively, these results suggest that high PARG expression is associated with poor prognosis in patients with GC and underscore its important role in sustaining cell cycle progression and tumor growth.

### PARG interacts with p21 to promote its proteasomal degradation via dePARylation.

**Figure 2:**
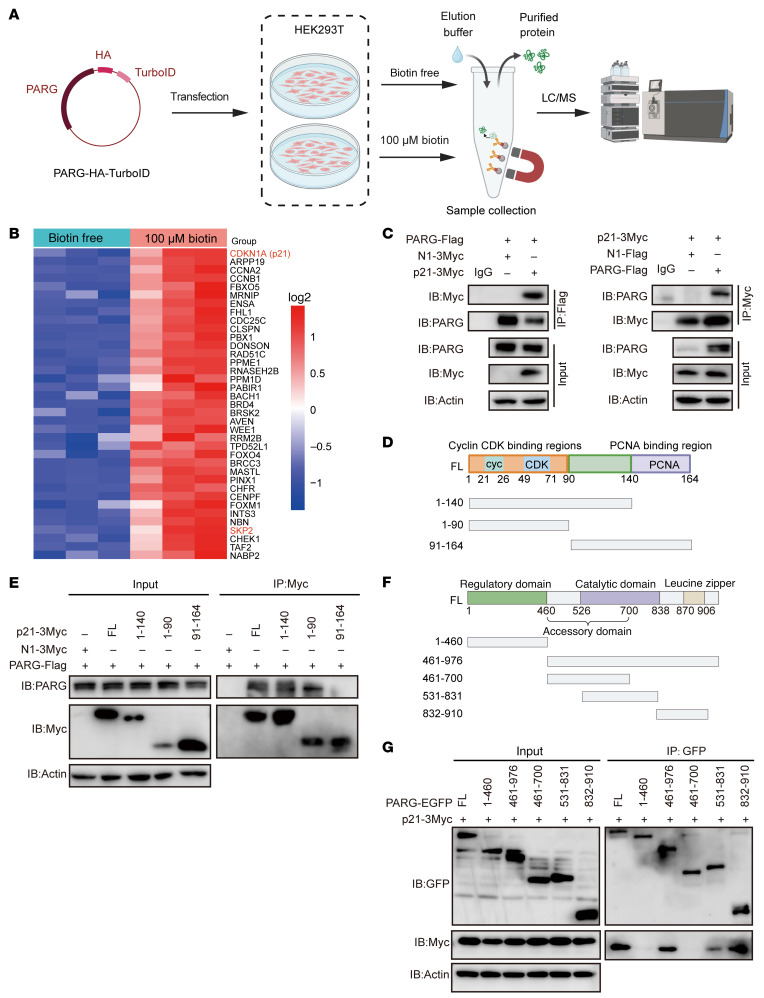
PARG interacts with p21. (**A**) Flowchart of the TurboID proximity labeling experiment. (**B**) Heatmap of the interactome of the PARG. (**C**) PARG-flag and p21-3Myc plasmids were cotransfected into HEK293T cells, and total cell lysates were immunoprecipitated with anti-flag and anti-Myc antibodies, respectively. Then, anti-Myc and anti-PARG antibodies were used to detect the immunoprecipitates, and anti-PARG and anti-Myc antibodies were used to detect the success of the experiments. (**D**) Schematic of the P21 truncation. (**E**) PARG-Flag and p21-3Myc truncated plasmids were cotransfected into HEK293T cells, and total cell lysates were immunoprecipitated with anti-Myc antibodies. Then, anti-Myc was used to detect the immunoprecipitates, and anti-PARG was used to detect the success of the experiment. (**F**) Schematic of the PARG truncation. (**G**) p21-3Myc and PARG-EGFP truncated plasmids were cotransfected into HEK293T cells, followed by coincubation with EGFP-labeled magnetic beads. Immunoprecipitates were then detected with indicated antibodies.

To elucidate the molecular mechanisms underlying PARG-mediated cell cycle regulation, we performed TurboID proximity labeling coupled with mass spectrometry analysis (Figure 2A). Subsequent GO pathway analysis of the interacting proteins revealed significant enrichment in the mitotic G2/M transition pathway, with p21 emerging as the top-ranked candidate (Figure 2B), suggesting its potential involvement in PARG-induced G2/M arrest. In addition, we also examined the cell cycle–related proteins CCNA2 and CCNB1, which are among the top candidates from the proteomic data, and found that their protein levels did not significantly change (Supplemental Figure 4A).To validate this interaction, we conducted coimmunoprecipitation (Co-IP) assays in HEK293T cells. Co-IP experiments demonstrated that p21 was consistently detected in PARG-Flag precipitates, while PARG was also present in p21-3Myc precipitates (Figure 2C), confirming their physical association. To determine whether this interaction occurs in GC cells, we repeated the experiment using AGS and HGC27 cells and confirmed a reproducible interaction between PARG and p21 (Supplemental Figure 4, B and C). Protein interaction modeling predicted PARG binding at the N-terminal domain of p21 (Supplemental Figure 4D). Subsequent Co-IP assays with p21 truncation mutants confirmed that PARG specifically interacts with amino acid residues 1–90 and 1–140 of p21 (Figure 2, D and E), definitively localizing the interaction domain to the N-terminal region. Additionally, we found that PARG binds to p21 through its C-terminal leucine zipper domain (Figure 2, F and G).

**Figure 3:**
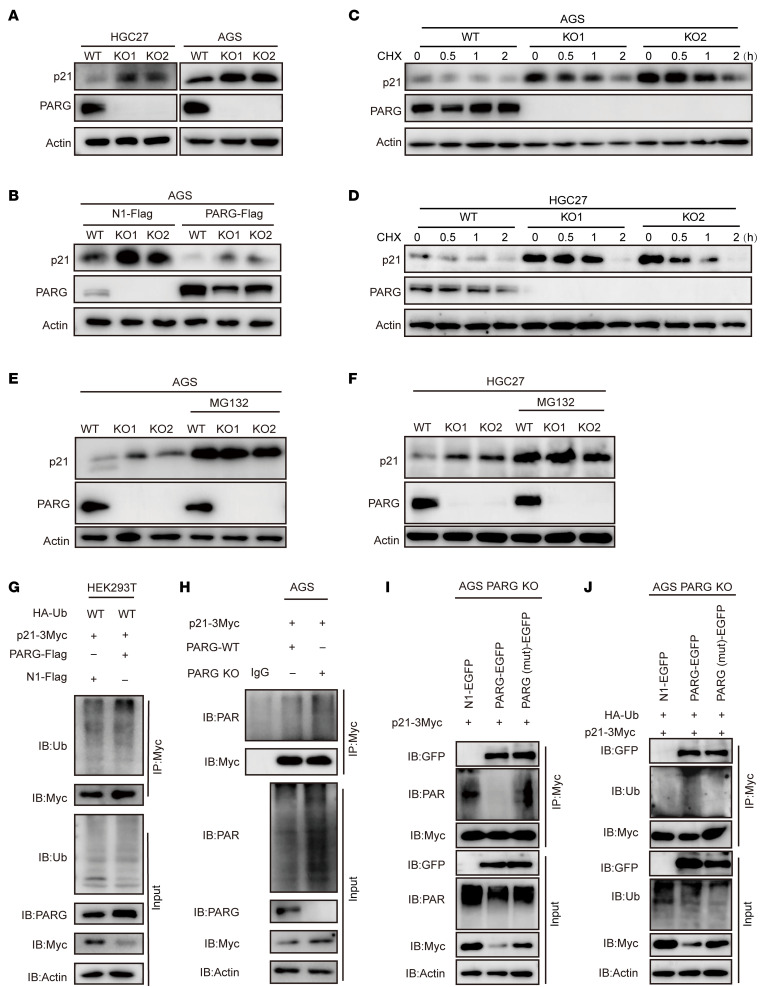
PARG drives p21 proteasomal degradation by removing its PARylation. (**A**) Western blot detection of p21 protein expression in WT and PARG KO HGC27 and AGS cells. (**B**) The PARG-Flag plasmid was transfected into WT and PARG KO AGS cells, followed by Western blot analysis of PARG-Flag transfection efficiency and its effect on p21 protein expression. (**C** and **D**) AGS and HGC27 cells were treated with 200 μg/mL CHX for 6 hours, and equal amounts of cell lysates enriched with the indicated antibodies were subjected to immunoblotting. (**E** and **F**) AGS and HGC27 cells were treated with 10 μM MG132 for 6 hours, and equal amounts of cell lysates were immunoblotted with the indicated antibodies. (**G**) p21-3Myc, PARG-Flag, and HA-Ub plasmids were transiently transfected into HEK293T cells, and the transfected HEK293T cells were treated with MG132 for 6 hours to harvest proteins. The ubiquitinated p21 protein was pulled down with an anti-Myc antibody and immunoblotted with an anti-Ub antibody. (**H**) The p21-3Myc plasmid was transfected into WT and PARG KO AGS cells, ADP-ribosylation generation was stimulated with 150 μM H_2_O_2_, and total cell lysates were immunoprecipitated with an anti-Myc antibody. Then, anti-Myc was used to detect the immunoprecipitates, and anti-PAR was used to detect the success of the experiment. (**I**) PARG KO AGS cells were cotransfected with the p21-3Myc plasmid and either the N1-EGFP plasmid or the PARG-EGFP, PARG(mut)-EGFP plasmid. ADP-ribosylation was stimulated with 150 μM H_2_O_2_, and total cell lysates were immunoprecipitated and detected with indicated antibodies. (**J**) p21-3Myc and HA-Ub plasmids were cotransfected with N1-EGFP, PARG-EGFP, or PARG(mut)-EGFP into PARG KO AGS cells. The proteins were harvested after 6 hours of MG132 treatment. The ubiquitinated p21 protein was pulled down via an anti-Myc antibody, followed by immunoblotting with anti-Ub and anti-GFP antibodies.

To further investigate the regulatory role of PARG in relation to p21, we examined p21 protein levels through Western blot experiments. The results indicated that PARG KO led to the upregulation of p21 protein expression in both the HGC27 and AGS cell lines (Figure 3A). To verify that p21 upregulation resulted directly from PARG KO, we introduced PARG-Flag overexpression plasmids into the PARG-WT, -KO1, and -KO2 cell lines and examined the effects on p21. These experiments demonstrated that PARG overexpression substantially reduced p21 protein levels, confirming that p21 expression regulation in GC cells depends on PARG (Figure 3B and Supplemental Figure 4E). We subsequently measured p21 mRNA expression levels via qPCR. The results revealed that PARG KO did not significantly affect p21 transcription levels, suggesting that PARG regulates p21 at the protein level rather than at the transcriptional level (Supplemental Figure 4F).

To further examine the specific regulatory mechanism involved, we treated PARG WT, KO1, and KO2 AGS cells with the protein synthesis inhibitor cycloheximide (CHX) and monitored p21 protein turnover. At baseline (0 h), p21 abundance was markedly higher in PARG-deficient cells than in WT cells (Figure 3, C and D), indicating enhanced p21 stability upon PARG loss. However, the very low basal level of p21 in WT cells precluded reliable determination of degradation kinetics. To overcome this limitation, cells were treated with the p21 transcription activator (APTO-253) to enhance p21 expression prior to CHX treatment. Under these optimized conditions, PARG KO significantly prolonged the half-life of the p21 protein, confirming that PARG depletion stabilizes the p21 protein (Supplemental Figure 4, G and H). The proteasome inhibitor MG132 was subsequently added to PARG KO AGS and HGC27 cells. Western blot experiments demonstrated that PARG KO resulted in the upregulation of p21 expression, and MG132 treatment led to an increase in p21 expression to the same level in all the cell lines (Figure 3, E and F). These findings suggest that PARG primarily regulates p21 through the ubiquitin-proteasome pathway. Ubiquitination assays in HEK293T cells cotransfected with HA-Ub, p21-3Myc, and PARG-Flag plasmids revealed that PARG overexpression increased p21 ubiquitination levels (Figure 3G), whereas PARG KO had the opposite effect (Supplemental Figure 5A). Given recent evidence that PARylation inhibits proteasomal degradation and promotes deubiquitination (48), we examined the p21 PARylation status. Co-IP experiments confirmed that p21 undergoes PARylation, with obviously increased PARylation levels following PARG KO (Figure 3H). In PARG KO cells, empty vectors (N1-EGFP), WT PARG (PARG-EGFP), or a catalytically inactive mutant (PARG[mut]-EGFP, where the key catalytic residues E755/756 were mutated to alanine [E755/756A]), were expressed and p21 PARylation levels and ubiquitination status were analyzed. We found that PARG-EGFP reconstitution effectively rescued the PARG KO–induced reduction in p21 PARylation and increase in p21 ubiquitination. In contrast, catalytically dead PARG(mut)-EGFP failed to do so (Figure 3, I and J). Thus, the decreased level of p21 ubiquitination in PARG KO cells may be due to a spatial blockade of p21 PARylation. Additional experiments with ubiquitin chain-specific mutants (K6R, K11R, K27R, K29R, K33R, K48R, and K63R) revealed K48-linked polyubiquitination as the predominant form involved in this regulation (Supplemental Figure 5B).To identify critical ubiquitination sites, we generated a series of Myc-tagged p21 mutants, including single-point (K16R, K75R, K141R, K154R, K161R, and K163R), multipoint (K2R [K161R/K163R], K4R [K16R/K154R/K161R/K163R]), and complete lysine (K6R) mutants. Co-IP analysis revealed that K161 and K163 are crucial sites for PARG-mediated p21 ubiquitination (Supplemental Figure 5C). Previous research mentioned that SKP2, RNF126, and Cdt2 are potential regulators of p21 ubiquitination and degradation (49). We detected SKP2, RNF126, and Cdt2 expression following PARG knockout via Western blot analysis and found no changes in their expression levels (Supplemental Figure 5, D and F). Subsequent interaction assays demonstrated that PARG overexpression enhanced p21 binding to the E3 ubiquitin ligases SKP2, RNF126, and Cdt2 (Supplemental Figure 5, G–I) and promoted p21 ubiquitination. This suggests that PARG-mediated deparylation may facilitate p21 ubiquitination by promoting E3 ubiquitin ligase recruitment.

To investigate whether PARG universally regulates p21 and the cell cycle, we knocked out PARG in several cancer models with high PARG expression, including esophageal carcinoma KYSE150 and KYSE30 cell lines, breast carcinoma MDA-MB-231 cell line, hepatocellular carcinoma Huh7 cell line, and colorectal carcinoma HCT116 cell line. The results revealed that loss of PARG induced p21 expression and cell cycle arrest at the G2/M phase in all these cell lines, suggesting that this regulation may be universal in different types of cancer (Supplemental Figure 6, A and B).

### PARG depletion triggers p21-dependent cancer suppression.

**Figure 4:**
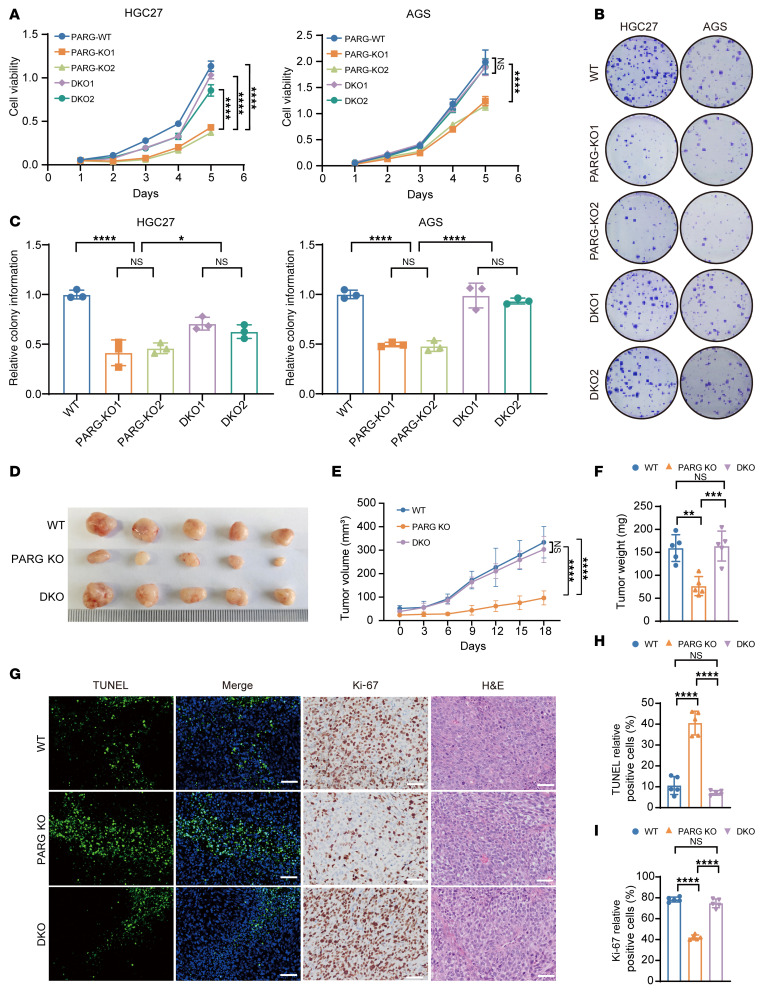
PARG depletion triggers p21-dependent cancer suppression. (**A**) Proliferation profile of the HGC27 and AGS DKO cell lines, *n* = 5. (**B**) Colony formation diagram of the HGC27 and AGS DKO cell lines. (**C**) Statistical graphs of the colony formation of the HGC27 and AGS DKO cell lines, *n* = 3. (**D**) Tumor diagram of the HGC27 xenograft model. (**E**) Tumor volume growth curve statistics of the HGC27 xenograft model, *n* = 5. (**F**) Tumor weight statistics of the HGC27 xenograft model, *n* = 5. (**G**) IHC staining of tumor tissues for TUNEL, Ki-67, and H&E staining; scale bar: 50 μm. (**H**) TUNEL-positive cell statistical graph, *n* = 5. (**I**) Statistical analysis of Ki-67–positive cells in tumor tissues, *n* = 5. (*P ≤ 0.05, **P ≤ 0.01, ***P ≤ 0.001, ****P ≤ 0.0001, NS, not significant, **A** and **E** by 2-way ANOVA, **C**, **F**, **H**, and **I** by 1-way ANOVA. Error bars represent the mean ± SD).

To determine whether the inhibition of proliferation caused by PARG KO was p21 dependent, we generated PARG and p21 double knockout (DKO) cell lines via CRISPR-Cas9 genome editing (Supplemental Figure 7A). The results revealed that the proliferation capacity of DKO1 and DKO2 cells was considerably restored compared with that of PARG KO cells (Figure 4A). Furthermore, plate cloning experiments showed that the colony formation ability of DKO1 and DKO2 cells was significantly restored compared with that of PARG KO cells (Figure 4, B and C).

To verify the regulatory role of the PARG-p21 signaling axis in the proliferative activity of GC cells at the cellular level, we investigated whether the PARG KO–induced inhibition of tumor growth was dependent on p21 via the HGC27 cell line-derived xenograft (CDX) model. The results showed that the growth rate of HGC27 cells, as well as the volume and weight of tumors, was significantly higher in mice bearing DKO tumors than in those bearing PARG KO tumors (Figure 4, D–F). Subsequent IHC staining of tumor samples from subcutaneous tumor-bearing mice revealed a substantial decrease in the percentage of TUNEL-positive cells and a significant increase in the percentage of Ki-67–positive cells after knockout of p21 expression in GC cells from PARG KO mice (Figure 4, G–I). These findings provide compelling evidence that PARG exerts its proliferative regulatory effects in GC through p21-dependent mechanisms.

### High-throughput screening identifies G C-K as synergistic lethal with PARG loss.

**Figure 5:**
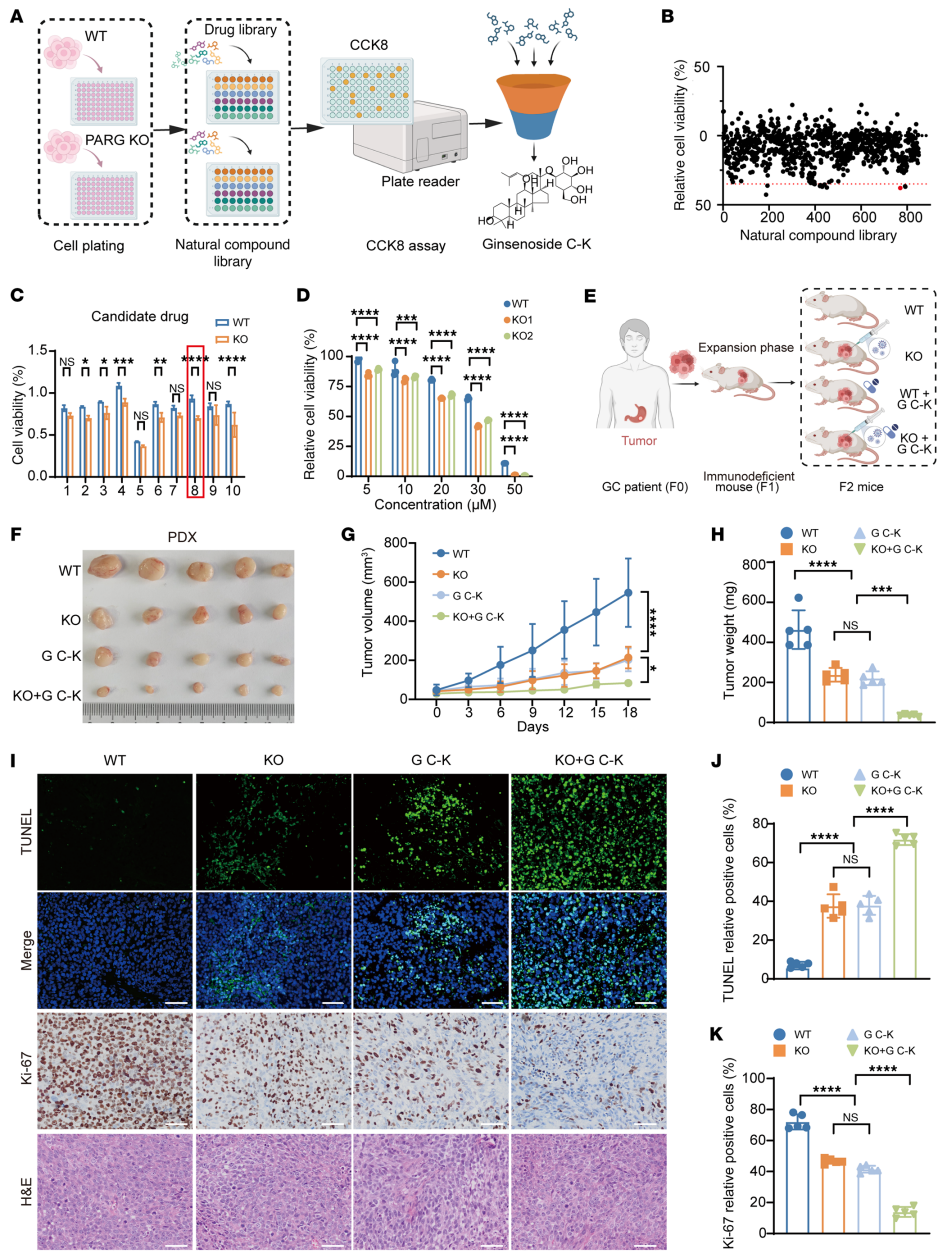
High-throughput screening identifies G C-K as synergistic lethal with PARG loss. (**A**) Flowchart of high-throughput drug screening. (**B**) Initial screening of the natural product library, *n* = 3. (**C**) Secondary screening of the top 10 drugs sensitive to PARG KO cells, *n* = 3. Compounds 1–10 represent vincristine sulfate, lappaconitine, 4-methylumbelliferone, mupirocin, diosmetin, aloin, uridine, ginsenoside C-K, saikosaponin D, and gracillin, respectively. (**D**) A CCK-8 assay was used to detect the survival rate of WT and PARG KO HGC27 cells after G C-K treatment, *n* = 3. After the cells were treated with 30 μM G C-K for 48 hours, they were incubated with CCK-8 assay reagent for 2 hours before detection. (**E**) Flowchart of PDX model construction. (**F**) Graph of G C-K-treated PDX model tumors. (**G**) Statistical graph of the tumor volume growth curve in the PDX model after G C-K treatment, *n* = 5. (**H**) Statistical graph of tumor weight in the PDX model after G C-K treatment, *n* = 5. (**I**) Graphs of IHC staining for TUNEL, Ki-67 and H&E in PDX tumor tissues; scale bar: 50 μm. (**J**) Statistical graph of TUNEL-positive cells in tumor tissues; *n* = 5. (**K**) Statistical graph of Ki-67–positive cells in tumor tissues, *n* = 5. (*P ≤ 0.05, **P ≤ 0.01, ***P ≤ 0.001, ****P ≤ 0.0001, NS, not significant, **“C**, **D**, and **G** by 2-way ANOVA, **H**, **J**, and **K** by 1-way ANOVA. Error bars represent the mean ± SD).

To enhance therapeutic efficacy while mitigating drug resistance and adverse effects, GC treatment typically employs multidrug regimens (50). The principle of drug synergy occurs when the combined effect of 2 drugs is greater than the sum of their individual effects, offering a powerful strategy to overcome compensatory pathways and enhance therapeutic outcomes (51). Further induction of damage in genomically unstable tumors promises to apply the principle of synergistic lethality to establish additional therapeutic options. Building upon our identification of PARG as a potential therapeutic target, we conducted high-throughput screening of natural product libraries to identify compounds that exhibit synergistic effects with PARG inhibition (Figure 5A). Primary screening identified 10 compounds with enhanced sensitivity in PARG KO cells (Figure 5B). Further screening revealed that G C-K, a bioactive ginseng component, had potent antitumor activity only in PARG KO cancer cells (Figure 5C). This selection was supported by the established role of p21 as a primary target of G C-K, which aligns with our previous findings regarding the PARG-p21 signaling axis in cell cycle regulation (52). Dose-response assays demonstrated concentration-dependent reductions in the viability of PARG KO1 and KO2 cells, with significantly higher sensitivity than seen in WT cells (Figure 5D and Supplemental Figure 7B). The IC_50_ value of G C-K in GES-1 cells was 51.26 μM, which was substantially higher than the IC_50_ values observed in AGS and HGC27 cells. Furthermore, at a working concentration of 30 μM, GES-1 cell viability remained at approximately 90%, indicating that 30 μM G C-K was nontoxic to normal gastric mucosa (Supplemental Figure 7, C and E). Colony formation assays confirmed that, while PARG KO alone impaired proliferative capacity, combination with 30 μM G C-K substantially inhibited growth (Supplemental Figure 7, F–I). These findings establish that PARG deficiency sensitizes GC cells to G C-K treatment. We subsequently examined the cell viability of GC cells in the presence of G C-K via a CCK-8 assay. The investigations revealed that cotreatment with the pancaspase inhibitor Z-VAD-FMK, but not the ferroptosis inhibitor Fer-1 or the pyroptosis inhibitor VX-765, significantly attenuated G C-K-induced cytotoxicity (Supplemental Figure 7J). Flow cytometric analysis confirmed significantly elevated apoptosis rates in G C-K–treated PARG KO cells relative to those in control cells (Supplemental Figure 7, K and L), demonstrating that PARG ablation potentiates G C-K–induced apoptotic cell death.

**Figure 6:**
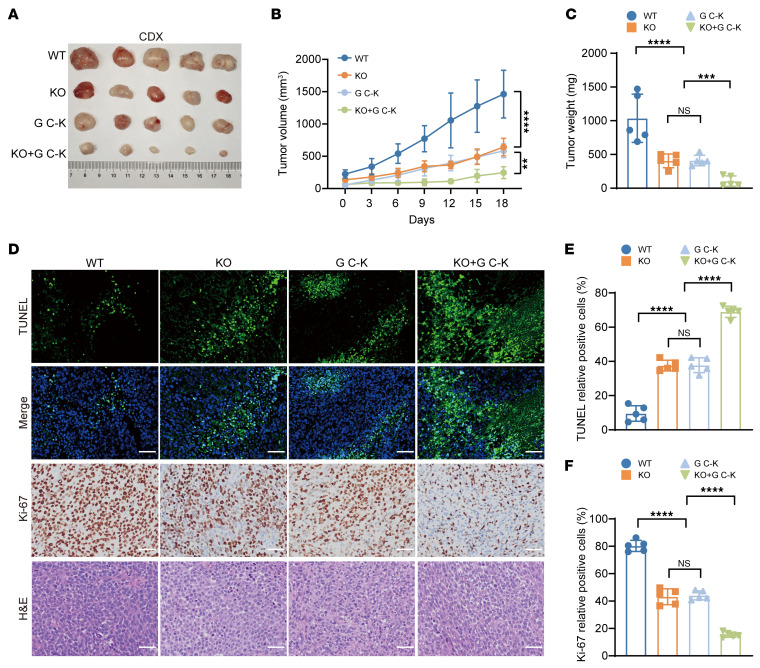
PARG deficiency enhances GC tumor sensitivity to GC-K. (**A**) Tumor plots of the HGC27 CDX model after G C-K treatment. (**B**) Tumor volume growth curve statistics of the HGC27 xenograft model after G C-K treatment, *n* = 5. (**C**) Tumor weight statistics of the HGC27 xenograft model after G C-K treatment, *n* = 5. (**D**) Graphs of IHC staining for TUNEL, Ki-67, and H&E in CDX tumor tissues; scale bar: 50 μm. (**E**) Statistical graph of TUNEL-positive cells in tumor tissues, *n* = 5. (**F**) Statistical graph of Ki-67-positive cells in tumor tissues, *n* = 5. (**P ≤ 0.01, ***P ≤ 0.001, ****P ≤ 0.0001, NS, not significant, **B** by 2-way ANOVA, **C**, **E**, and **F** by 1-way ANOVA. Error bars represent the mean ± SD).

We therefore further tested the effect of PARG KO–enhanced G C-K on the antitumor activity of GC in a patient-derived tumor xenograft (PDX) model (Figure 5E). The results demonstrated that the tumor growth rate, as well as the volume and weight of the tumors, was significantly inhibited by the PARG KO and G C-K treatments (Figure 5, F–H). Subsequent IHC staining of tumor samples from subcutaneous tumor-bearing mice revealed that the percentage of TUNEL-positive cells was significantly elevated and the percentage of Ki-67–positive cells was significantly decreased in tumors from G C-K–treated PARG KO mice (Figure 5, I–K). No significant alterations in body weight were observed during drug treatment (Supplemental Figure 8A), and no substantial differences in the morphological characteristics of organs and tissues after treatment were detected, indicating that G C-K did not induce toxic effects on normal tissues at the experimental doses (Supplemental Figure 8B). These findings further substantiate the safety profile of G C-K in the context of GC treatment. Consistent outcomes were observed in the CDX model (Figure 6, A–F, and Supplemental Figure 8, C and D). Additionally, serum chemistry analysis revealed that key biomarkers for assessing posttreatment hepatic and renal function ALT and BUN remained within normal physiological ranges with no significant changes (Supplemental Figure 8, E and F). In summary, PARG KO markedly enhanced the sensitivity of GC to G C-K, providing a more in-depth experimental basis for the use of PARG as a potential therapeutic target.

### p21 governs G C-K sensitivity in PARG-deficient cancer.

**Figure 7:**
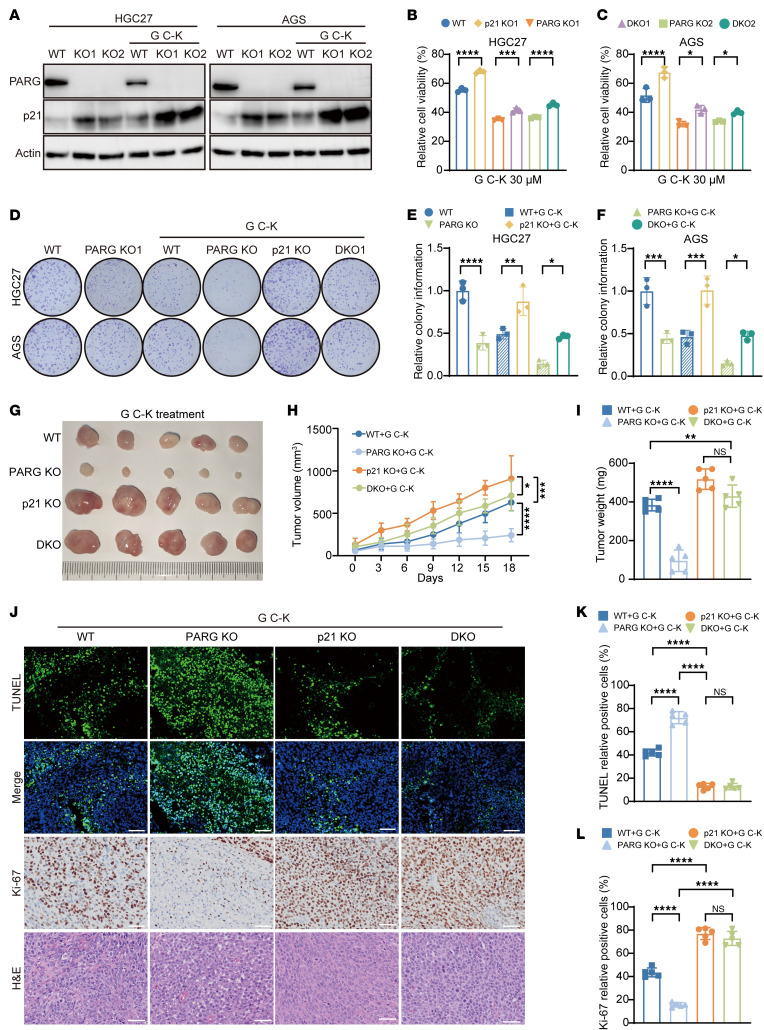
p21 governs G C-K sensitivity in PARG-deficient cancer. (**A**) G C-K enhances the regulatory effect of p21 in PARG KO gastric cancer cells. (**B**) A CCK-8 assay was used to detect the survival rate of PARG and p21 DKO HGC27 cells after G C-K treatment, *n* = 3. (**C**) A CCK-8 assay was used to detect the survival rate of PARG and p21 DKO AGS cells after G C-K treatment, *n* = 3. (**D**) Colony formation assay to detect colony formation in PARG and p21 DKO HGC27 and AGS cells after G C-K treatment. (**E**) Colony formation statistics of HGC27 and AGS cells after PARG or p21 DKO; *n* = 3. (**F**) G C-K treatment after PARG and p21 DKO AGS cell colony formation statistics, *n* = 3. (**G**) Tumor plots of the HGC27 xenograft model after G C-K treatment. (**H**) Tumor volume growth curve statistics of the HGC27 xenograft model after G C-K treatment, *n* = 5. (**I**) Tumor weight statistics of the HGC27 xenograft model after G C-K treatment, *n* = 5. (**J**) IHC staining of tumor tissues for TUNEL, Ki-67 and H&E staining; scale bar: 50 μm. (**K**) Statistical analysis of TUNEL-positive cells in tumor tissues, *n* = 5. (**L**) Ki-67–positive cell statistical graph, *n* = 5. (*P ≤ 0.05, **P ≤ 0.01, ***P ≤ 0.001, ****P ≤ 0.0001, NS, not significant, **H** by 2-way ANOVA, **B**, **C**, **E**, **F**, **I**, **K**, and **L** by 2-way ANOVA. Error bars represent the mean ± SD).

G C-K can induce cell cycle arrest, with p21 identified as one of the primary regulatory targets of this effect (52). Our transcriptomic profiling demonstrated that the core synergistic lethality of G C-K is executed through a p21-dependent pathway (Supplemental Figure 9, A and B). In addition, we examined the p21 protein levels in HGC27 and AGS cells after treatment with 30 μM G C-K for 48 hours via Western blotting (Figure 7A). The results demonstrated that the p21 protein level was elevated after PARG KO, and G C-K treatment further augmented the expression of p21, which was obviously increased compared with that in both WT and PARG KO cells. These findings suggest that G C-K remarkably amplifies the upregulation effect of p21 in PARG KO GC cells. To further validate these findings, we conducted a cellular activity assay via CCK-8 on p21 KO HGC27 and AGS cells treated with G C-K (Figure 7, B and C). The results showed that p21 KO could partially reverse the antiproliferative effect of G C-K. Furthermore, the results of the colony formation assay revealed that p21 KO partially rescued the colony formation ability after G C-K treatment (Figure 7, D–F). These findings collectively indicate that p21 plays an important role in the antitumor effects of G C-K. To further explore the antitumor effect of G C-K, we regulated p21 expression through a CDX model. The results showed that the growth rate of the tumors, as well as their volume and weight, was significantly inhibited by the PARG KO and G C-K treatments. However, p21 KO and DKO reversed the antitumor effects of G C-K (Figure 7, G–I). Subsequent IHC staining of tumor samples from the mice revealed that the percentage of TUNEL-positive cells was significantly increased and the percentage of Ki-67–positive cells was significantly decreased in the G C-K–treated PARG KO tumors, whereas the percentage of TUNEL-positive cells was significantly reduced and the percentage of Ki-67–positive cells was significantly increased in the p21 KO and DKO tumors (Figure 7, J–L).

## Discussion

GC is a globally prevalent malignancy with complex molecular pathogenesis (53). Despite surgical intervention as the primary treatment, outcomes remain unsatisfactory due to the high heterogeneity of disease, underscoring the urgent need for novel therapeutic strategies (6, 54).

Given the central role of poly(ADP-ribosyl)ation (PARylation) dynamics in genome stability, targeting this pathway has garnered substantial interest. However, while effective in other cancers, PARP inhibitors have shown limited efficacy in the treatment of GC in clinical trials (14). This highlights the need to explore alternative targets within the PARylation pathway. As the primary enzyme responsible for degrading PAR chains, PARG represents a promising candidate. Our study aimed to elucidate the functional role of PARG in GC, revealing its criticality in supporting tumor cell proliferation and cell cycle progression, thereby providing a strong rationale for PARG inhibition as a potential therapeutic approach.

PARG plays multifaceted oncogenic roles, as evidenced by synthetic lethal interactions with DNA damage response targets such as PARP and CHK1 (22), clinical associations with poor prognosis, and functional synergy with oncogenes such as HER2 (20). Despite these advances, the precise biological functions and mechanistic roles of PARG in GC pathogenesis has remained enigmatic until now.

Here, we find that PARG expression is markedly upregulated in GC tissues and that elevated PARG expression is correlated with unfavorable patient prognosis (Figure 1, A and B). Genetic ablation of PARG markedly inhibited GC cell proliferation, as confirmed by CCK-8 viability assays and colony formation analyses (Figure 1, C–F). Moreover, PARG deficiency induced substantial genomic instability in GC cells, characterized by chromosomal bridge formation, micronuclei accumulation, multinucleation, and chromosomal aberrations (Supplemental Figure 1, B–D, and Figure 2A). Cell cycle profiling through flow cytometry and FUCCI4 live-cell imaging demonstrated that PARG knockout induced G2/M phase arrest (Figure 1, H–K, Supplemental Figure 2, B and C, and Figure 3, A–D). Collectively, these findings position PARG as a promising therapeutic target for GC intervention strategies.

Importantly, our mechanistic results revealed a functional interaction between PARG and p21 involving the direct regulation of p21 protein level by PARG through the ubiquitin-proteasome pathway (Figure 2 and Figure 3, A and B). Furthermore, our findings indicated that knockout of PARG led to increased p21 PARylation level and a decreased p21 ubiquitination level (Figure 3, G–J). Research has demonstrated that PARG deficiency results in PARylation accumulation, facilitating the recruitment of the deubiquitinase USP7 and the subsequent stabilization of PARylated TOP1-DNA–protein crosslinks (TOP1-DPCs) (55). Therefore, we reason that PARG has the potential to influence the level of p21 ubiquitination by regulating the level of p21 PARylation. However, the potential for p21 regulation by alternative posttranslational modifications, such as phosphorylation and acetylation, in addition to PARylation and ubiquitination, and the coordination of these modifications with PARG function remain the subject for further exploration. Yet, polyubiquitination of the K48 linkage and the K161 and K163 residues of p21 was crucial for PARG to regulate the stability of the p21 protein (Supplemental Figure 5, B and C), corroborating and extending previous studies (7, 56).

p21 ubiquitination is regulated by E3 ubiquitin ligases such as SKP2, CRL4/Cdt2, CUL4B, RNF126, and UHRF2 (57). Through LC-MS and Co-IP experiments, we confirmed the pivotal role of PARG in regulating E3 ubiquitin ligase-mediated p21 ubiquitination (Supplemental Figure 5, D–I). A multitude of environmental (58) and dietary factors (59) contribute to the intricate molecular biological mechanisms underlying development of GC. While we elucidated the critical function of PARG in GC and its underlying molecular mechanism, further analysis may determine how PARG precisely regulates the interaction between E3 ubiquitin ligase and p21 and whether other cofactors are involved in this process. In an animal model, we verified that PARG KO inhibited proliferation in a manner dependent on the regulatory role of p21. Furthermore, the proliferative capacity of PARG and p21 DKO cells was significantly restored compared with that of PARG single-KO cells, suggesting that p21 plays a key role in PARG-mediated proliferation regulation (Figure 4). Besides its regulation of p21, whether PARG modulates cell cycle progression through other key regulatory proteins (including but not limited to Cyclin B1 and CDK1) during cell cycle distribution warrants further investigation. Notably, previous studies have reported that PARG inhibition can induce replication stress and ATR/CHK1 pathway activation, leading to S-phase perturbation in multiple tumor models (60). Our findings are consistent with the general notion that PARG plays an essential role in maintaining cell cycle integrity, while extending this understanding by revealing that PARG loss can also exert dominant effects at the G2/M checkpoint through p21 stabilization. These observations collectively suggest that PARG regulates the cell cycle at multiple levels, potentially influencing both S-phase and G2/M transitions. The specific checkpoint predominantly affected may vary depending on cell lineage, stress context, or mode of PARG perturbation, which warrants further investigation in future studies.

Interestingly, high-throughput screening of natural product libraries revealed that G C-K exhibited significantly enhanced cytotoxicity against PARG-deficient GC cells. Both in vitro and in vivo results demonstrated that PARG KO potentiates the antitumor efficacy of G C-K through p21 upregulation, providing compelling preclinical evidence for targeting PARG in combination with G C-K for GC therapy (Figures 5–7). While some studies have identified p21 as a primary regulatory target of G C-K, further research is necessary to elucidate the specific molecular mechanisms through which G C-K exerts its antitumor effects by regulating p21 expression, including its potential impact on p21 transcription, translation, or degradation processes.

Our collective results contribute to the body of knowledge surrounding GC pathogenesis. PARG inhibitors, including IDE161, ETX1977, and DAT2645, have been sanctioned by the FDA for clinical experimentation (61, 62) and thus current potential for GC clinical trials. Although the role of PARG in GC pathogenesis remains enigmatic, our data provide a solid experimental foundation for consideration of the combination of PARG inhibitors and G C-K in GC clinical trials.

**Figure 8:**
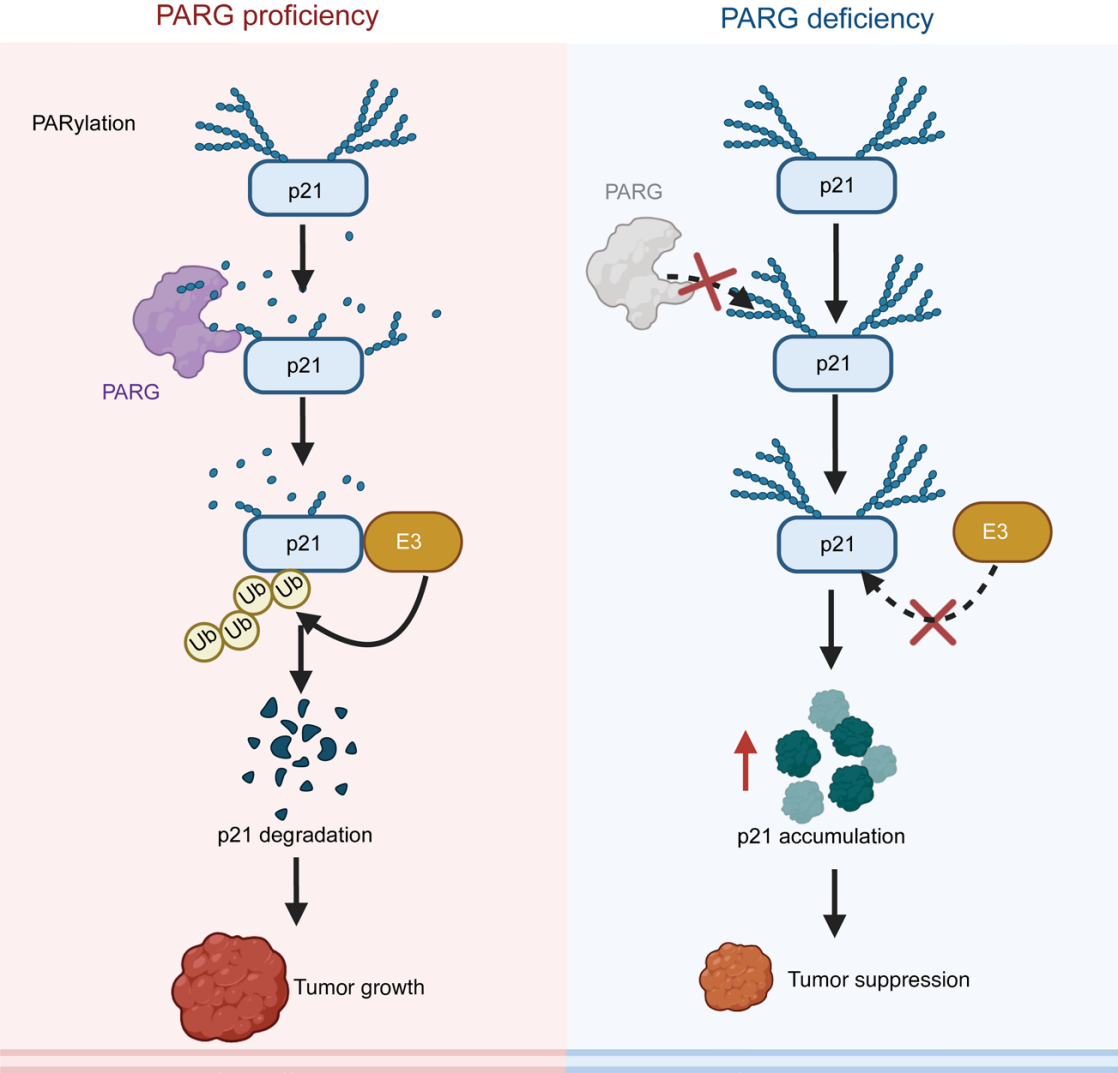
A mechanistic model of PARG-p21 axis in cancer progression. In the context of PARG proficiency, PARG engages in interaction with p21, resulting in dePARylation. This process subsequently enables the E3 ubiquitin ligase to interact with p21, thereby facilitating the ubiquitination and subsequent degradation of p21. This series of events culminates in the promotion of tumor growth. In the context of PARG deficiency, the PAR chains on p21 cannot be removed, spatial hindrance prevents E3 ubiquitin ligase from ubiquitinating p21 for degradation, and the accumulation of p21 leads to inhibition of tumor growth.

These findings support a model wherein PARG-mediated regulation of p21 expression drives GC progression (Figure 8). PARG activity dynamically modulates tumor growth by controlling p21 protein stability. Mechanistically, PARylation removal by PARG eliminates steric hindrance, facilitating E3 ubiquitin ligase binding to p21 and subsequent proteasomal degradation. The PARG-p21 axis is a regulatory pathway essential for GC progression. Furthermore, GC-K exhibits synergistic lethality with PARG deficiency, implicating PARG-p21 signaling as a critical regulator of cell proliferation. Collectively, these results establish a mechanistic framework for PARG’s biological function in GC and highlight its therapeutic potential.

## Methods

### Sex as a biological variable.

Sex was not considered as a biological variable in this study. GC samples from both female and male patients were included in the analysis. Male mice were used in all mouse studies for xenograft experiments, in accordance with previously established methodologies (63). While the in vivo studies were conducted exclusively in male mice, it is anticipated that the key mechanistic insights are applicable to both sexes, as the fundamental pathways driving GC progression are shared between male and female patients.

### Mice.

BALB/c mice were purchased from GemPharmatech and maintained in a specific pathogen-free facility. Experiments were initiated when the mice reached 6 weeks of age.

### Cell lines, drugs, reagents and antibodies.

The human cell lines AGS (Cat No. CRL-1739) and HEK293T (Cat No. CRL-5971) were obtained from the American Type Culture Collection (ATCC). The human cell lines HGC27 (Cat No. TCHu22), KATO-3 (Cat No. TCHu229), KYSE150 (Cat No. TCHu236), KYSE30 (Cat No. TCHu266), MDA-MB-231 (Cat No. TCHu227), Huh7 (Cat No. TCHu182), and HCT116 (Cat No. TCHu 99) were obtained from the National Collection of Authenticated Cell Cultures. The human cell lines MKN1 (Cat No. FH0320), NUGC3 (Cat No. FH1016), NUGC4 (Cat No. FH0480), MKN45 (Cat No. FH-MKN-45), and GES-1 (Cat No. FH0273) were ordered from Shanghai Fuheng Biological Co., Ltd. The human cell line AZ521 (Cat No.JCRB0061) was obtained from the Cell Bank RIKEN BioResource Center (Tsukuba, Japan). The GES-1, MKN1, MKN74, NUGC3, NUGC4, HGC27 and AGS cells were cultured at 37°C in a humidified atmosphere containing 5% CO_2_, and RPMI 1640 medium (Meilunbio, Cat. No. MA0215-2) supplemented with 10% fetal bovine serum (FBS, Cellmax, Cat. No. SA311.02). The MKN45, HEK293T,KYSE150, KYSE30,Huh7 and MDA-MB-231 cells were maintained in DMEM (Meilunbio, Cat. No. MA0212-2) supplemented with 10% fetal bovine serum. The KATO-3 cells were maintained in IMEM (Gibco, Cat. No. 31980030) supplemented with 10% fetal bovine serum. The AZ521 cells were maintained in MEM (cellmax, Cat. No. CGM110.05) supplemented with 10% fetal bovine serum, 1% Non-Essential Amino Acids (Gibco, Cat. No. 11140050), and 1 mM sodium pyruvate (Gibco, Cat. No. (Gibco, Cat. No. 11360-070). The HCT116 cells were maintained in McCoY’s 5A (Gibco, Cat. No. 31980030) supplemented with 10% fetal bovine serum. The drug genisteinide C-K (Proteintech, Cat. No. BM002089) was dissolved in dimethyl sulfoxide (DMSO; Meilunbio, Cat. No. PWL064) and stored at –20°C. For the relevant drug experiments, the cells were treated with various concentrations of the drug for a specified duration. Anti-PARG (#27808-1-AP, 1:10000), anti-RNF126 (#66647-1-Ig, 1:1000) and anti-Flag (#20543-1-AP, 1:1000) antibodies were purchased from Proteintech. Anti-p21 (#2947, 1:1000), anti-PAR (#83732S, 1:1000), and anti-SKP2 (#2652T, 1:1000) antibodies were purchased from Cell Signaling Technology. Anti-β-actin (#EM21002, 1:10000) antibody was purchased from HUABIO. Anti-HA (#ab9110, 1:10000) antibody was purchased from Abcam. Anti-Myc (#10015-M01, 1:1000) antibody was purchased from MabStar. The anti-Ub (#SC-8017, 1:1000) antibody was purchased from Santa Cruz. Anti-GFP (#YM8341, 1:5000) was purchased from Immunoway. Anti-Cdt2 (#YA2474, 1:500) was purchased from MCE. Anti-CCNA2 (#HA500287, 1:1000) and anti-CCNB1 (#ET1612-21, 1:1000) antibodies were purchased from Huaan Biotechnology Co., Ltd. Anti-rabbit IgG for IP (HRP) (#RA1008-00-A, 1:3000) was purchased from Vazyme. Goat Anti-Rabbit IgG (H&L) (#ASP1615, 1:10000) HRP and Goat Anti-Mouse IgG (H&L) HRP (#ASP1613, 1:10000) were purchased from Abcepta.

### Transfection and infection experiments and plasmids.

For the transfection and infection experiments, the target plasmids and packaging plasmids were transfected into HEK293FT cells via the transfection reagents Lipofectamine 3000 (Thermo, Cat. No. L3000015) and linear polyethyleneimine (PEI) (Polysciences, Cat. No. 23966-100 mg). Lentiviruses were collected 48 hours later and used to infect GC cells twice, 24 hours per infection. The infected cells were screened by treatment with puromycin for 36 hours, and the surviving cells were frozen and stored in liquid nitrogen for subsequent experiments. PARG sgRNA sequences were designed with the CRISPR guide design tool developed by Feng Zhang’s laboratory at the Broad Institute, Cambridge, Massachusetts, USA (https://zlab.squarespace.com/guide-design-resources) and inserted into the pLvx vector.

The sgRNA sequences are as follows: PARG sg1: 5′-GCTGGGCTCCGCGTCCACCG-3′; PARG-sg2: 5′-TTCGGCTCCGAGTACTTCGA-3′.

The plasmids pLV3-U6-CDKN1A(human)-sgRNA1-Cas9-Neo (#P67544), pLV3-U6-CDKN1A(human)-sgRNA2-Cas9-Puro (#P67518), pLL3.7m-Clover-Geminin(1-110)-IRES-mKO2-Cdt(30-120) (#P10263), pLL3.7m-mTurquoise2-SLBP(18-126)-IRES-H1-mMaroon1(#P1578), pCMV-SKP2(human)-3×HA-Neo(#P74730), pEnCMV-CDKN1A(human)-3×Myc-SV40-Neo (#P30729), pEnCMV-CDKN1A(human) (1-90aa)-3×Myc-SV40-Neo (#P30724), pEnCMV-CDKN1A(human) (1-140aa)-3×Myc-SV40-Neo (#P30719), pEnCMV-CDKN1A(human) (91-164aa)-3×Myc-SV40-Neo (#P30694) and pCMV-PARG(human)-Linker-EGFP-SV40-Neo (#P24472) were purchased from Miaoling Biotechnology Co., Ltd. (Wuhan, China). The plasmids pEnCMV-CDKN1A(human)-K16R-3×Myc-SV40-Neo, pEnCMV-CDKN1A(human)-(-K75R-3×Myc-SV40-Neo, pEnCMV-CDKN1A(human)-K141R-3×Myc-SV40-Neo, pEnCMV-CDKN1A(human)-K154R-3×Myc-SV40-Neo, pEnCMV-CDKN1A(human)-K161R-3×Myc-SV40-Neo, pEnCMV-CDKN1A(human)-K163R-3×Myc-SV40-Neo, pEnCMV-CDKN1A(human)- K2R(K161R,K163R)-3×Myc-SV40-Neo, pEnCMV-CDKN1A(human)- K4R(K16R,K154R,K161R,K164R)-3×Myc-SV40-Neo, pEnCMV-CDKN1A(human)-K6R(K16R,K75R,K141R,K154R,K161R,K164R)-3×Myc-SV40-Neo, pCMV-His-Ub, pCMV-PARG-Flag, pCMV-N1-Flag, pCMV-N1-3Myc, pCMV-PARG(human) (1-460aa)-Linker-EGFP-SV40-Neo, pCMV-PARG(human) (461-976aa)-Linker-EGFP-SV40-Neo, pCMV-PARG(human) (461-700aa)-Linker-EGFP-SV40-Neo, pCMV-PARG(human) (531-831aa)-Linker-EGFP-SV40-Neo, pCMV-PARG(human) (832-910aa)-Linker-EGFP-SV40-Neo, and pCMV-PARG-HA-TurboID were constructed by Ji Jing laboratory from the Hangzhou Institute of Medicine, Chinese Academy of Sciences. The plasmids pCMV-His-Ub (K6R), pCMV-His-Ub (K11R), pCMV-His-Ub (K27R), pCMV-His-Ub (K29R), pCMV-His-Ub (K33R), pCMV-His-Ub (K48R), and pCMV-His-Ub (K63R) were provided by Jiangjiang Qin from Hangzhou Institute of Medicine, Chinese Academy of Sciences.

### Bioinformatics analysis.

The gene expression levels of PARG in different types of tumors and normal tissues were obtained from TCGA. The significance of the expression difference was calculated via the Wilcoxon test. Differences in PARG gene expression levels between cancer and normal tissues were demonstrated via box plots and visualized using ‘ggplot2’ for presentation. The difference in overall survival (OS) between the 2 groups of patients was analyzed using Kaplan-Meier curves, and the significance of the survival distributions was assessed using the log-rank test in order to explore the correlation between the expression level of the PARG gene and patient survival. To explore the biological functions of PARG gene, we performed KEGG (Kyoto Encyclopedia of Genes and Genomes) functional enrichment analysis using the ‘Cluster-profiler’ software package. The screening criteria were |log2 fold change| > 1 and *P*-value < 0.05.

### IHC staining.

For each treatment group, mouse tumor tissues were fixed overnight at room temperature in 4% formalin and then embedded in paraffin, followed by serial ethanol gradient processing and paraffin embedding. Sections were cut at a thickness of 3 micrometers. For immunohistochemistry, sections were deparaffinized and rehydrated, then placed in a pressure cooker for antigen retrieval with sodium citrate buffer (10 mM, pH 6.0) for 3 minutes. After blocking with 5% normal goat serum, sections were incubated overnight at 4°C with the following primary antibody: anti-Ki-67 (Abcam, #15580). Apoptosis detection were performed with a TUNEL apoptosis assay kit (FITC-labeled green fluorescence) (Apigen, #C002), following the provided protocol. All sections were counterstained with hematoxylin, dehydrated, and mounted. Three sections per sample were examined, with five high-power fields randomly selected for analysis. ImageJ software was used to analyze the percentage of positive cells. Automated scoring was performed using the IHC Profiler plugin, followed by counting of positive and negative cells using the trainable Weka segmentation plugin.

### Western blot.

Proteins were extracted from the cells of interest by adding RIPA buffer and heating to induce denaturation, followed by sodium dodecyl sulfate-polyacrylamide gel electrophoresis (SDS-PAGE). After electrophoresis, the target proteins were transferred to a polyvinylidene fluoride (PVDF; Merck, Cat No. ISEQ00010) membrane. The membrane was then blocked with 5% skim milk for 1 hour at room temperature. After washing with Tris-buffered saline containing 0.05% Tween 20 (Tween 20; Meilbio, Cat No. 9005-64-5), the membrane was incubated with the primary antibody overnight at 4°C, washed with TBST, and then incubated with the corresponding secondary antibody for 1 hour at room temperature. Finally, chemiluminescent reagents (FDbio, Cat No. FD8020) were used to visualize the immunoreactive bands.

### RT-PCR.

Total RNA from cells was extracted via TRIzol (Vazyme, Cat No. R401-01), and complementary DNA (cDNA) was generated via a reverse transcription kit (Vazyme, Cat No. R212-02). Transcript expression levels were measured on a CFX96 Real-Time System (Bio-Rad) via the SYBR Green Premix Pro Taq HS qPCR Kit (AG, Cat No. AG11718). Relative gene expression was quantified via the 2^–ΔΔCt^ method and normalized to β-actin for related gene expression analysis.

Primer sequences for the target gene *CDKN1A* were: forward, 5′-TCCAGCGACCTTCCTCATCCAC-3′; reverse, 5′-TCCATAGCCTCTACTGCCACCATC-3′. Primer sequences for the reference gene β*-actin* were: forward, 5′-GAGCTACGAGCTGCCTGAC-3′; reverse, 5′-GGTAGTTTCGTGGATGCCACAG-3′.

### Immunoprecipitation.

In this study, AGS and HEK293T cells were transfected with the target plasmid. Total protein was subsequently extracted with ice-cold RIPA lysis buffer. Next, these proteins were incubated with anti-Flag (Proteintech, #20543-1-AP, 800ug:2 μL), anti-Myc (ABclonal,# AE070, 800ug:1 μL) tag antibodies, with IgG antibodies or anti-GFP magnetic beads (Beyotime, P2132) at a constant speed (60 r/min) overnight at 4°C. After that, the Co-IP complexes were precipitated with Protein A/G magnetic beads (Thermo, Cat No 88802), and the beads were washed 3 times with RIPA lysis buffer. The complex incubated with GFP magnetic beads was directly washed 3 times with RIPA lysis buffer. Finally, the immunoprecipitated complexes were detected via Western blot analysis.

### Cell proliferation assay.

For analysis of the proliferation of cells, 1 × 10^3^ cells were cultured in 96-well plates for 7 days. A Cell Counting Kit-8 (CCK-8) assay kit (Meilunbio, Cat. No. MA0218-3) was used to detect cell viability and growth curves. All experiments were independently performed three times.

### Colony formation assay.

HGC27 and AGS cells were seeded in 6-well plates at a density of 500 cells per well, with each experimental group receiving the designated pretreatment. Following a 14-day incubation period at 37°C with 5% CO_2_, the cells were gently washed 3 times with phosphate-buffered saline (PBS) (Cinery, #CR-20012-250ML) and fixed with 4% paraformaldehyde (Servicebio, #G1101-500ML) for 30 minutes at room temperature. After 3 additional PBS washes, the colonies were stained with 0.4% (w/v) crystal violet solution (Beyotime Biotechnology, #C0121) for 30 minutes. Stained colonies were photographed and quantified to determine cloning efficiency. All experiments were independently performed three times.

### Cell apoptosis assays.

The cells were seeded in 6-well plates at a density of 1 × 10^4^ cells per well and treated according to the experimental conditions for 48 hours. Following treatment, the cells were harvested, washed with phosphate-buffered saline (PBS), and centrifuged at 300 × *g* for 5 minutes to pellet the cells. The supernatant was carefully removed, and the cells were resuspended in 100 μL of 1× Annexin V binding buffer. The cells were subsequently stained with 5 μL of FITC-conjugated Annexin V and 5 μL of propidium iodide (PI) (Beyotime Biotechnology, C1062M) for 15 minutes at room temperature in the dark. Apoptotic cells were quantified via a Beckman Coulter CytoFLEX flow cytometer (Beckman Coulter).

### Cell cycle assays.

HGC27 and AGS cells were seeded in 6-well plates at a density of 1 × 10^4^ cells per well and treated according to the experimental conditions for 48 hours. Following trypsinization, harvested cells were resuspended in ice-cold phosphate-buffered saline (PBS) and centrifuged at 300 × *g* for 5 minutes at 4°C. The supernatant was carefully removed, and the cells were fixed in pre-chilled 70% ethanol overnight at 4°C. After fixation, the cells were pelleted by centrifugation (1000 rpm, 5 minutes, 4°C), washed once with PBS, and centrifuged again under identical conditions. For DNA staining, the cells were resuspended in 0.5 mL of staining solution containing 50 μg/mL propidium iodide (PI; Beyotime Biotechnology, C1052) and 100 μg/mL RNase A, which was prepared according to the manufacturer’s protocol. The cell suspension was incubated at 37°C for 30 minutes in the dark. Cell cycle analysis was performed using a Beckman Coulter CytoFLEX flow cytometer. The acquired data were analyzed via FlowJo software (version 10.8.1; FlowJo LLC, USA).

### FUCCI4 live cell imaging.

Cells were infected using 2 double cis-trans lentiviruses containing EMCV IRES, pLL3.7m-Clover-Geminin (1-110)-IRES-mKO2-Cdt (30-120) and pLL3.7m-mTurquoise2-SLBP (18-126)-IRES-H1-mMaroon1 (64). Cells were cultured in glass-bottomed 96-well plates infected with packaged viruses. Cells were incubated in 2 mM thymidine for 18 hours, fresh medium for 9 hours, and again in 2 mM thymidine for 15 hours with the following excitation (ex) and emission (em) filters: cyan, ex 440/10 nm (Chroma), em 472/30 nm (Semrock); green, ex 490/10 nm (Chroma), and em 525/30 nm (Semrock); orange, ex 545/10 nm (Omega), em 575/25 nm (Chroma); far-red, ex 610/10 nm (Omega), em 665/65 nm (Chroma) imaging; and imaging at 37°C using an environmental control system (ImageXpress Micro) at 37°C and 5% CO_2_. Images were acquired every 30 minutes.

### Chromosomal aberration analysis.

HGC27 and AGS cells were cultured in 10 cm dishes until reaching approximately 90% confluence. The culture medium was replaced with fresh medium containing 0.2 μg/ml colchicine (Beyotime Biotechnology, ST1173), and the mixture was incubated for 4 hours at 37°C in a 5% CO_2_ atmosphere. Following treatment, both adherent and floating cells were collected by trypsinization and centrifugation at 200 × *g* for 5 minutes. The cell pellet was resuspended in 0.075 M KCl hypotonic solution (Beyotime Biotechnology, ST340) and incubated for 30 minutes at 37°C. Then, the cells were fixed in a fixative solution prepared in a 3:1 ratio of methanol (Chron; Cat No. 67-56-1) to acetic acid (Gtech; Cat No. 64-19-7). Fixed cell suspensions were dropped onto pre-chilled glass slides and air-dried overnight. Chromosome spreads were stained with 10% Giemsa solution (Sigma-Aldrich, 51811-82-6) in phosphate buffer (pH 6.8) for 10 minutes, rinsed gently with distilled water, and air-dried. Metaphase chromosomes were analyzed via a Zeiss LSM 700 confocal microscope with a 100× oil immersion objective. For each experimental condition, a minimum of 50 well-spread metaphase cells were evaluated for chromosomal abnormalities.

### Proximity labeling with TurboID and LC-MS/MS.

The PARG-HA-TurboID fusion plasmid was constructed using standard molecular cloning techniques. HEK293T cells were transfected with either the experimental construct or empty vector control using polyethylenimine (PEI) transfection reagent (Polysciences, 23966) at a 3:1 PEI:DNA ratio. Following 24–36 hours of incubation at 37°C with 5% CO_2_, the experimental cells were treated with 100 μM biotin (Sigma-Aldrich, B4501) in DMEM for 1 hour to enable proximity labeling. The labeling reaction was terminated by placing the cells on ice. The cells were harvested by scraping and lysed in RIPA buffer containing protease inhibitors. TurboID-mediated biotinylation was confirmed by western blot analysis using streptavidin-HRP (1:5000; Thermo Fisher, 21130). Biotinylated proteins were enriched using streptavidin-coated magnetic beads (Pierce, 88817) according to the manufacturer’s protocol. The bound proteins were eluted and subjected to tryptic digestion for subsequent LC-MS/MS analysis to identify PARG-interacting proteins.

### Structure modeling.

PARG (NP_003622.2) and p21 (NP_000380.1) were modeled using the AlphaFold3 (AF3) server (https://alphafoldserver.com/) (65). PyMOL was used to analyze the structure models and prepare the figures.

### Animal experiments.

Four- to 5-week-old male athymic BALB/c mice were maintained under specific pathogen-free (SPF) conditions. Previously generated patient-derived GC tissues or 1×10^7^ HGC27 cells suspended in 100 μL of PBS were subcutaneously implanted into the right flank of each mouse. When the tumors reached 1–2 cm^3^, they were excised, dissected into 2 mm^3^ fragments, and re-implanted into secondary recipient mice. The treatment groups received intraperitoneal injections of drugs (30 mg/kg) every 72 hours. Tumor volume (V = ½ × width^2^ × length) and body weight were monitored triweekly. The final procedures included CO_2_ euthanasia followed by collection of tumors and major organs for immunohistochemical analysis.

### Automated detection methods for Serum Alanine Aminotransferase (ALT) and Blood Urea Nitrogen (BUN).

Blood samples were collected and centrifuged at 3000 rpm for 10 minutes to obtain clear serum. ALT assay kits (Rayto Life and Analytical Sciences Co., Ltd, # R01502) and BUN assay kits (Rayto Life and Analytical Sciences Co., Ltd, # R02902) were equilibrated at room temperature (18–25°C) for 15–30 minutes and prepared according to the manufacturer’s instructions. The measurements were then performed using a fully automated biochemical analyzer (Rayto Life and Analytical Sciences Co., Ltd, Chemray 240).

### Statistics.

The data are presented as mean ± SD from independent experiments. Statistical significance was determined via 2-tailed Student’s *t* test for comparisons between 2 groups or 2-way ANOVA for multiple group comparisons with comparable variances. *P* < 0.05 was considered statistically significant. All experiments included at least 3 independent biological replicates. Statistical analyses were performed via GraphPad Prism 9 (version 9.0.0; GraphPad Software).

### Study approval.

All the study procedures, including PDX model generation, animal care, and handling were approved by the Ethics Committee of Zhejiang Cancer Hospital (approval No. IRB-2023-538) and the Animal Protection and Use Committee of the Institute of Basic Medical Sciences and Oncology, Chinese Academy of Sciences (approval no. AP2024-12-0379). All procedures involving animals were approved by the Animal Care and Use Committee of the Hangzhou Institute of Medicine, Chinese Academy of Sciences.

### Data availability.

The tumor transcriptomic data used in this study were obtained from The Cancer Genome Atlas (TCGA), a publicly available cancer genomics program jointly supported by the National Cancer Institute and the National Human Genome Research Institute. The data are hosted by the National Cancer Institute’s Center for Cancer Genomics and can be accessed at: https://www.cancer.gov/ccg/research/genome-sequencing/tcga All R scripts used for data processing, statistical analysis, and visualization have been made publicly available on GitHub at: https://github.com/dmcc816/dmcc.git (commit ID 5621fd8f4879b18a79452f00ba90b065507de426). The raw sequence data reported in this paper have been deposited in the Genome Sequence Archive (Genomics, Proteomics & Bioinformatics 2025) in National Genomics Data Center (Nucleic Acids es 2025), China National Center for Bioinformation / Beijing Institute of Genomics, Chinese Academy of Sciences (GSA-Human: HRA014714) that are publicly accessible at https://ngdc.cncb.ac.cn/gsa-human The raw data from proximity tagging coupled with mass spectrometry analysis reported in this paper have been deposited in the Genome Sequence Archive (Genomics, Proteomics & Bioinformatics 2025) in National Genomics Data Center (Nucleic Acids es 2025), China National Center for Bioinformation / Beijing Institute of Genomics, Chinese Academy of Sciences (OMIX: OMIX013052) that are publicly accessible at https://ngdc.cncb.ac.cn/omix Data will be available upon request. All Supporting data values are compiled in the Supporting Data Values file.

## Author contributions

Conceptualization: ZY, JJ, and XC. Investigation: Y Hu, QB, Y Huang, YW, XZ, JN, and YM. Visualization: MD, YL, and ZZ. Data curation: Y Hu, HH, DZ, YZ, and CZ. Methodology: Y Hu, BW, RL, Y He, and ML. Writing—original draft preparation: Y Hu. Writing—review and editing: ZY, JJ, YS, QW, and JAT. Supervision: ZY and JJ. Funding acquisition: ZY and JJ. All authors have read and agreed to the published version of the manuscript.

## Funding support

This work is the result of NIH funding, in whole or in part, and is subject to the NIH Public Access Policy. Through acceptance of this federal funding, the NIH has been given a right to make the work publicly available in PubMed Central.

- The National Natural Science Foundation of China (Grant No. 82473195).
- The Natural Science Foundation of Zhejiang Province (Grant No. LTGY23H160018).
- The Zhejiang Medical and Health Science and Technology Program (Grant No. 2024KY789).
- The Zhejiang Provincial Disease Control and Prevention Science and Technology Key Program (Grant No. 2026JKZ035).
- The National Research Center for Translational Medicine at Shanghai Program [Grant No. NRCTM(SH)-2025-07].
- The Beijing Science and Technology Innovation Medical Development Foundation (Grant No. KC2023-JX-0270-07).
- Guangxi Key Laboratory of Early Prevention and Treatment for Regional High Frequency Tumor Funding (Grant No. GKE-KF-202403).
- The National R&D Program of China (2023YFC3403400).
- The National Natural Science Foundation of China (32271485).
- The Natural Science Foundation of Zhejiang Province (YXD23H0302).
- The National Cancer Institute P01 CA092584 and Robert Welch Chemistry Chair G-0010 to JAT.

## Supplementary Material

Supplemental data

Unedited blot and gel images

Supporting data values

## Version 1 01/15/2026

In-Press Preview

## Version 2 03/02/2026

Electronic publication
