# Supplementary material for: Poly(ADP-ribose) glycohydrolase enforces p21 degradation via dePARylation to promote gastric cancer progression
Source: J Clin Invest. 2026 Jan 15;136(5):e195538. doi: 10.1172/JCI195538 (PMC13067944; doi:10.1172/JCI195538)
Supplement: Supplemental data [file jci-136-195538-s293.pdf]

Poly(ADP-ribose) glycohydrolase enforces p21 degradation via dePARylation to promote gastric cancer progression

Yangchan Hu[1,2], Qimei Bao[1], Yixing Huang[1,3], Yan Wang[1,4], Xin Zhao[1], Junjun Nan[1,2], Yuxin Meng[1,2], Mingcong Deng[1], Yuancong Li[1,2], Zirui Zhuang[1,5], Hanyi He[1], Dan Zu[1,6], Yuke Zhong[1], Chunkai Zhang[1], Bing Wang[1,2], Ran Li[1,2], Yanhua He[1], Qihan Wang[7], Min Liu[8], John A. Tainer[8], Yin Shi[3], Xiangdong Cheng[1,9,10,*], Ji Jing[1,9,10,*] and Zu Ye[1,9,10,11,12,*]

[1] Zhejiang Cancer Hospital, Hangzhou Institute of Medicine (HIM), Chinese Academy of Sciences, Hangzhou, Zhejiang, China

[2]College of Pharmaceutical Science, Zhejiang University of Technology, Hangzhou 310014, China

[3]Department of Biochemistry and Department of Pulmonology, Children's Hospital, Zhejiang University School of Medicine, National Clinical Research Center for Children and Adolescents' Health and Diseases, Hangzhou 310058, China.

[4]Collaborative Innovation Center of Yangtza River Delta Region Green Pharmaceuticals, Zhejiang University of Technology, Hangzhou 310014, China

[5]School of Molecular Medicine, Hangzhou Institute for Advanced Study, University of Chinese Academy of Sciences (UCAS), Hangzhou 310024, China

[6]School of Life Sciences, Tianjin University, Tianjin 300100, China.

[7]College of Biological Sciences, University of California, Davis, CA 95616, USA

[8]Departments of Molecular and Cellular Oncology and Cancer Biology, The University of Texas MD Anderson Cancer Center, Houston, TX 77030, USA.

[9]Key Laboratory of Prevention, Diagnosis and Therapy of Gastrointestinal Cancer of Zhejiang Province, Hangzhou 310022, China.

[10]Zhejiang Provincial Research Center for Upper Gastrointestinal Tract Cancer, Zhejiang Cancer Hospital, Hangzhou 310022, China.

[11]Guangxi Key Laboratory of Early Prevention and Treatment for Regional High Frequency Tumor, Nanning 530021, China

[12]Key Laboratory of Early Prevention and Treatment for Regional High Frequency Tumor (Guangxi Medical University), Ministry of Education, Nanning 530021, China

*Correspondence:

Zu Ye, Zhejiang Cancer Hospital, Hangzhou Institute of Medicine (HIM), Chinese Academy of Sciences, Hangzhou, Zhejiang 310022, China. E-mail: yezuqscx@zju.edu.cn

Ji Jing, Zhejiang Cancer Hospital, Hangzhou Institute of Medicine (HIM), Chinese

Academy of Sciences, Hangzhou, Zhejiang 310022, China. E-mail: jingji@him.cas.cn
Xiangdong Cheng, Zhejiang Cancer Hospital, Hangzhou Institute of Medicine (HIM), Chinese Academy of Sciences, Hangzhou, Zhejiang 310022, China. E-mail: yezuqscx@gmail.com

Supplementary information

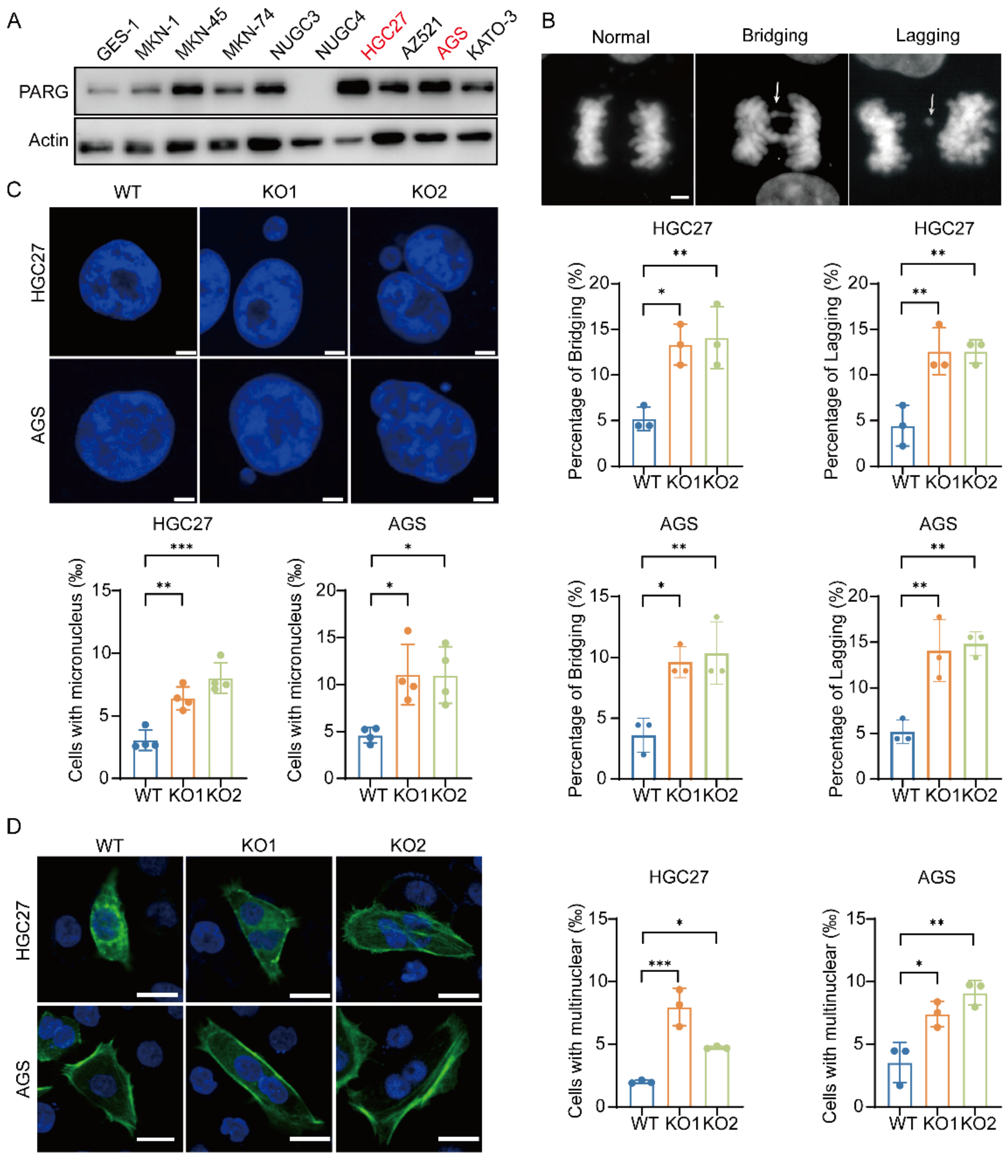

Supplementary Figure 1. Downregulation of PARG expression induces genomic instability. (**A**) Protein expression of PARG in gastric cancer cell lines. (**B**) Late chromosome division, from left to right, shows the normal chromosomes, chromosome bridges, and late lagging chromosomes in late division, scale bar = 3 μm. Quantification of chromosome bridges and late lagging chromosomes in late division in WT and PARG KO HGC27 and AGS cells, n = 50. (**C**) Imaging of micronucleus formation in WT and PARG KO HGC27 and AGS cells via confocal rotary disk microscopy; scale bar = 2 μm. Statistical plot of micronucleus formation in WT HGC27 and PARG KO AGS cells, n = 4, > 100 cells. (**D**) Imaging of multinuclei formation in WT and PARG KO HGC27 and AGS cells. Green fluorescence represents the Lifeact-pEGFP-labeled actin cytoskeleton, and blue fluorescence represents DAPI, scale bar = 20 μm. Statistical graph of multinuclei formation in WT and PARG KO HGC27 and AGS cells per thousand, n = 3. (**P* ≤ 0.05, ***P* ≤ 0.01, ****P* ≤ 0.001 by one-way ANOVA. Error bars represent the mean ± SD)

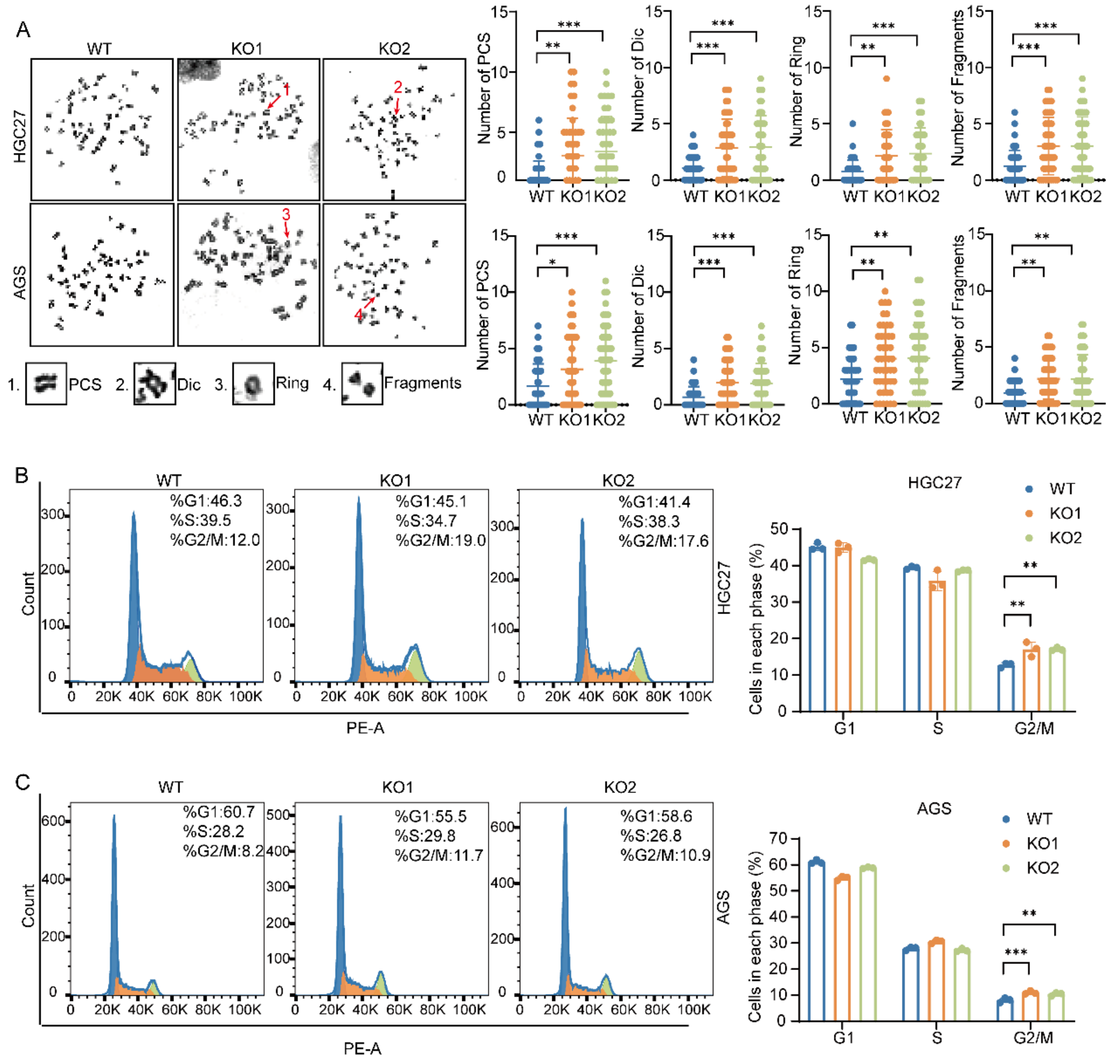

Supplementary Figure 2. PARG knockout induces chromosomal aberrations and cell cycle arrest at the G2/M phase. (**A**) PARG KO HGC27 and AGS cells presented increased chromosomal aberrations, with 1-4 indicating premature sister chromatid separation (PCS), double-stranded chromosome (Dic), ring chromosome (Ring), and fragmentation (Fragments), respectively. Statistical plots of chromosomal aberrations in WT HGC27 and PARG KO AGS cells (PCS, Dic, Ring, Fragments), n = 47. (**B**) Cell cycle distribution diagrams and statistical charts for each phase in wild-type and PARG KO HGC27 cells detected by a serum starvation assay, n = 3. (**C**) Cell cycle distribution diagrams and statistical charts for each phase in wild-type and PARG KO AGS cells detected by a serum starvation assay, n = 3. (***P* ≤ 0.01, ****P* ≤ 0.001 by one-way ANOVA. Error bars represent the mean ± SD)

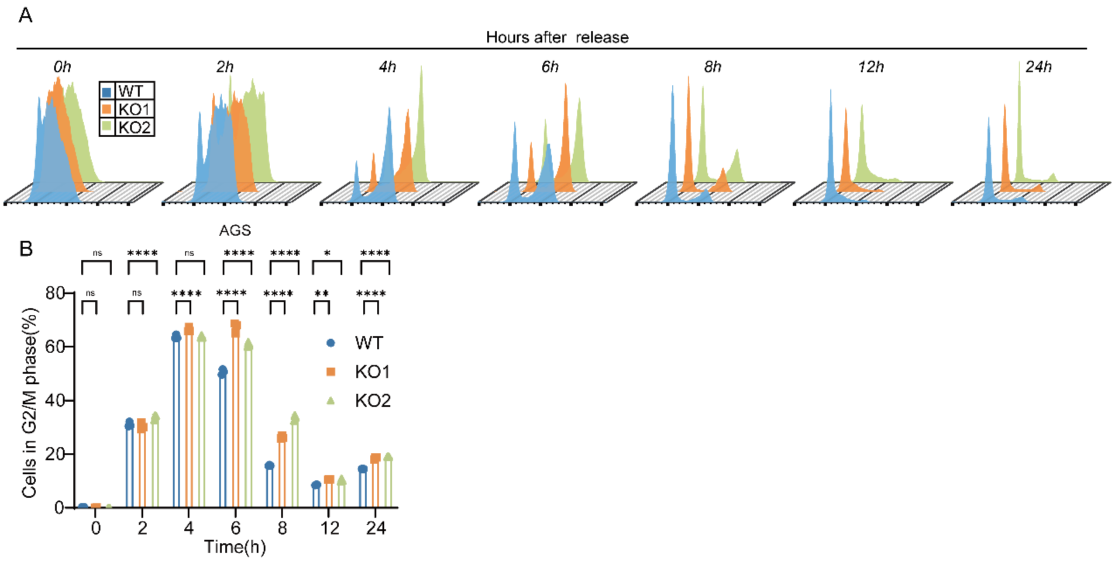

Supplementary Figure 3. PARG knockout induces cell cycle arrest at the G2/M phase. (**A**) Cell cycle distribution of WT and PARG KO AGS cells detected by the thymidine double-block assay. (**B**) Statistics of AGS cells in G2/M phase, n = 3. (**P* ≤ 0.05, ***P* ≤ 0.01, ****P* ≤ 0.001, *****P* ≤ 0.0001, ns represents *P* > 0.05 by two-way ANOVA. Error bars represent the mean ± SD)

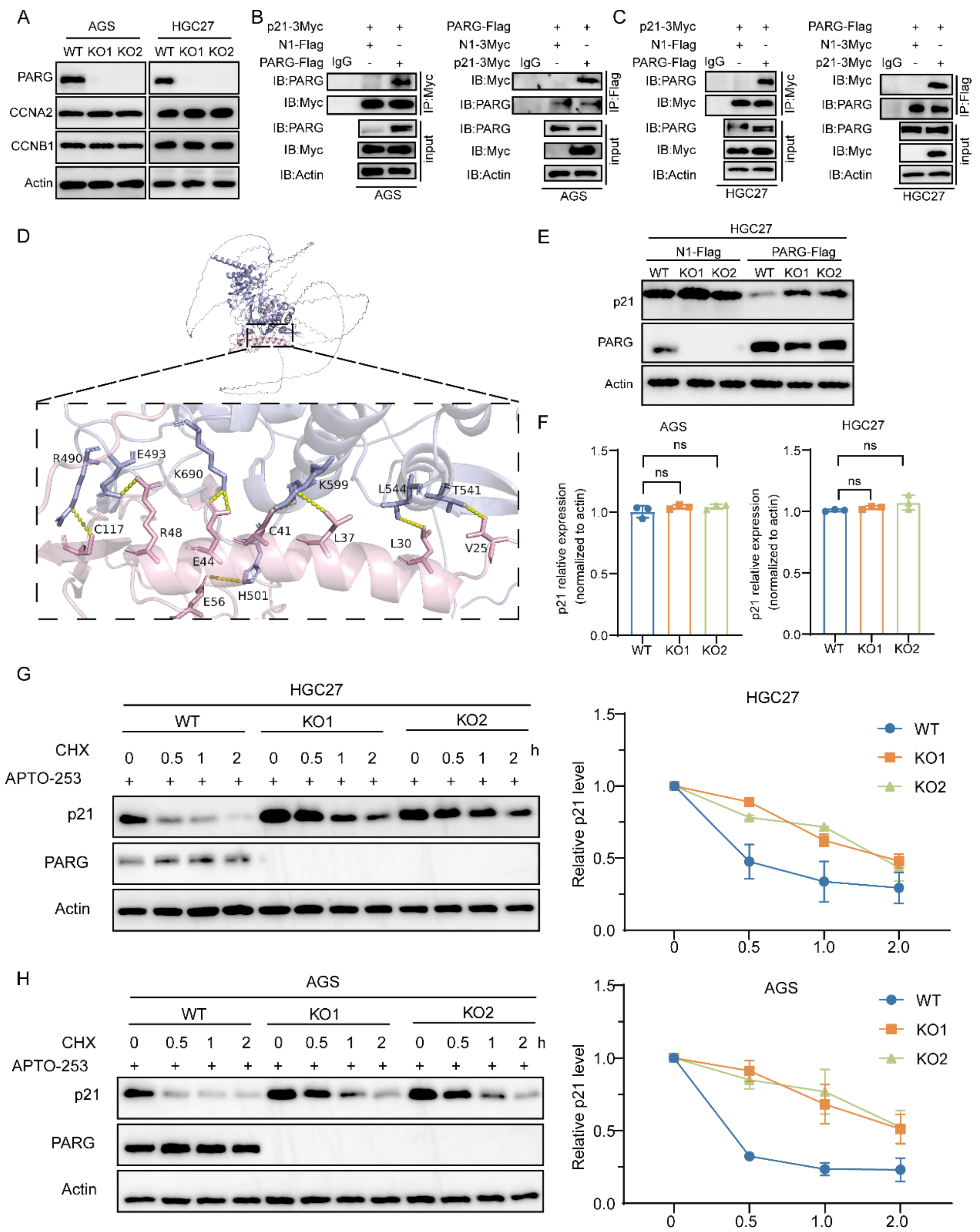

Supplementary Figure 4. PARG interacts with p21 and promotes its degradation via the proteasome pathway. (**A**) Western blot detection of CCNA2 and CCNB1 protein expression in WT and PARG KO HGC27 and AGS cells. (**B**, **C**) PARG-flag and p21-3Myc plasmids were co-transfected into AGS and HGC27 cells, and total cell lysates were immunoprecipitated with anti-flag and anti-Myc antibodies, respectively. Then, anti-Myc and anti-PARG antibodies were used to detect the immunoprecipitates, and anti-PARG and anti-Myc antibodies were used to detect the success of the experiments. (**D**) Predicted molecular model of the PARG (purple) interacting with p21 (pink). (**E**) The PARG-Flag plasmid was transfected into WT and PARG KO HGC27 cells, followed by Western blot analysis of PARG-Flag transfection efficiency and its

effect on p21 protein expression. (**F**) RT–PCR was used to detect p21 protein transcript levels in WT and PARG KO AGS and HGC27 cells. The p21 transcript levels were normalized to actin. (**G**, **H**) Following a 4-hour treatment period with 5 μM APTO-253, 200 μg/mL CHX was added at 2, 1, and 0.5 hours, respectively, prior to conducting Western blot analysis. Quantification of p21 expression in AGS and HGC27 cells. WT, KO1, and KO2 cells were normalized to their corresponding CHX-treated 0 h samples. (ns represents $P > 0.05$ by one-way ANOVA. Error bars represent the mean ± SD)

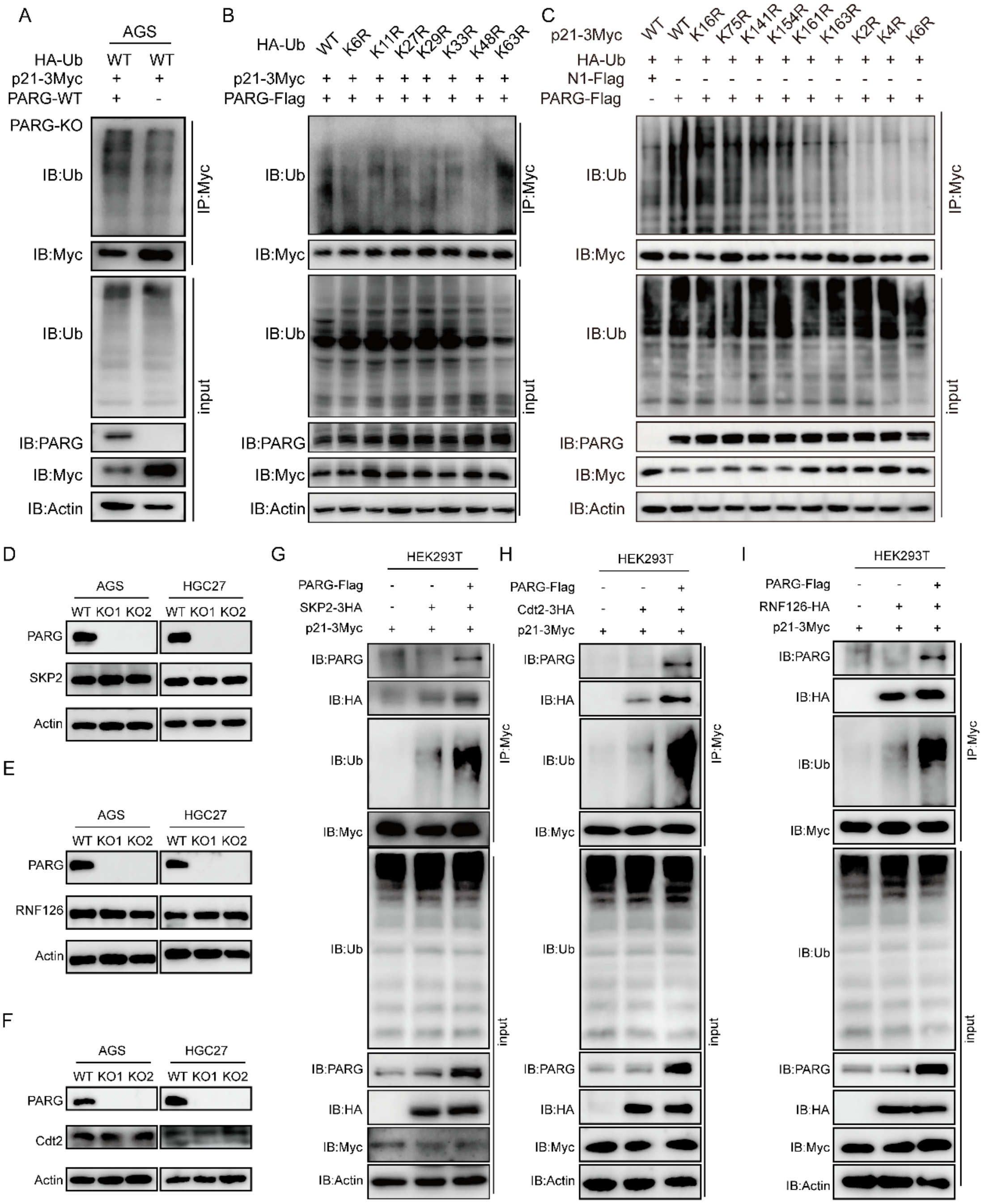

Supplementary Figure 5. PARG promotes the interaction between E3 ubiquitin ligase and p21 to regulate p21 ubiquitination. (**A**) p21-3Myc and HA-Ub plasmids were transiently transfected into AGS cells, and the transfected AGS cells were treated with MG132 for 6 h to harvest proteins. The ubiquitinated p21 protein was pulled down with an anti-Myc antibody and immunoblotted with an anti-Ub antibody. (**B**) K48-linked polyubiquitination plays a key role in PARG regulation of p21 ubiquitination. (**C**) Residues K161 and 163 of p21 play key roles in PARG regulation of p21 ubiquitination. (**D**, **E**, **F**) Western blot detection of SKP2, Cdt2 and RNF126 protein expression in WT and PARG KO HGC27 and AGS cells. (**G**) PARG promotes

the interaction between the E3 ubiquitin ligase SKP2 and p21, which promotes the ubiquitination of p21.(**H**) PARG promotes the interaction between the E3 ubiquitin ligase Cdt2 and p21, which promotes the ubiquitination of p21. (**I**) PARG promotes the interaction between the E3 ubiquitin ligase RNF126 and p21, which promotes the ubiquitination of p21.

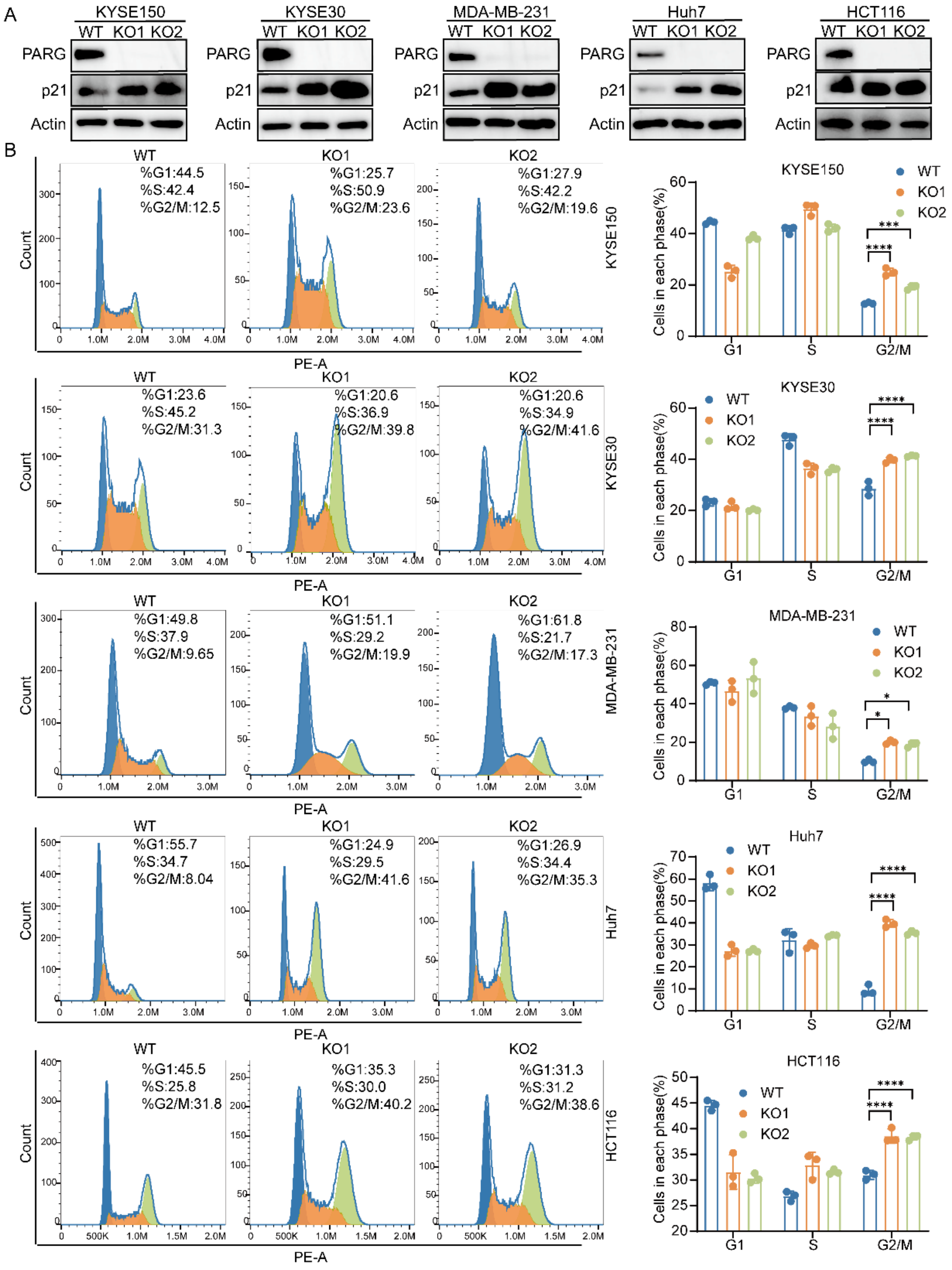

Supplementary Figure 6. Knockout of PARG induces G2/M phase arrest in esophageal cancer, breast cancer, liver cancer, and colorectal cancer. (**A**)Western blot detection of p21 protein expression in WT and PARG KO KYSE150, KYSE30, MDA-MB-231, Huh7 and HCT116 cells. (**B**) Cell cycle distribution diagrams and statistical charts for each phase in wild-type and PARG KO KYSE150, KYSE30, MDA-MB-231, Huh7 and HCT116 cells detected by a serum starvation assay, n = 3. (**P* ≤ 0.05, ****P* ≤ 0.001, *****P* ≤ 0.0001 by two-way ANOVA. Error bars represent the mean ± SD)

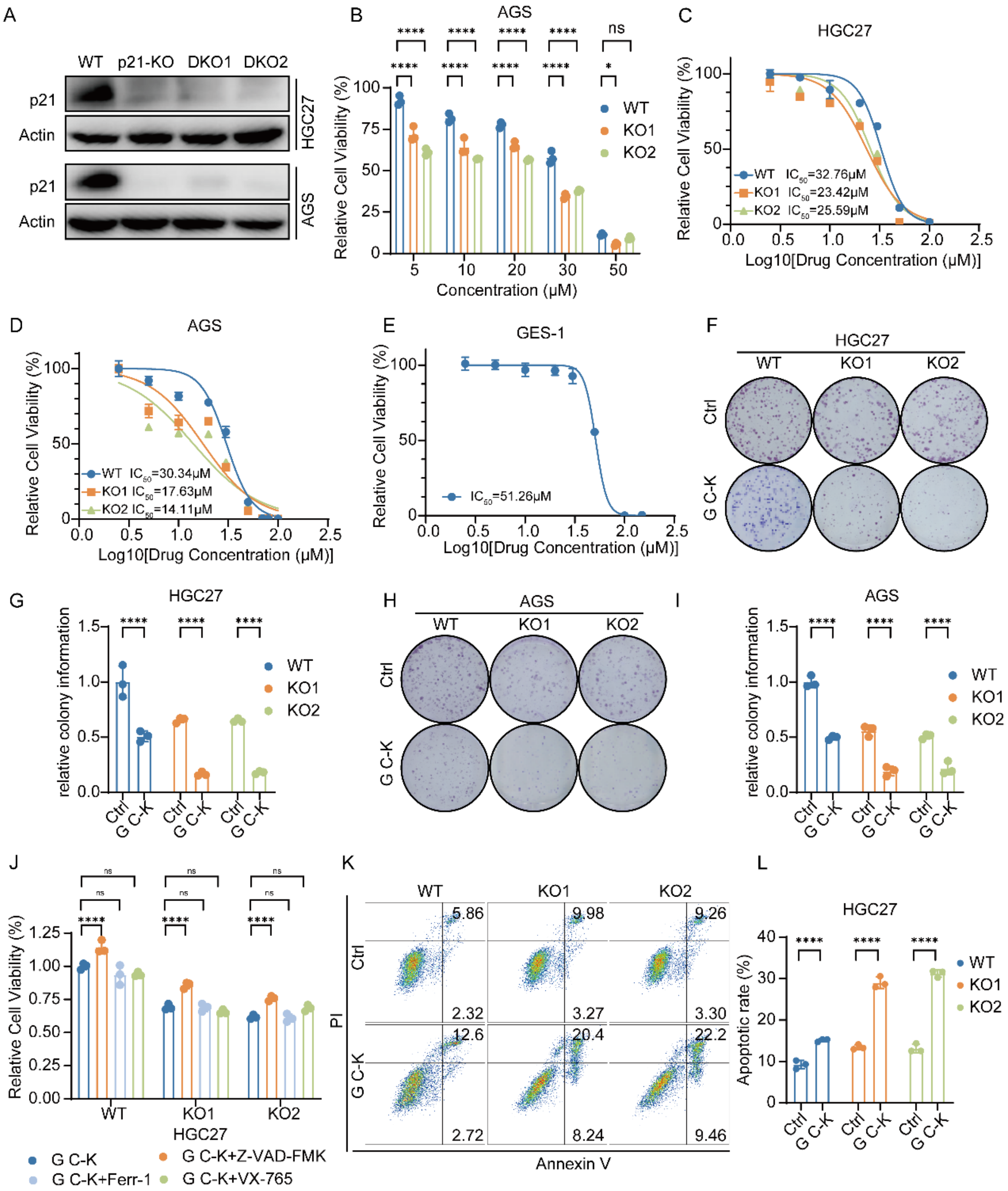

Supplementary Figure 7. PARG loss increases the sensitivity of GC cells to G C-K (**A**) Construction of PARG and p21 DKO cell lines. (**B**) CCK-8 assay to detect the survival of G C-K-treated WT and PARG KO AGS cells, n = 3.(**C**) IC_{50} of G C-K in WT and PARG KO HGC27 cells. (D) IC_{50} of G C-K in WT and PARG KO AGS cells (**E**) IC_{50} of G C-K in GES-1 cells (**F**) A colony formation assay was performed to generate colony formation plots of WT and PARG KO HGC27 cells after G C-K treatment. (**G**) Statistical plots of colony formation by WT and PARG KO HGC27 cells after G C-K treatment, n = 3. (**H**) Colony formation assay to detect G C-K-treated WT and PARG KO AGS cell colony formation. (**I**) G C-K-treated WT and PARG KO AGS cell colony formation statistics, n = 3. (**J**) A CCK-8 assay was used to detect cell viability of HGC27 cells after G C-K treatment, n = 3. Z-VAD-FMK:10 μM; Ferr-1:2 μM; VX-765:1 μM. (**K**) Annexin-V-PI double-staining assay was used to detect the degree of apoptosis in WT and

PARG KO HGC27 cells under the effect of G C-K. (**L**) Histogram of the percentage of apoptotic WT and PARG KO HGC27 cells and the statistics of the Annexin V-positive cell population, n = 3. (*$P \leq 0.05$, ***$P \leq 0.001$, ****$P \leq 0.0001$, ns represents $P > 0.05$ by two-way ANOVA. Error bars represent the mean ± SD)

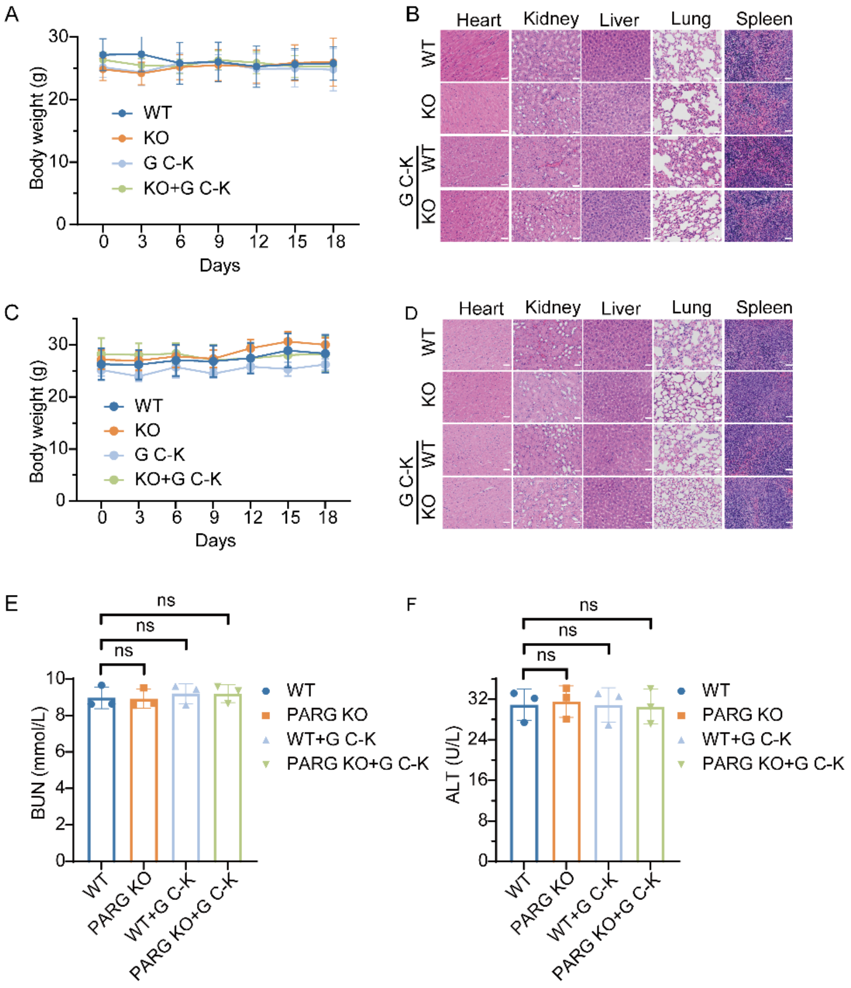

Supplementary Figure 8. G C-K has a favorable safety profile in vivo. (**A**) Weight curve of PDX tumor-bearing mice during G C-K treatment, n = 5. (**B**) H&E staining of major organ tissues (heart, liver, spleen, lung, and kidneys) from each group of PDX tumur-bearing mice; scale bar = 50 μm. (**C**) Weight curve of CDX tumor-bearing mice during G C-K treatment, n = 5. (**D**) H&E staining of major organ tissues (heart, liver, spleen, lung, and kidneys) from each group of CDX tumor-bearing mice, scale bar = 50 μm. (**E**) The ALT levels in the CDX tumor-bearing mice were measured after 18 days of G C-K treatment, n=3. (**F**) The BUN levels in the CDX tumor-bearing mice were measured after 18 days of G C-K treatment, n=3. (ns represents P > 0.05 by one-way ANOVA)

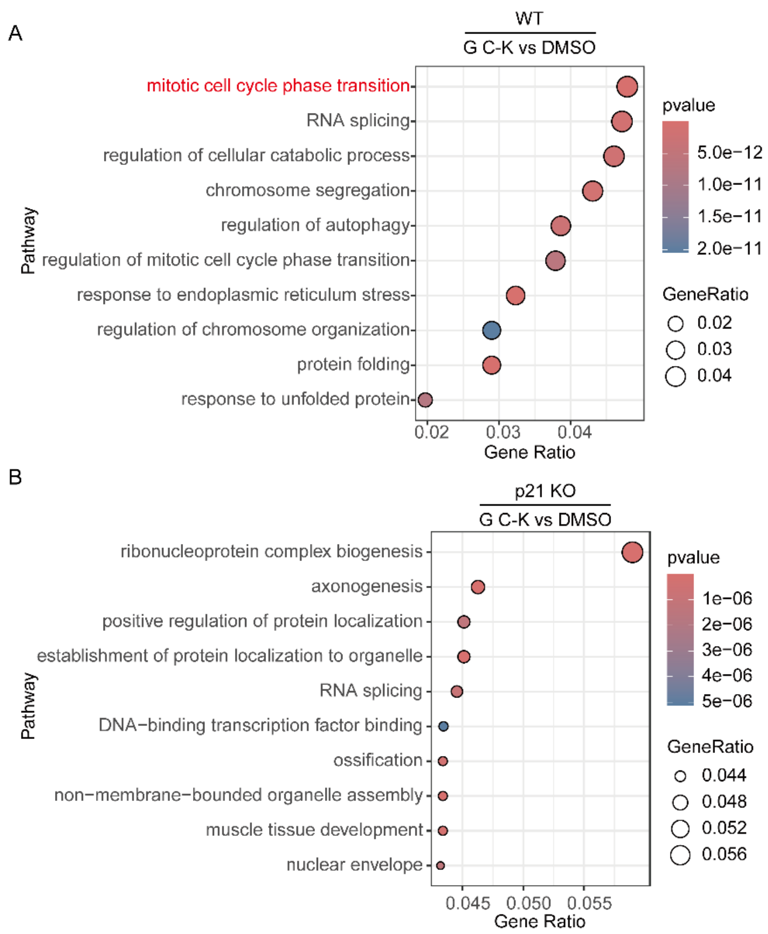

Supplementary Figure 9. G C-K-mediated mitotic cell cycle phase transition requires p21. (**A**) The top 10 enriched GO enrichment analysis in WT HGC27 cells with or without G C-K treatment. (**B**) The top 10 enriched GO enrichment analysis in p21 KO HGC27 cells with or without G C-K treatment. Vertical axis represents GO annotation, horizontal axis represents the gene ratio.
