# Supplementary material for: Poly(ADP-ribose) glycohydrolase enforces p21 degradation via dePARylation to promote gastric cancer progression
Source: J Clin Invest. 2026 Jan 15;136(5):e195538. doi: 10.1172/JCI195538 (PMC13067944; doi:10.1172/JCI195538)

Full unedited blot for Figure 1

Figure 1C

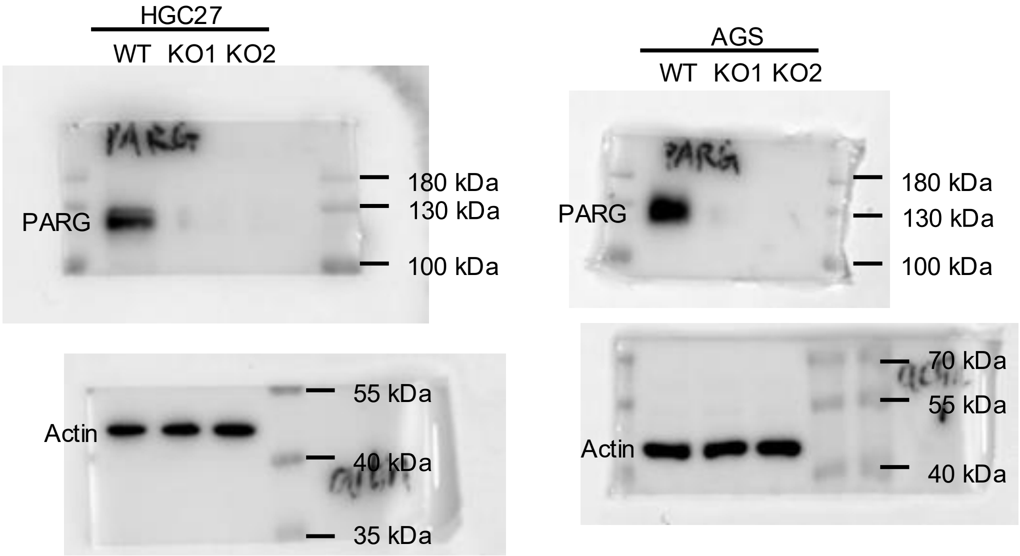

Full unedited blot for Figure 2

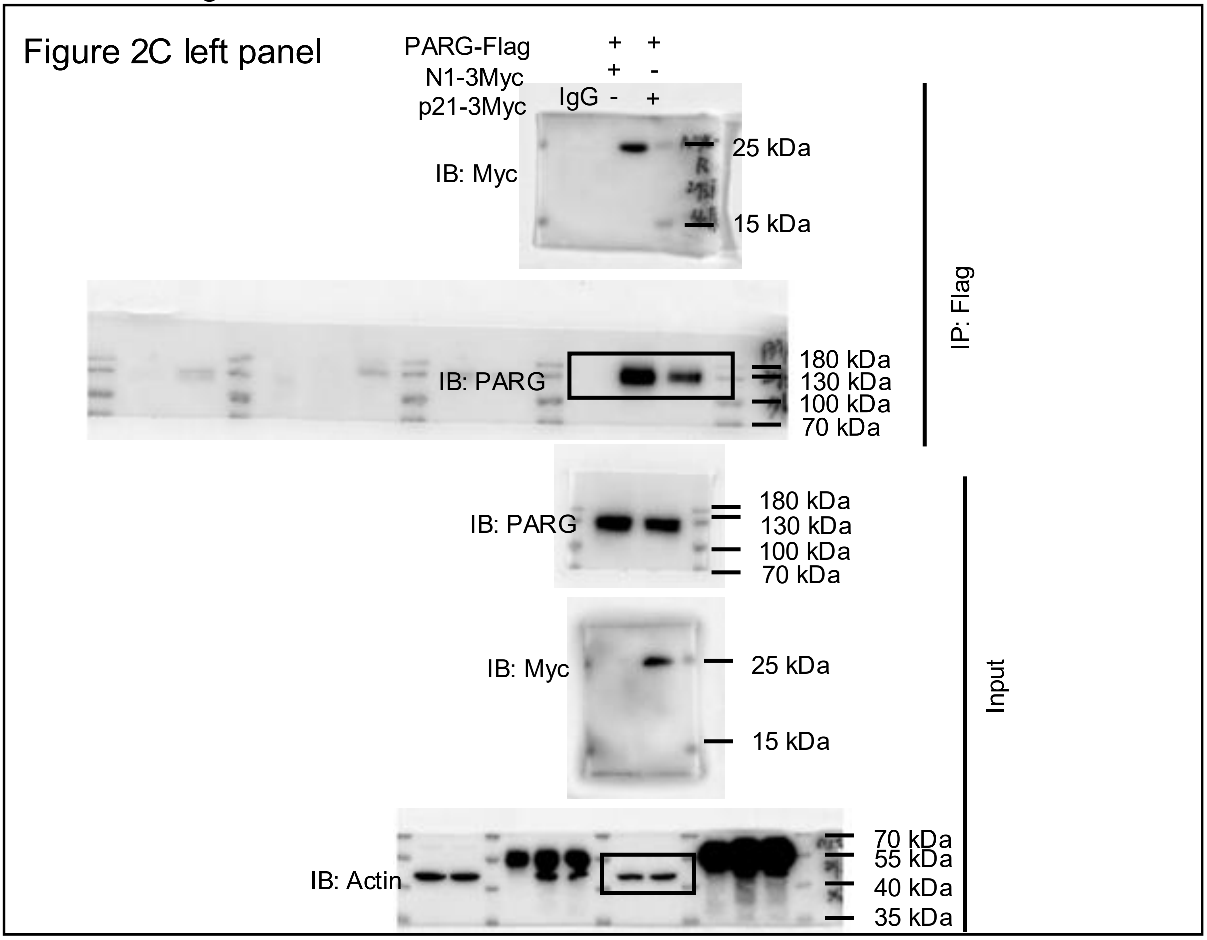

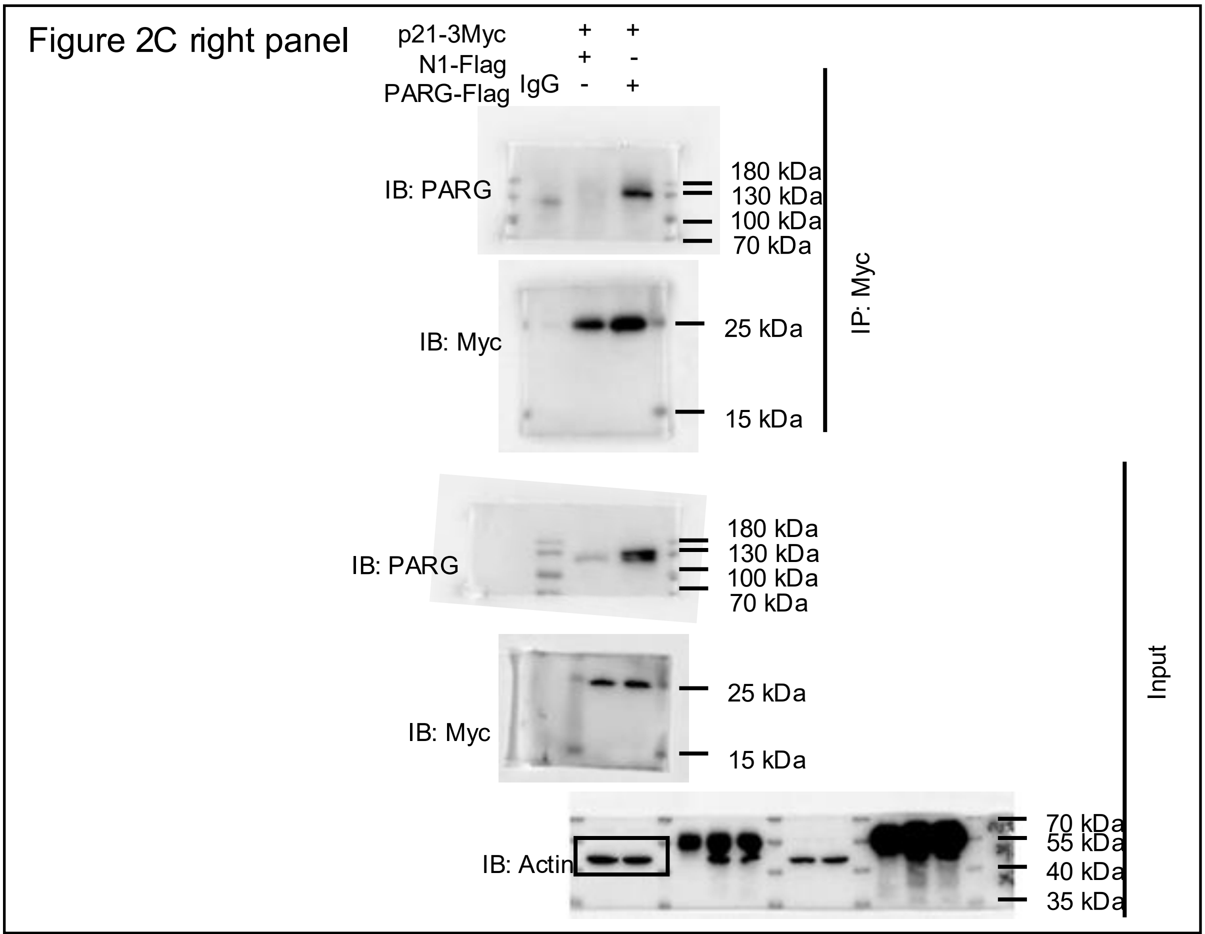

Full unedited blot for Figure 2

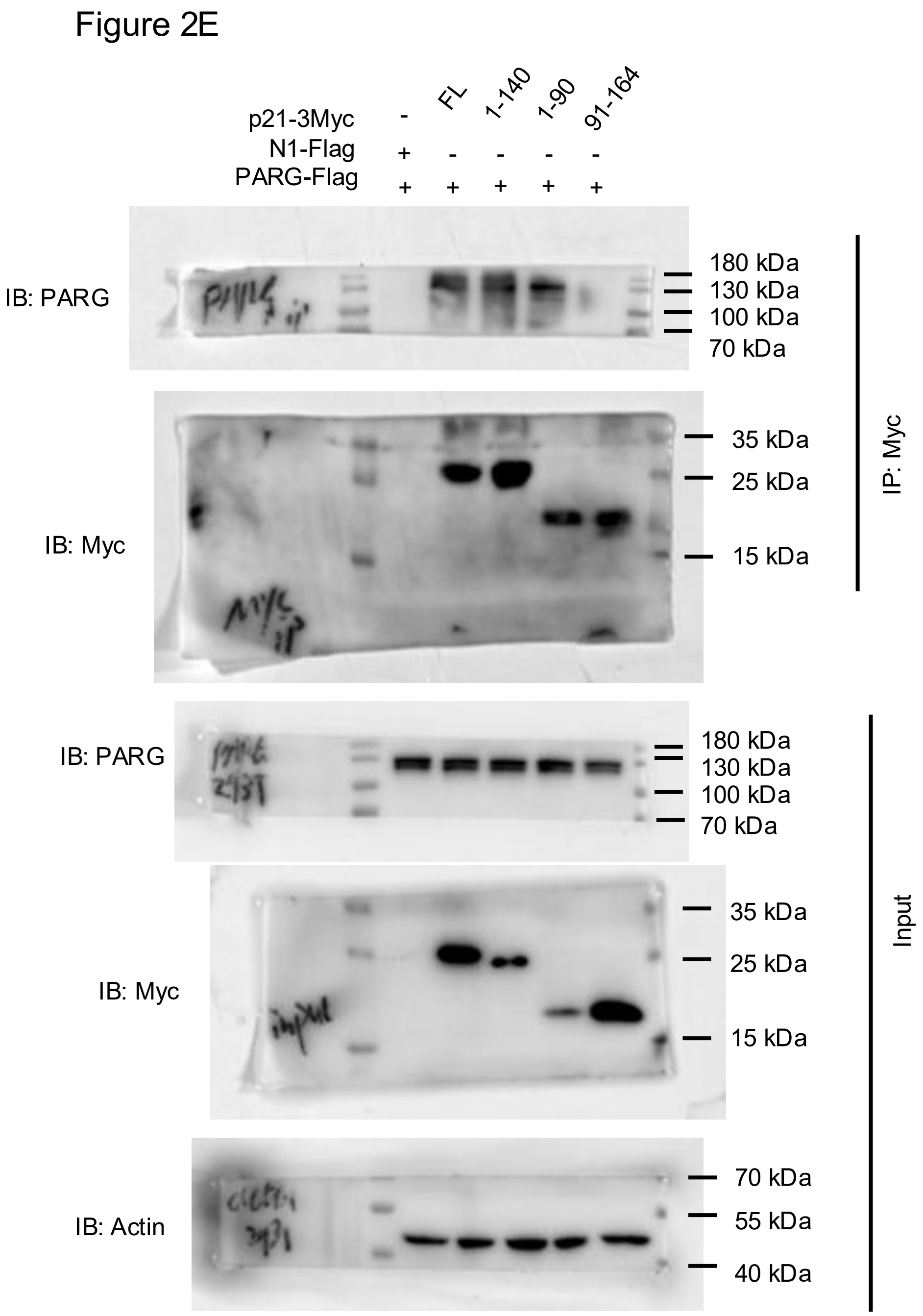

Full unedited blot for Figure 2

Figure 2G

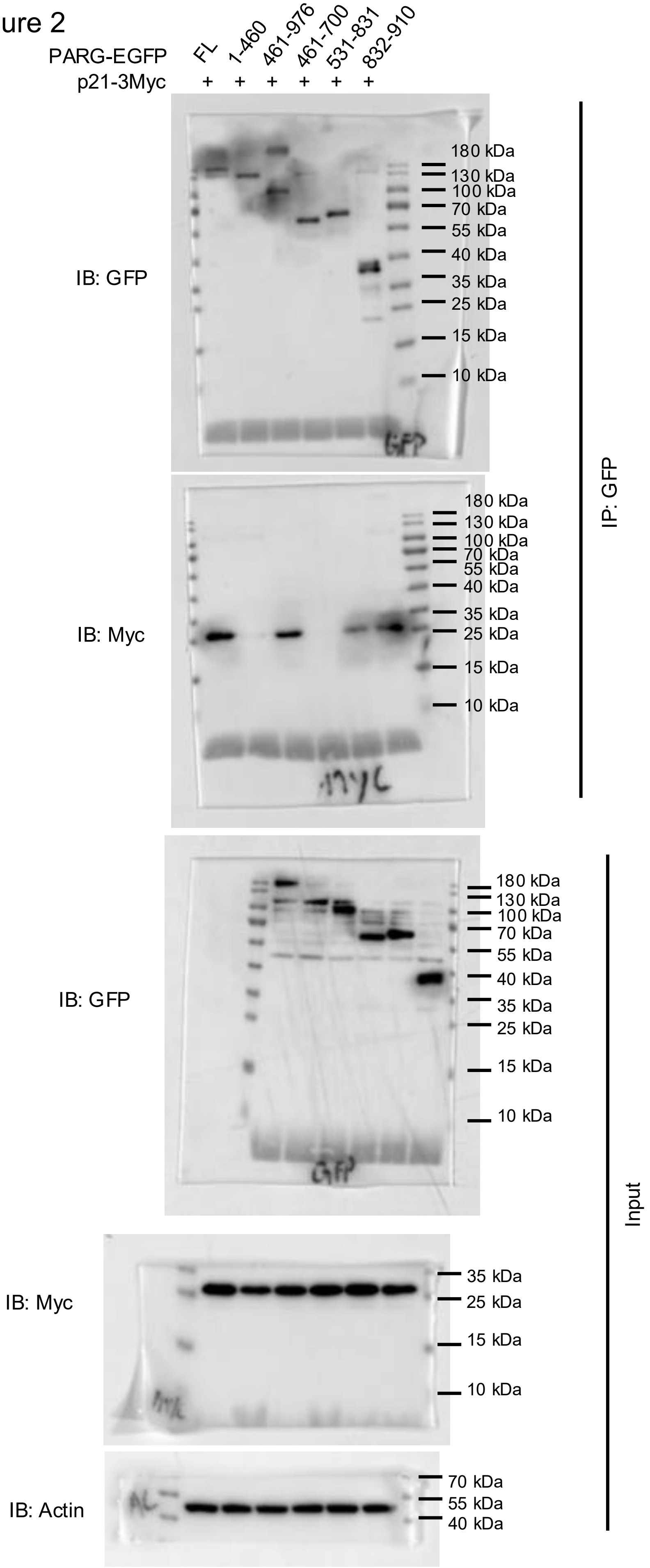

Full unedited blot for Figure 3

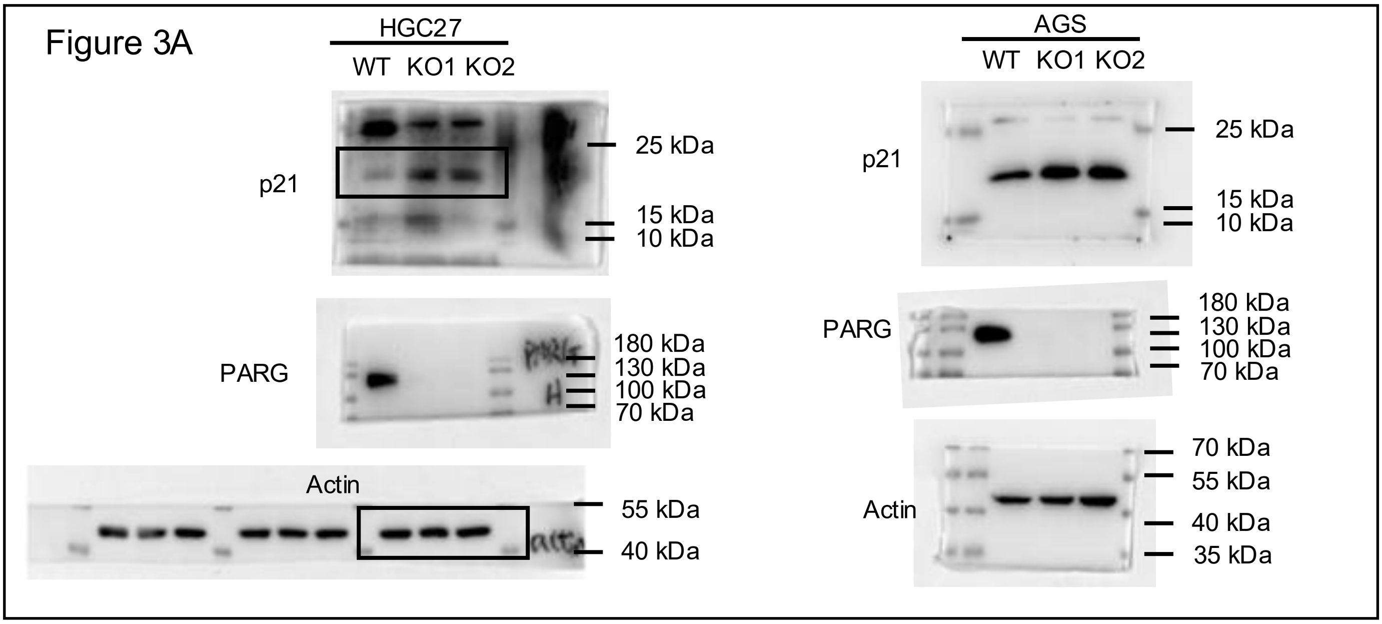

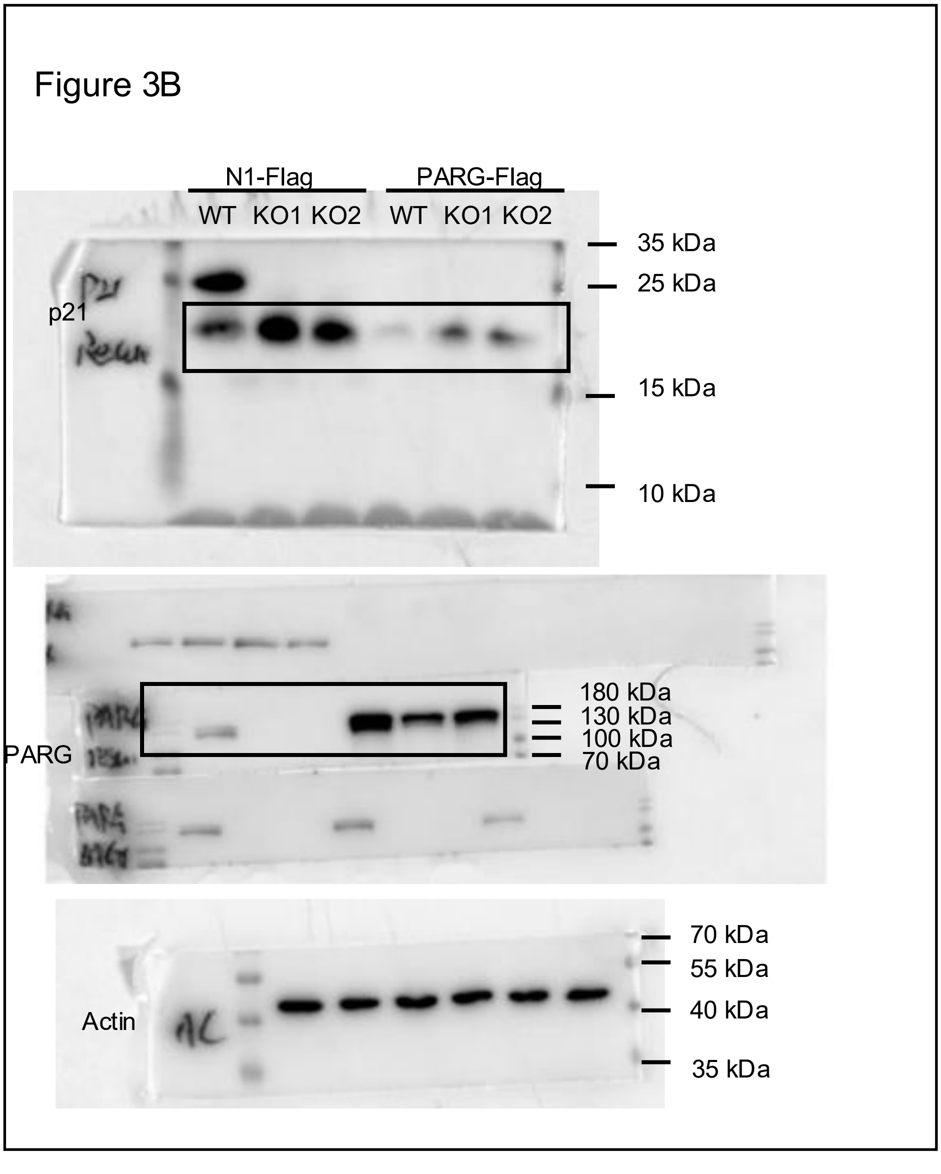

Full unedited blot for Figure 3

Figure 3C

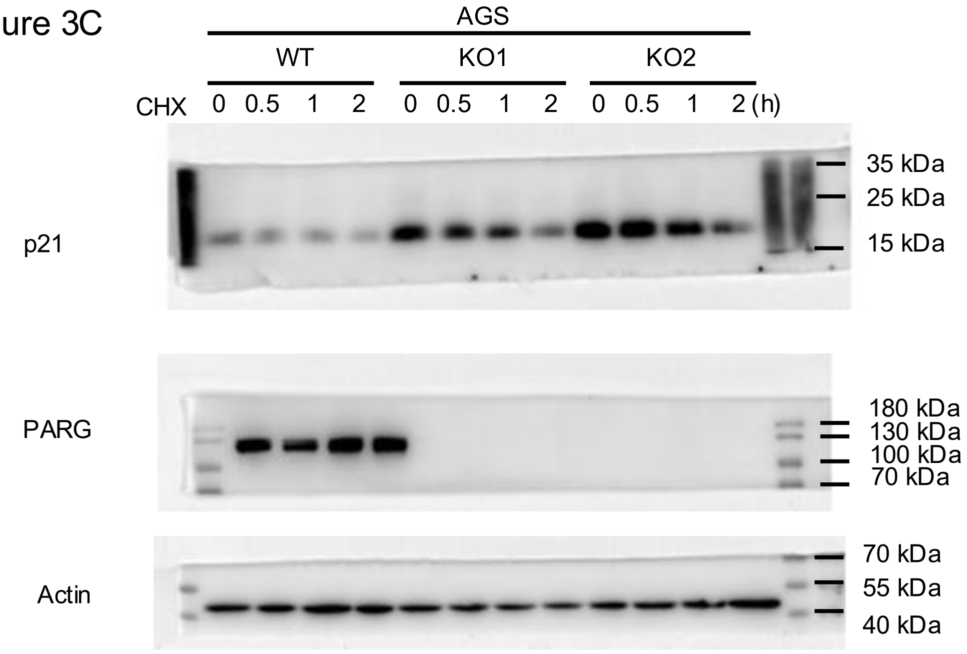

Figure 3D

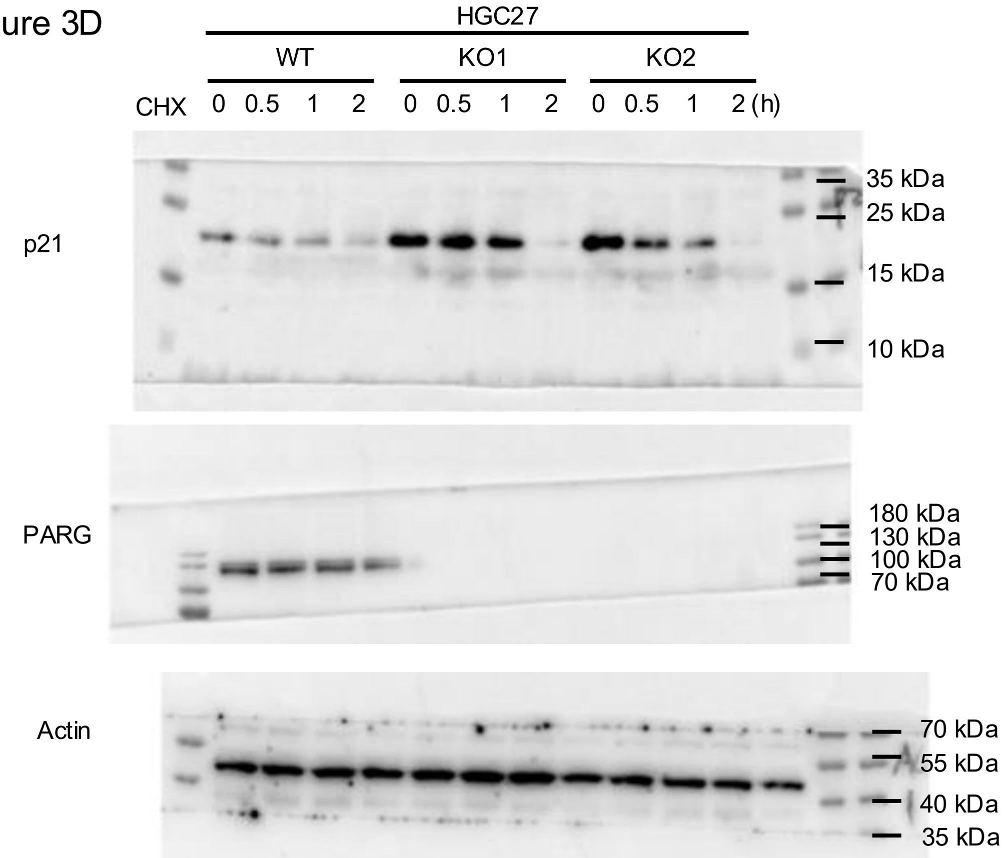

Full unedited blot for Figure 3

Figure 3E

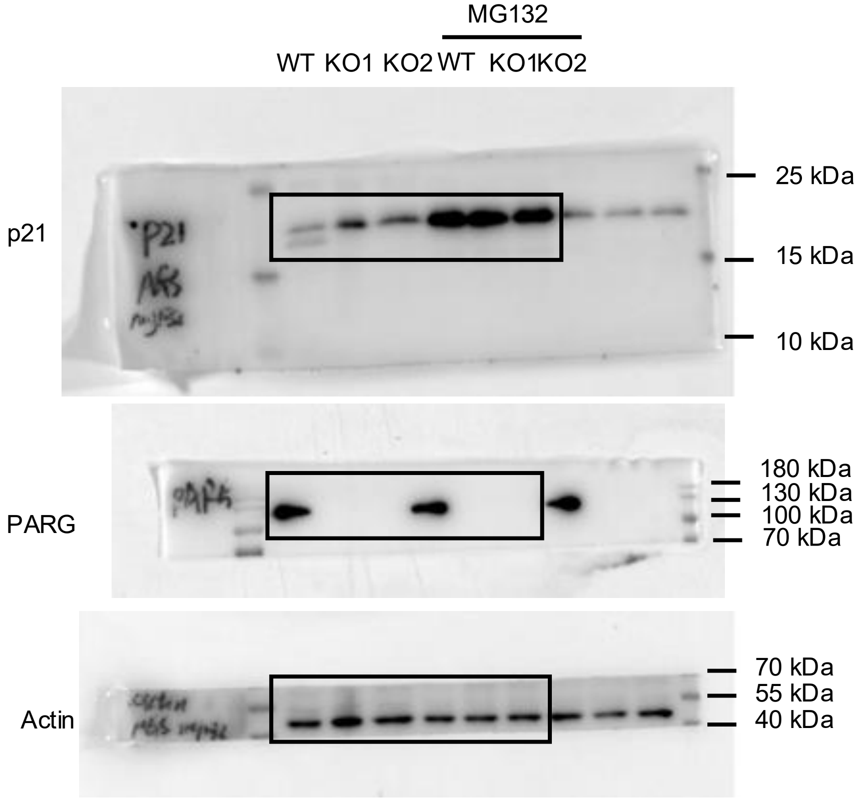

Figure 3F

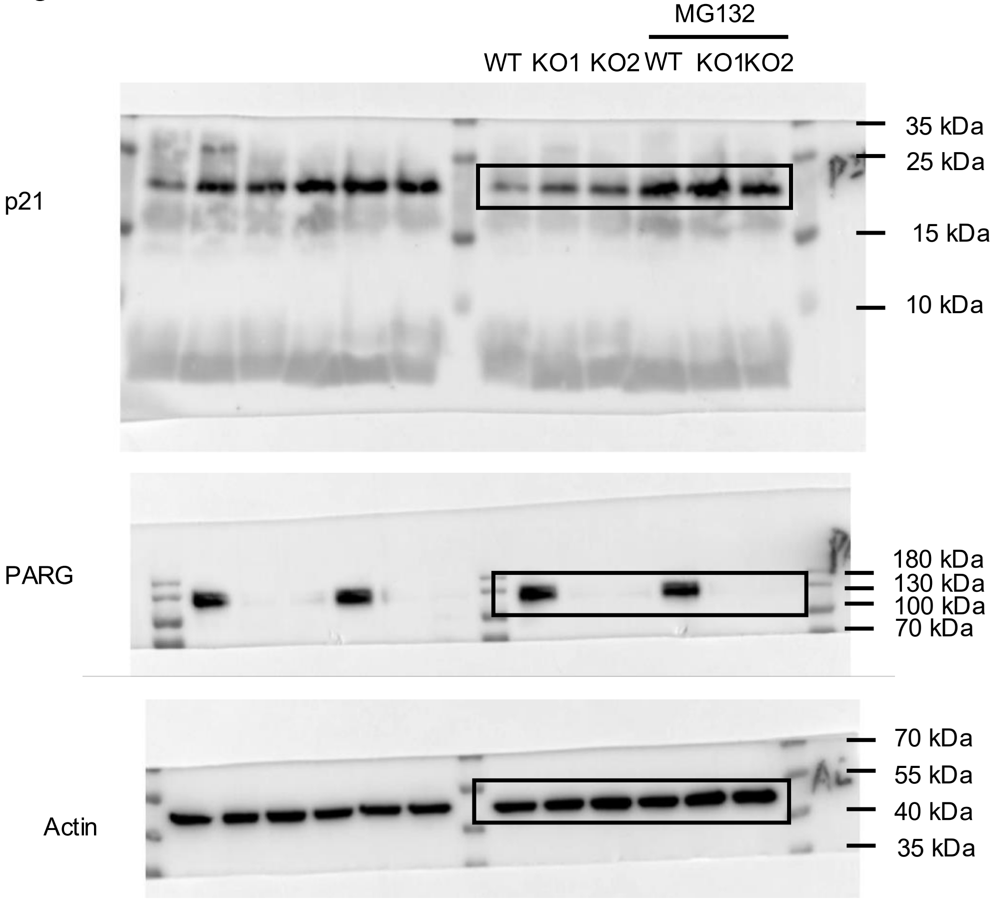

Full unedited blot for Figure 3

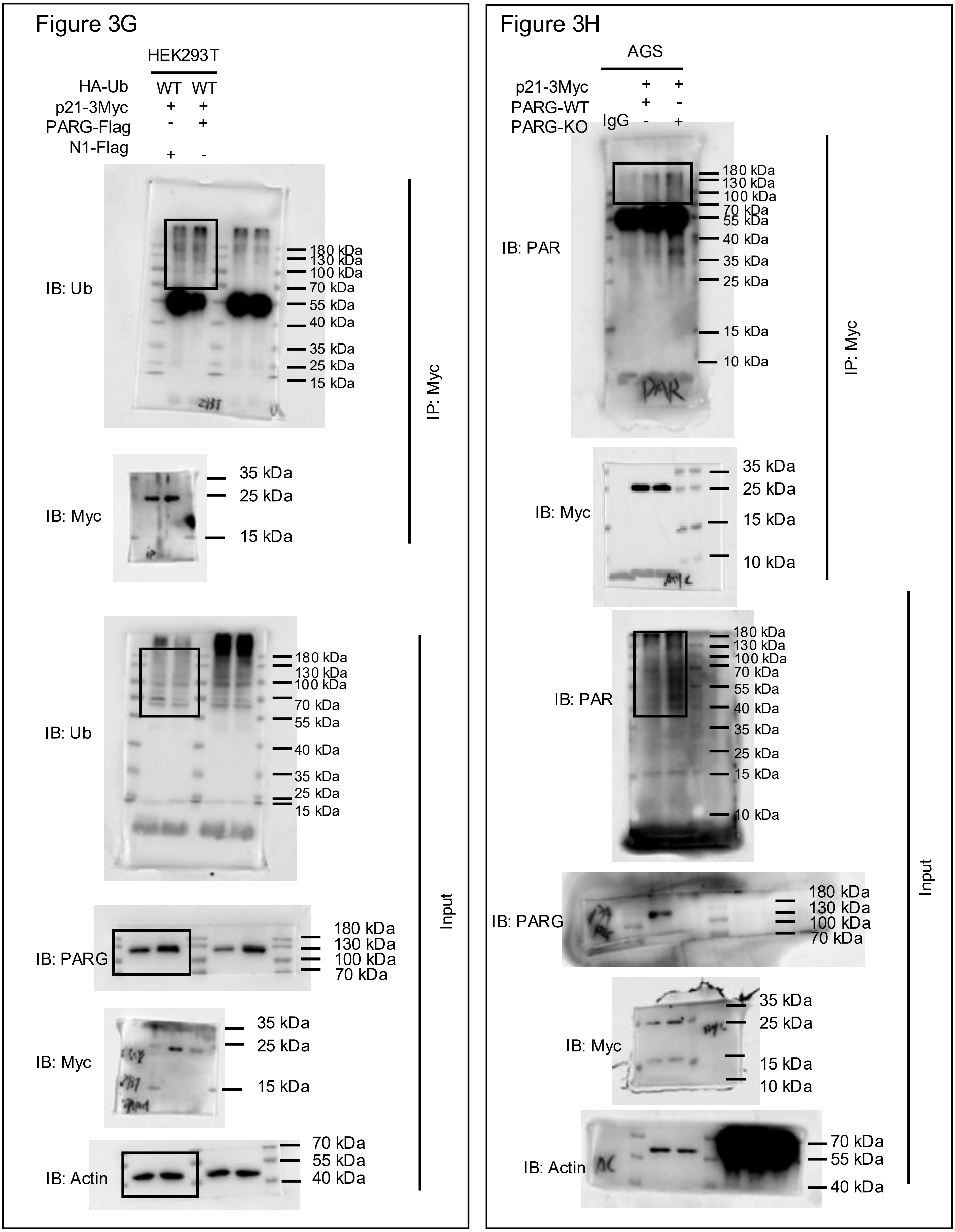

Full unedited blot for Figure 3

Figure 3I

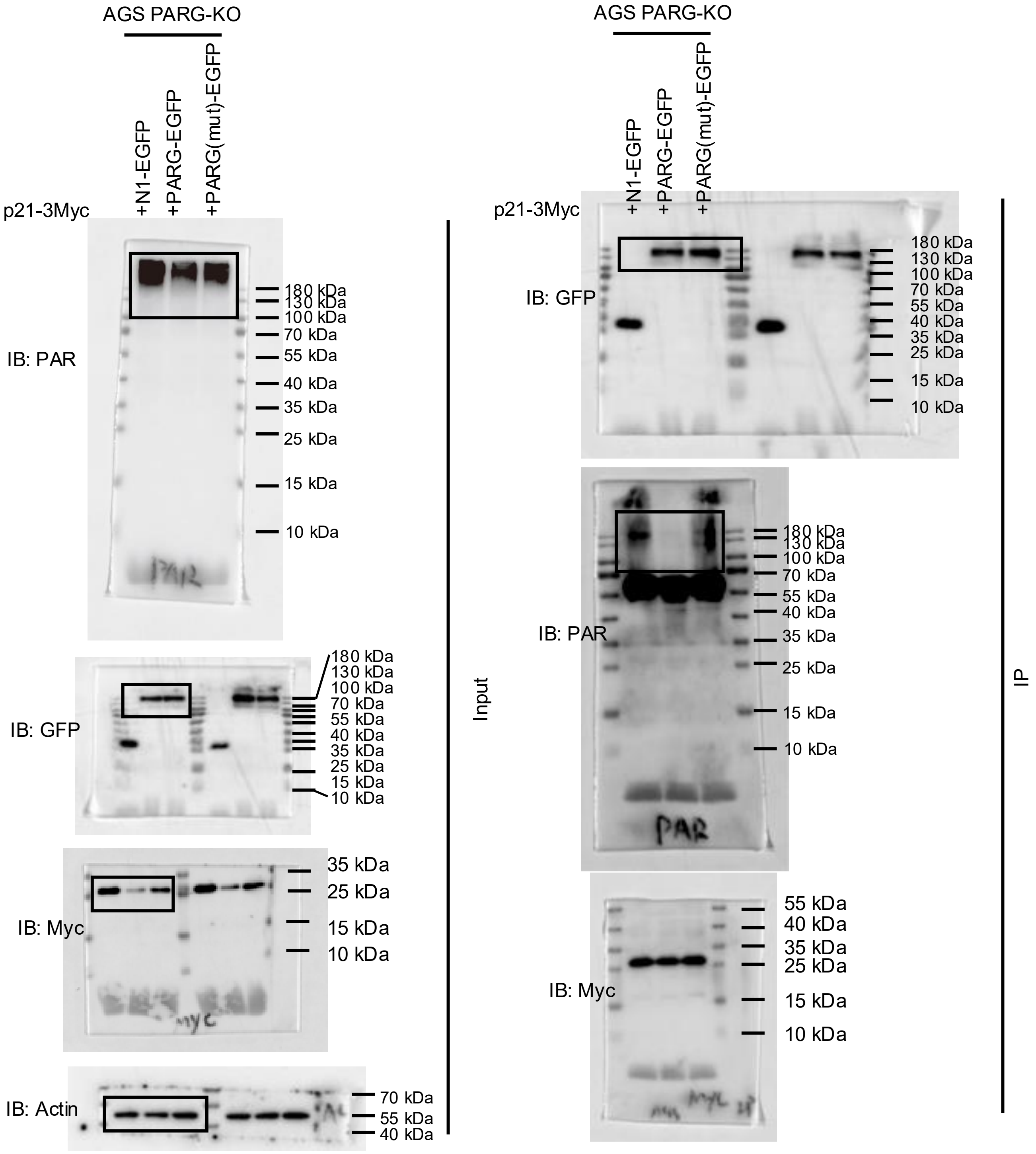

Full unedited blot for Figure 3

Figure 3J

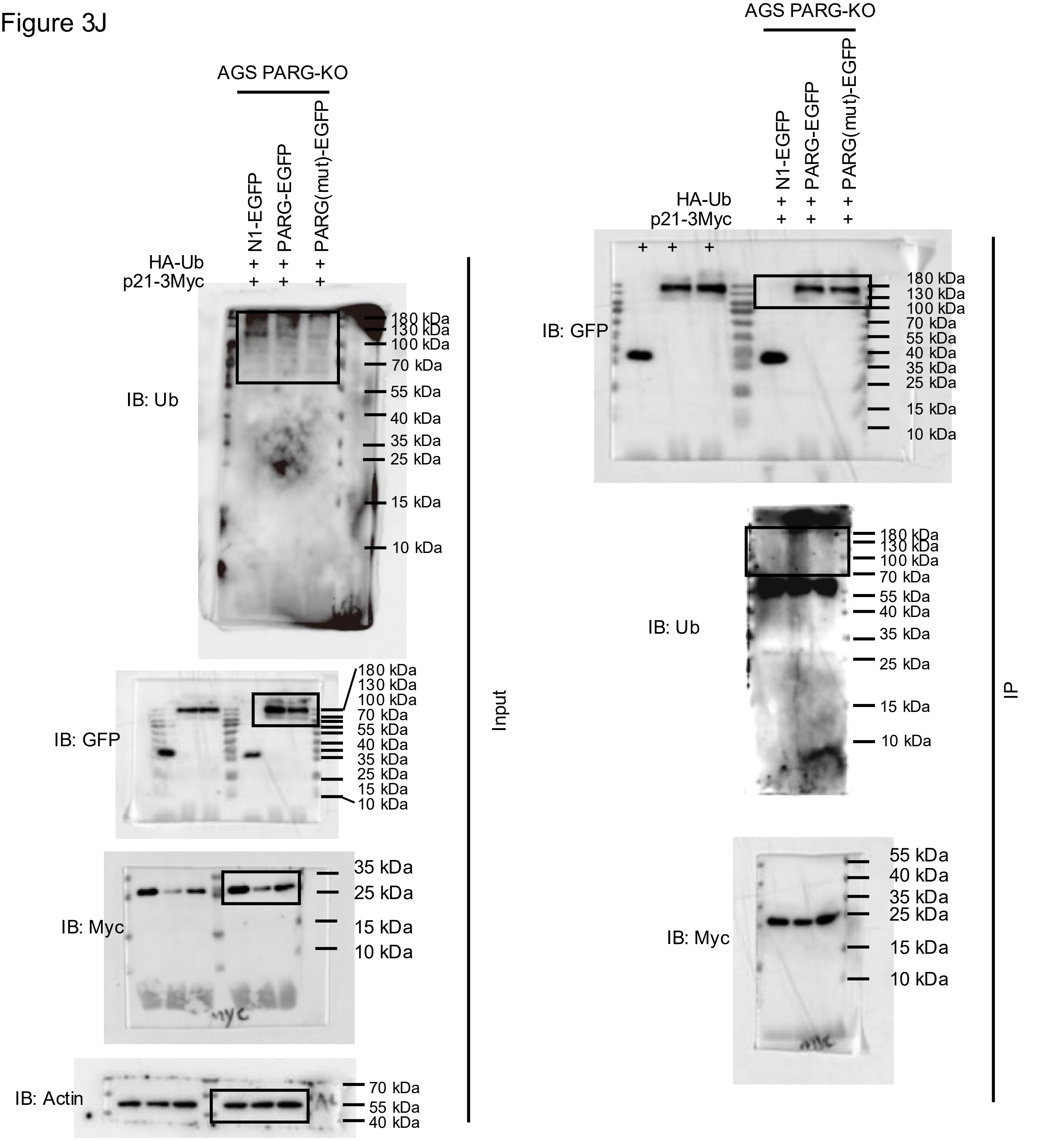

Full unedited blot for Figure 7

Figure 7A

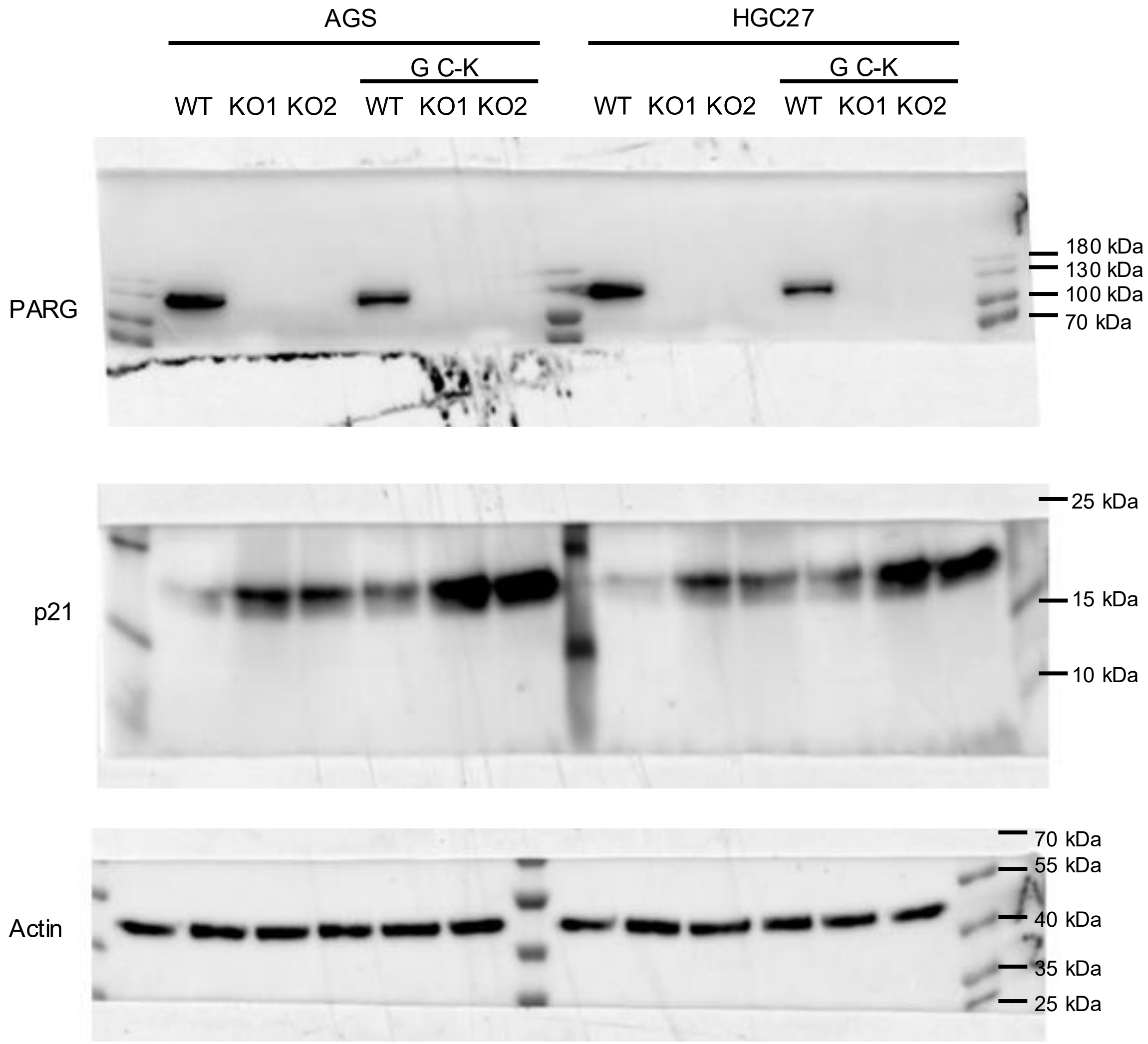

Full unedited blot for Supplementary Figure 1

Supplementary Figure 1A

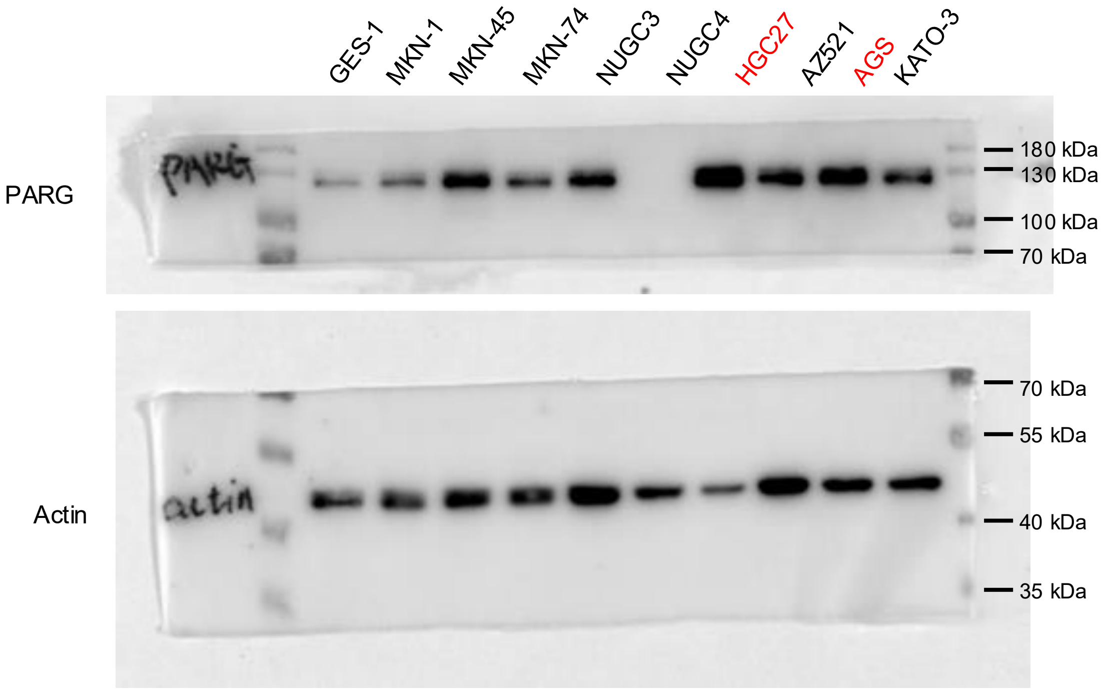

Full unedited blot for Supplementary Figure 4

Supplementary Figure 4A left panel

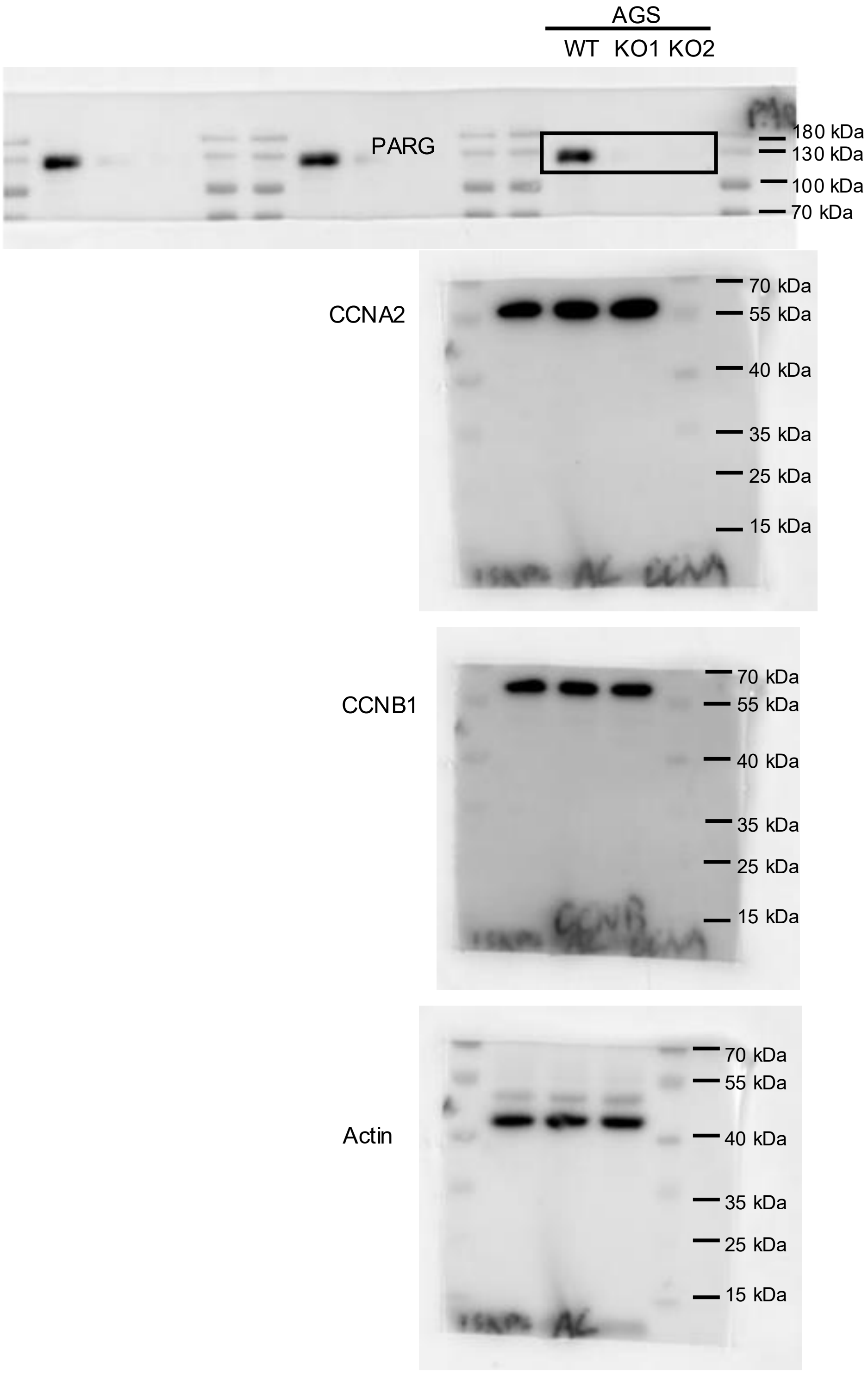

Full unedited blot for Supplementary Figure 4

Supplementary Figure 4A right panel

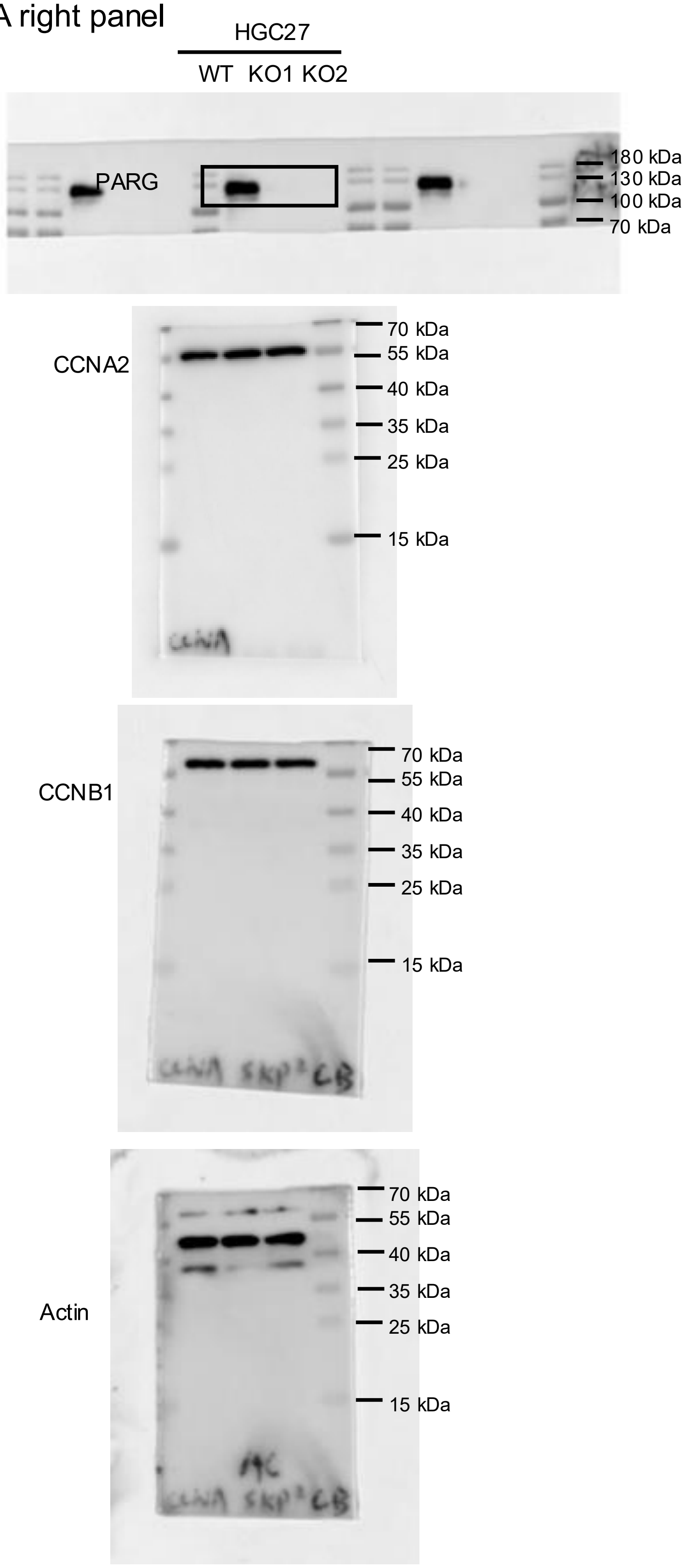

Full unedited blot for Supplementary Figure 4

Supplementary Figure 4B

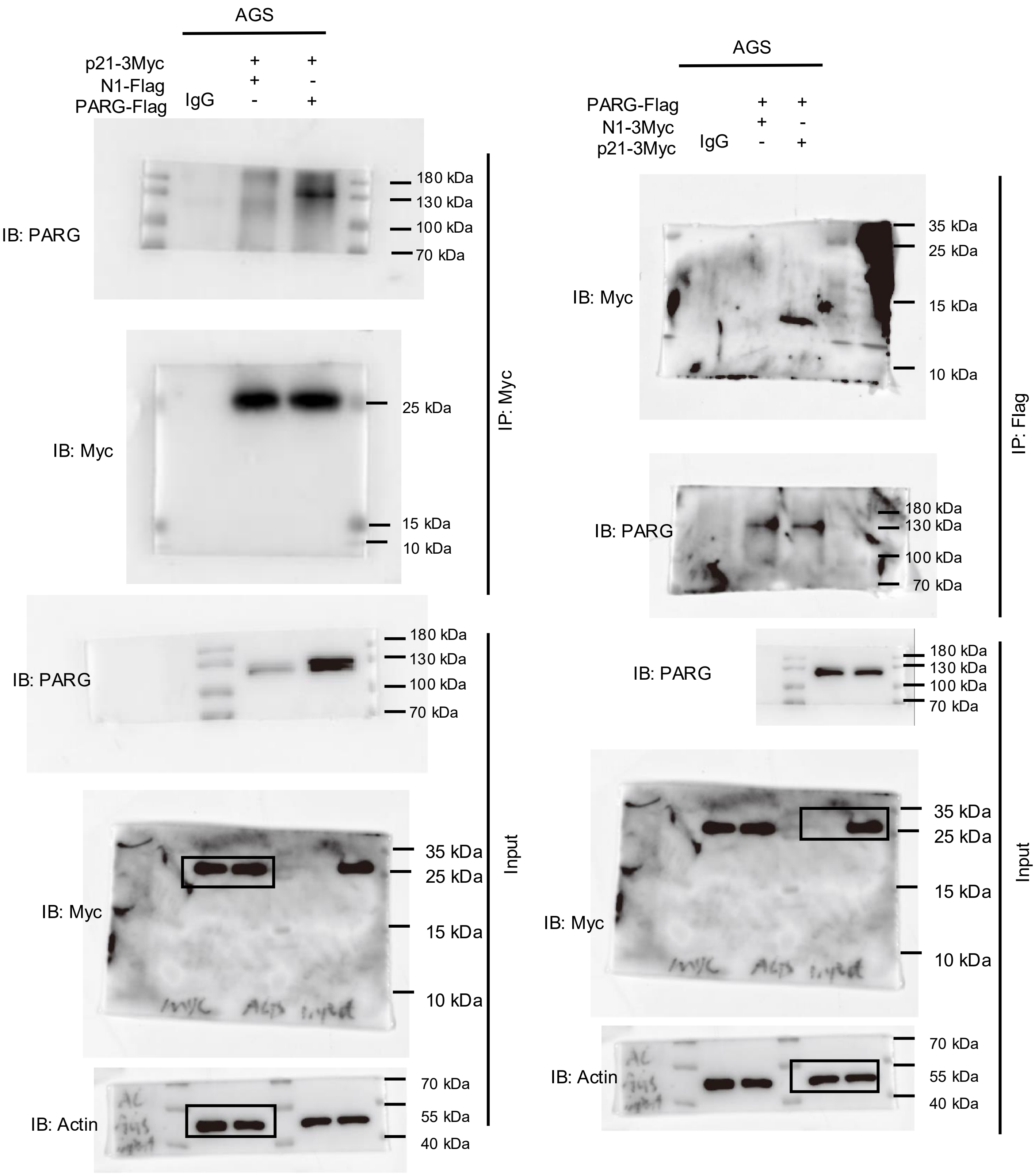

Full unedited blot for Supplementary Figure 4

Supplementary Figure 4C

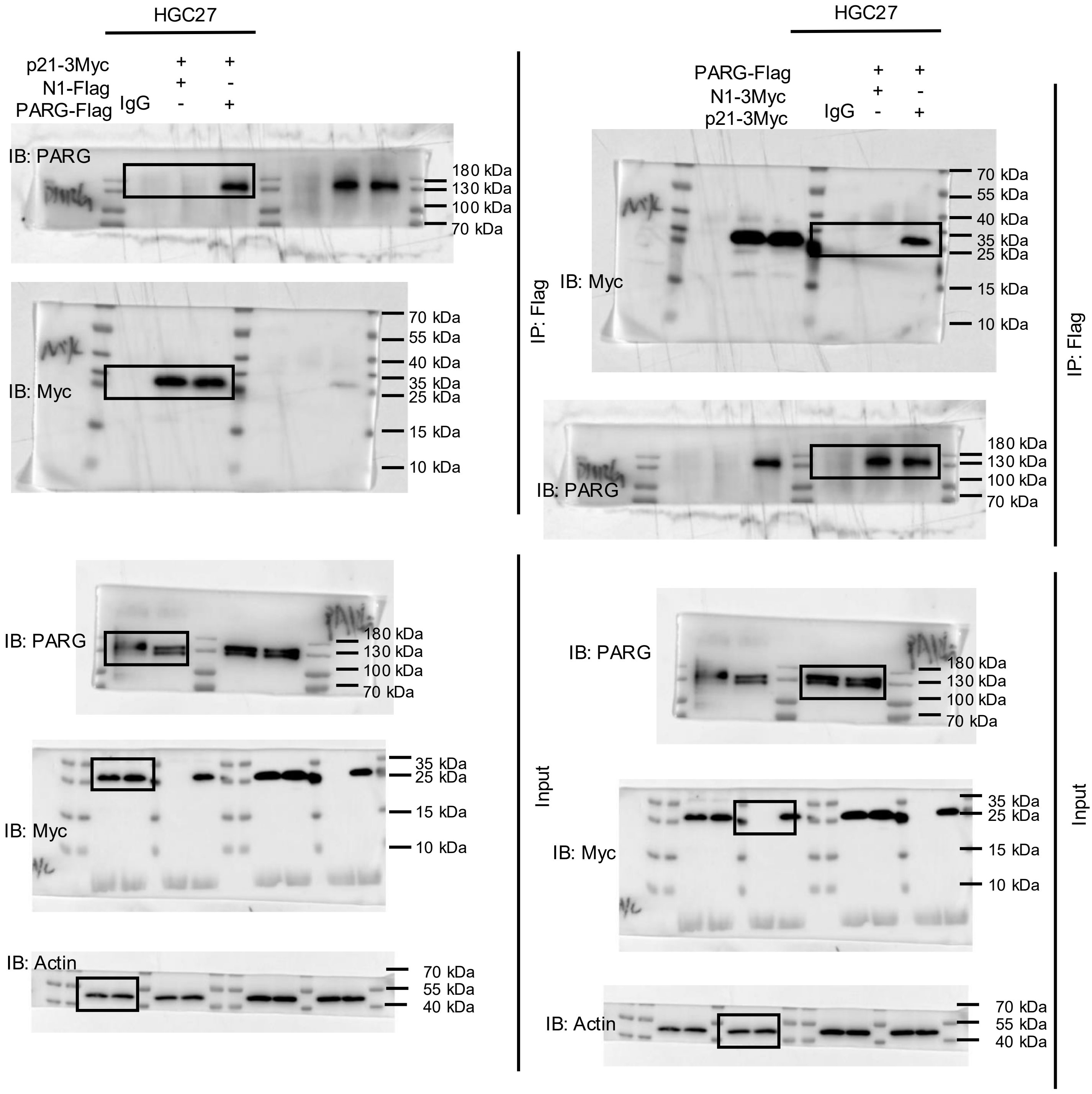

Full unedited blot for Supplementary Figure 4

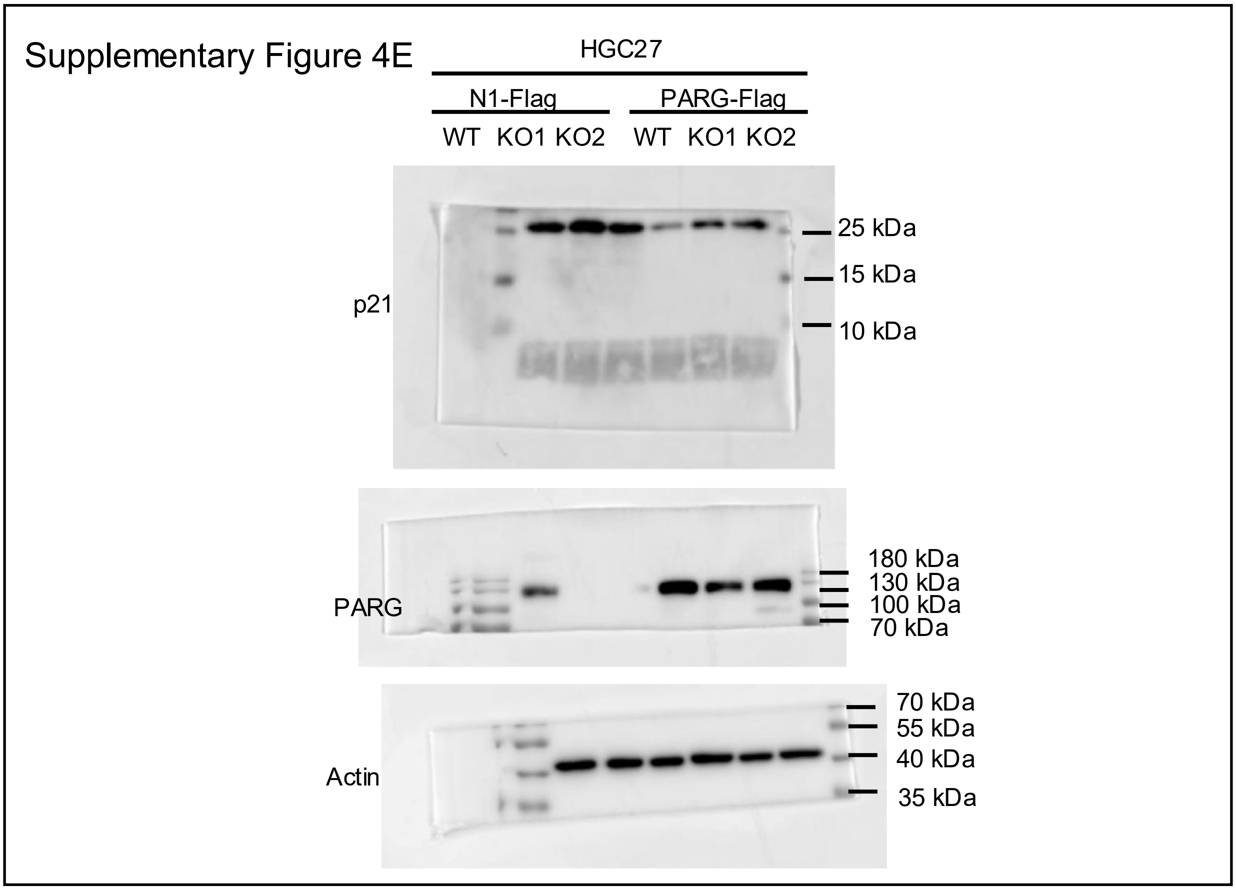

Full unedited blot for Supplementary Figure 4

Supplementary Figure 4G

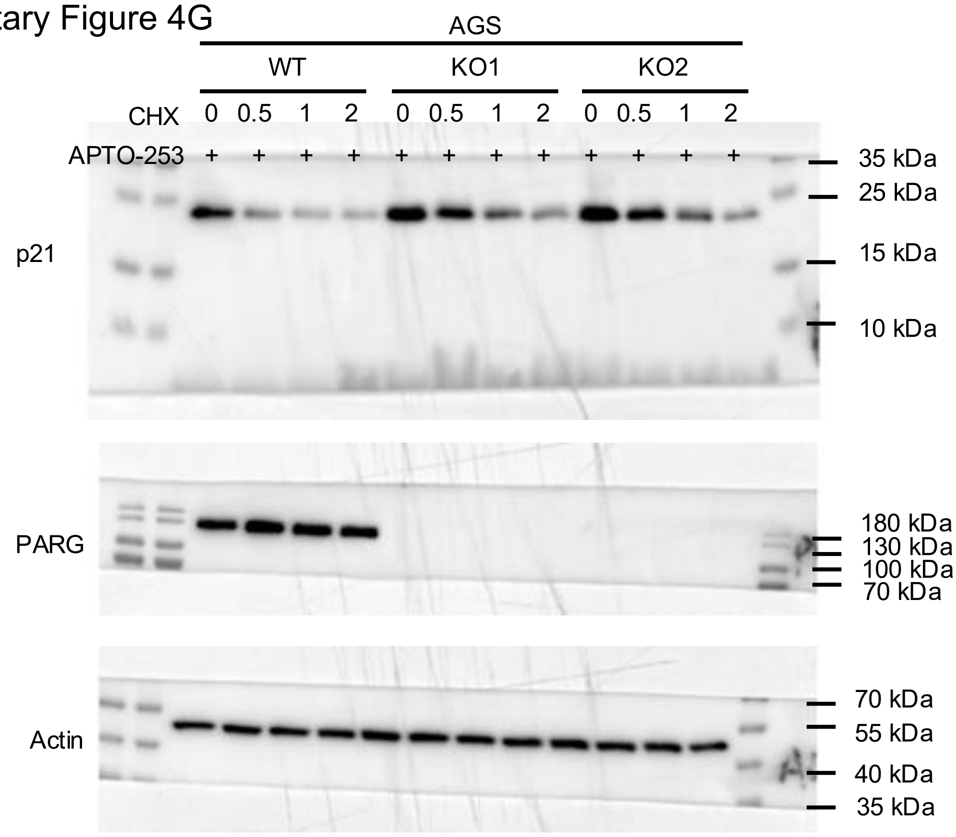

Supplementary Figure 4H

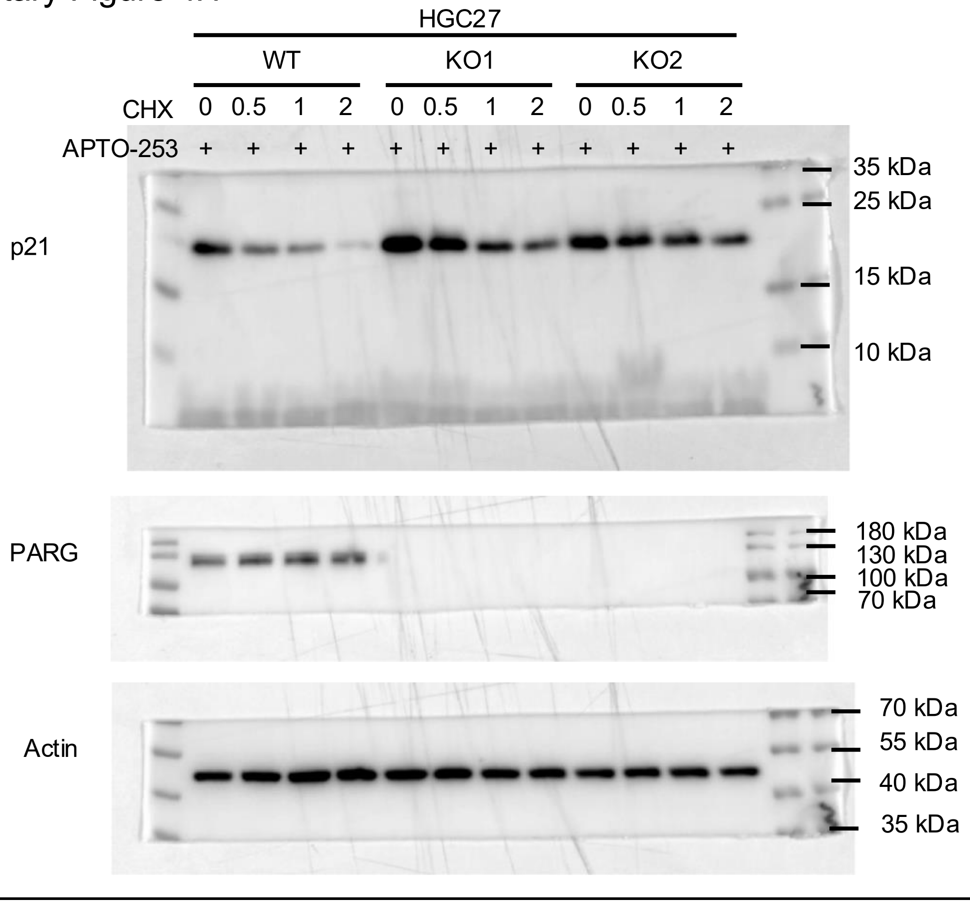

Full unedited blot for Supplementary Figure 5

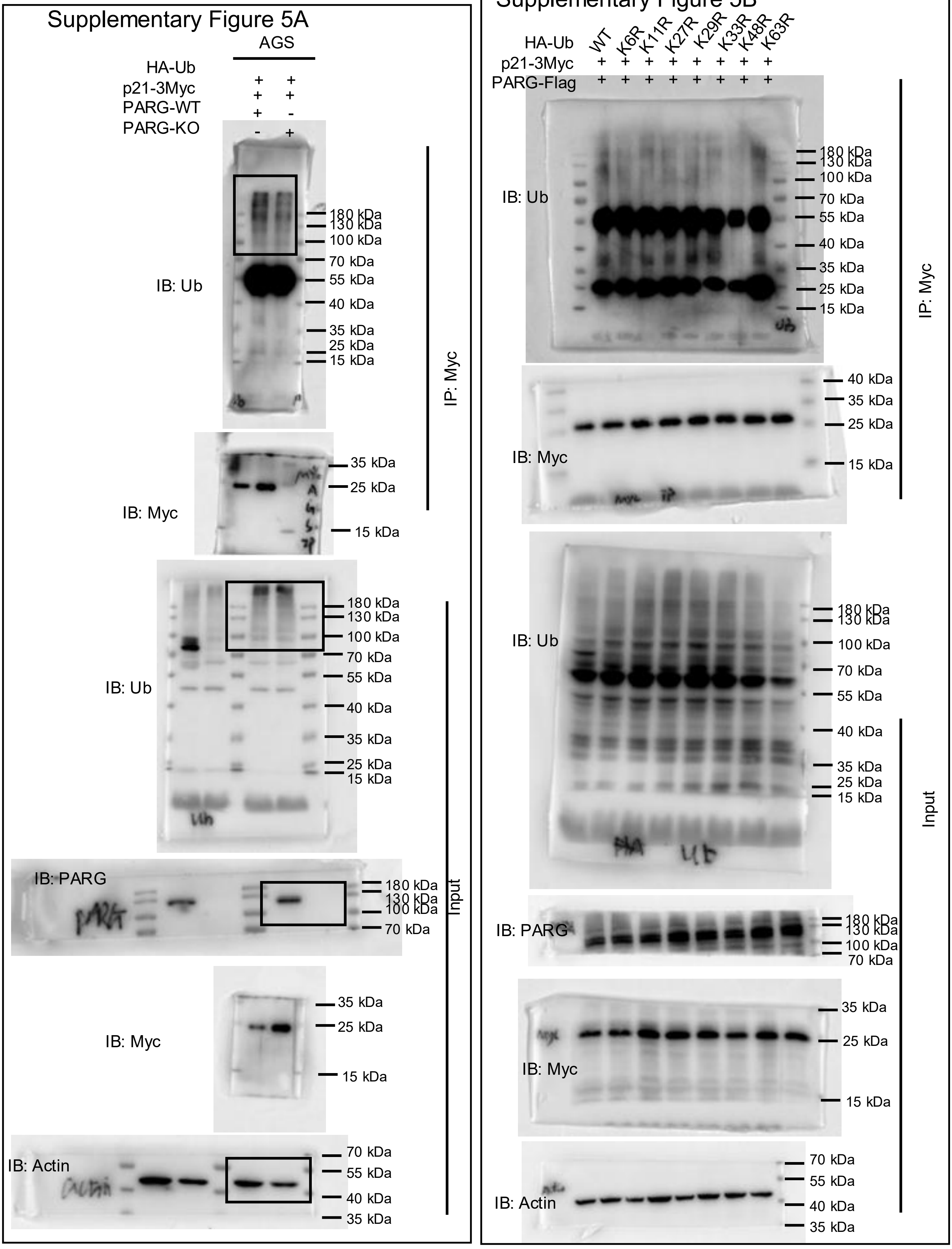

Full unedited blot for Supplementary Figure 5

Figure 5C

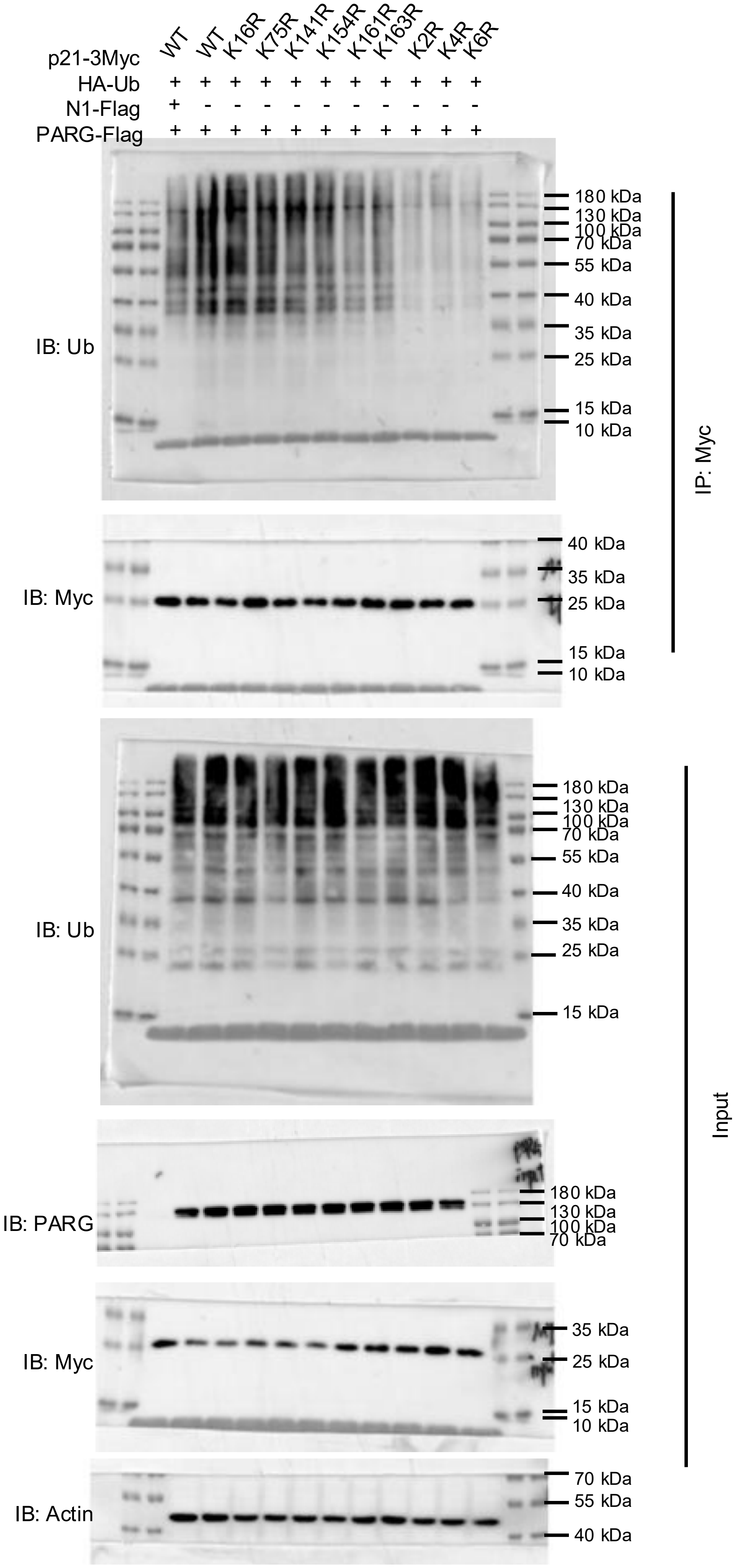

Full unedited blot for Supplementary Figure 5

Supplementary Figure 5D

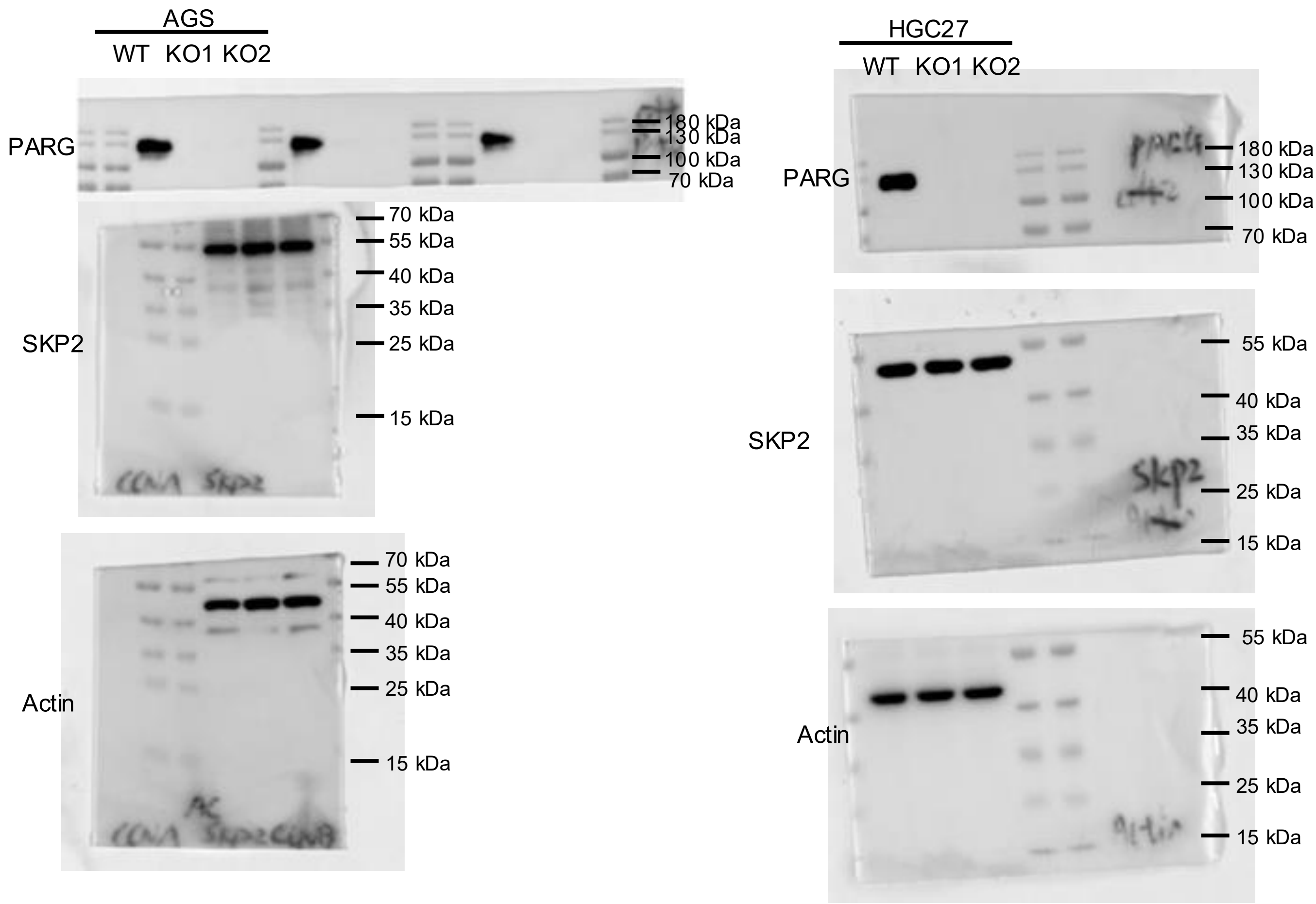

Full unedited blot for Supplementary Figure 5

Supplementary Figure 5E

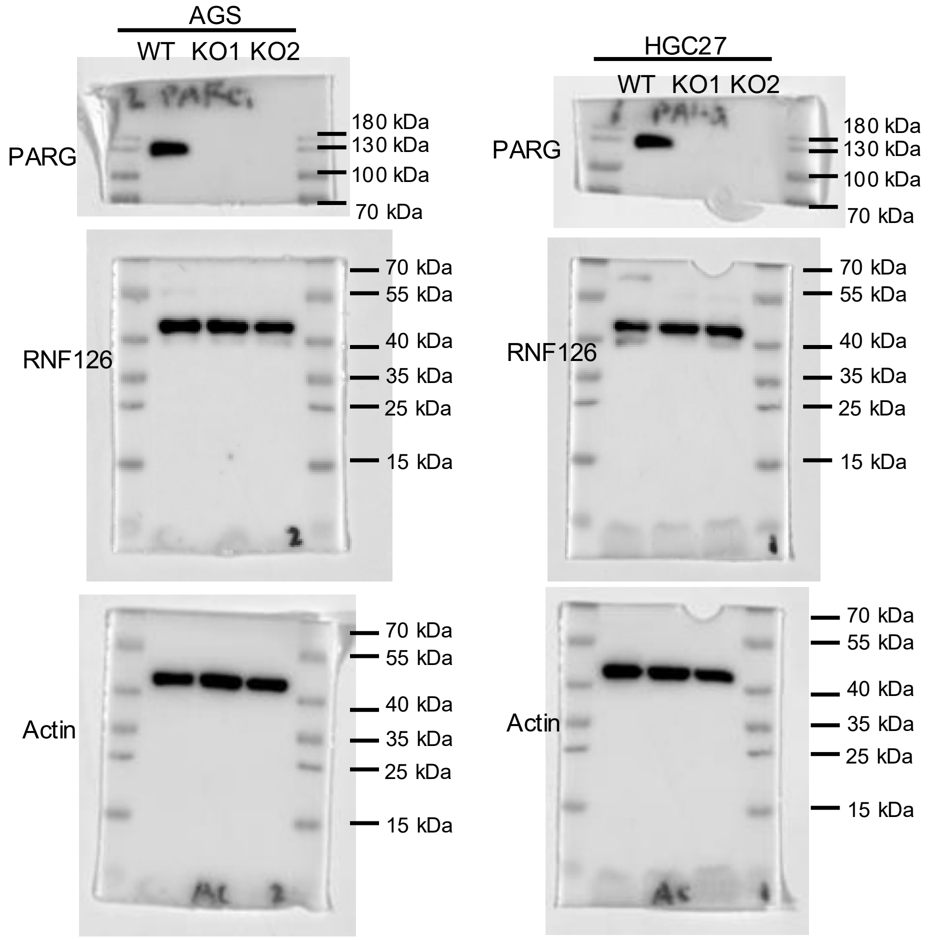

Full unedited blot for Supplementary Figure 5

Supplementary Figure 5F

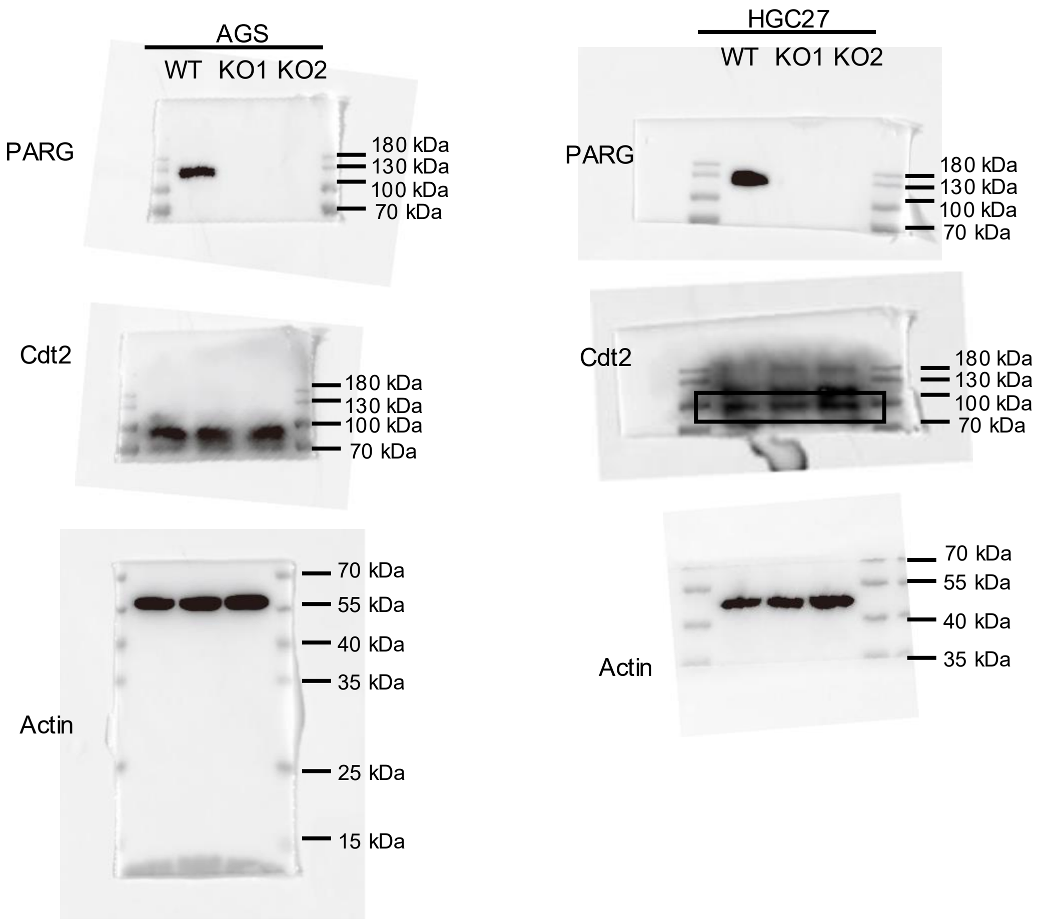

Full unedited blot for Supplementary Figure 5

Supplementary Figure 5G

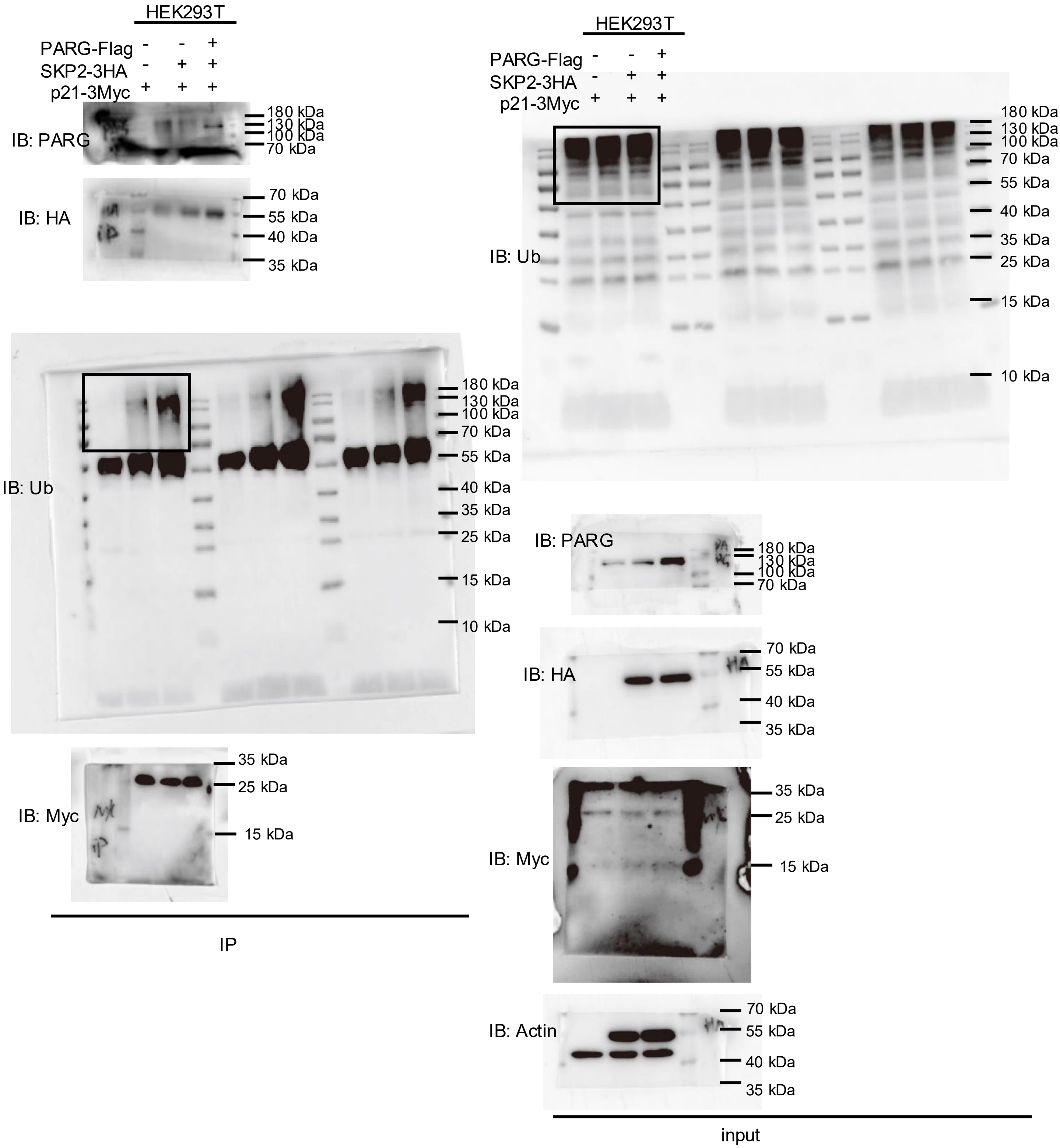

Full unedited blot for Supplementary Figure 5

Supplementary Figure 5H

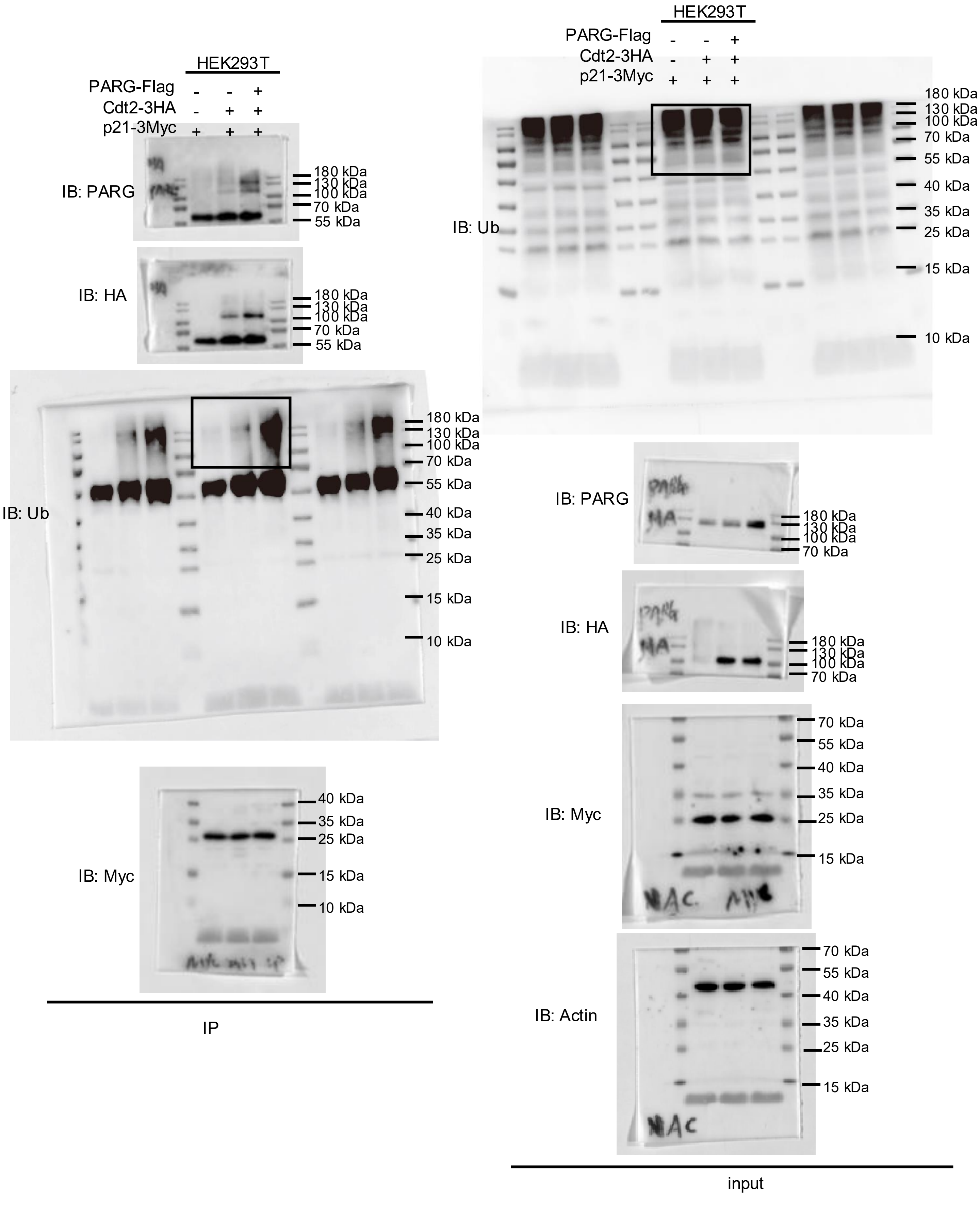

Full unedited blot for Supplementary Figure 5

Supplementary Figure 5I

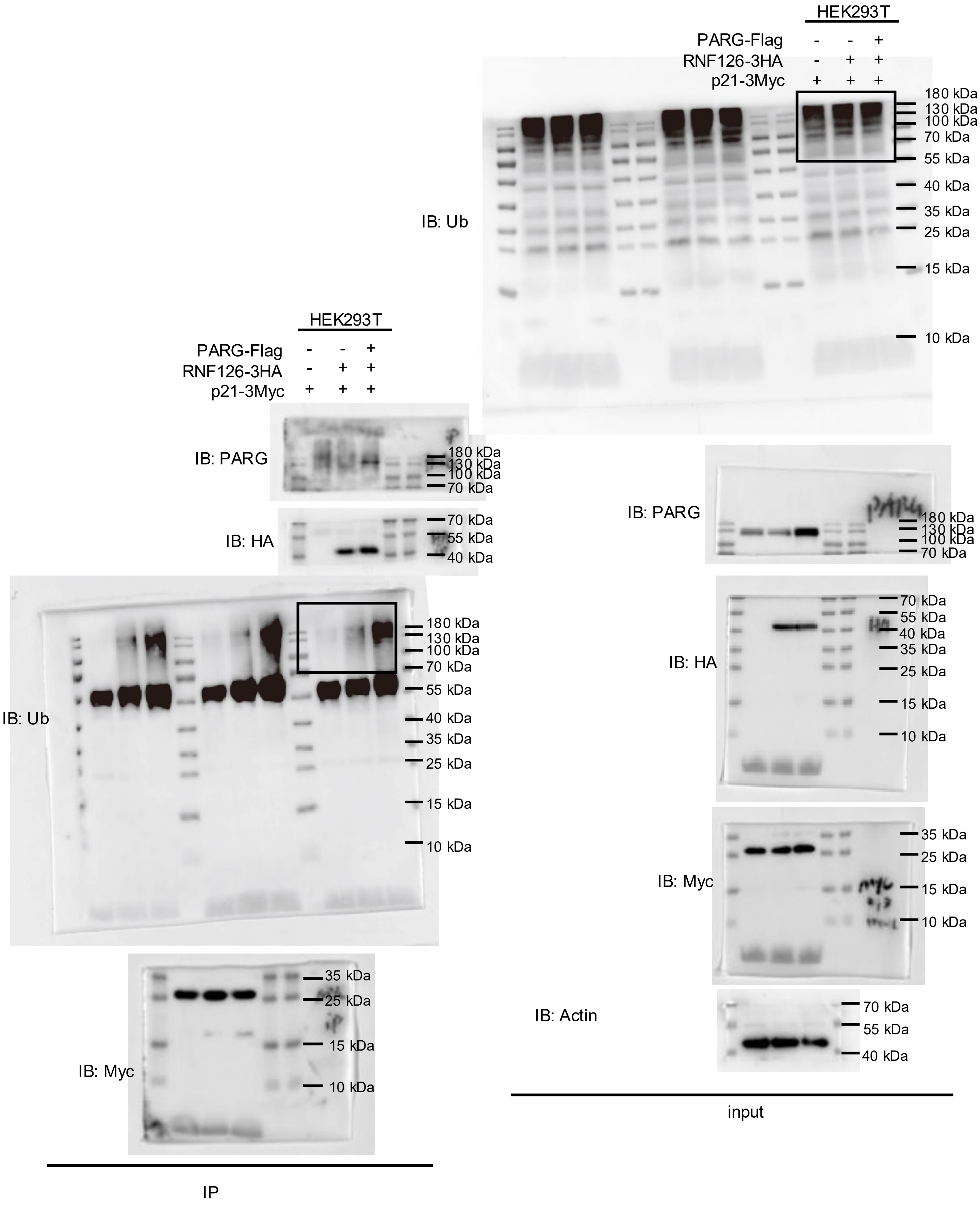

Full unedited blot for Supplementary Figure 6

Supplementary Figure 6A

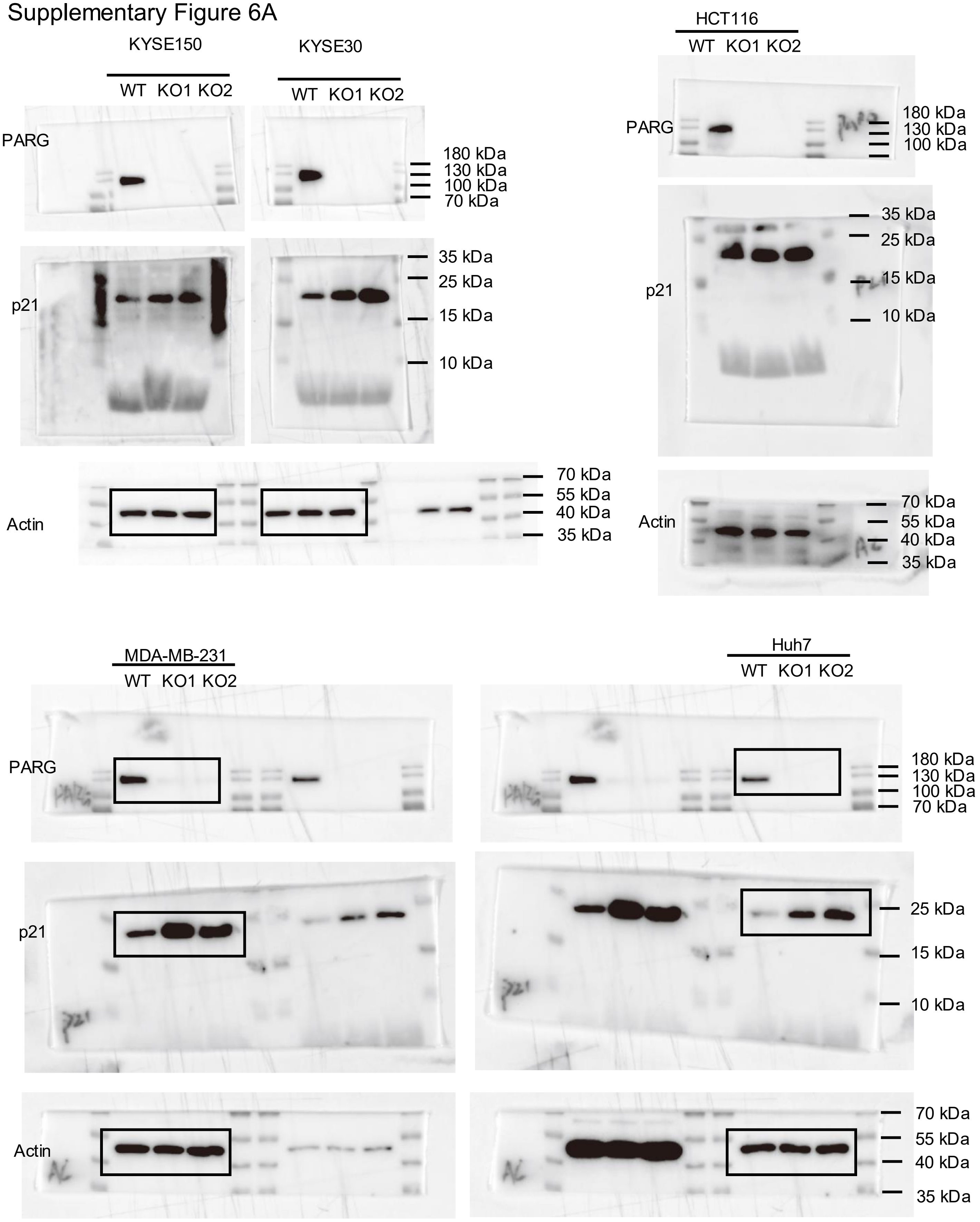

Full unedited blot for Supplementary Figure 7

Supplementary Figure 7A

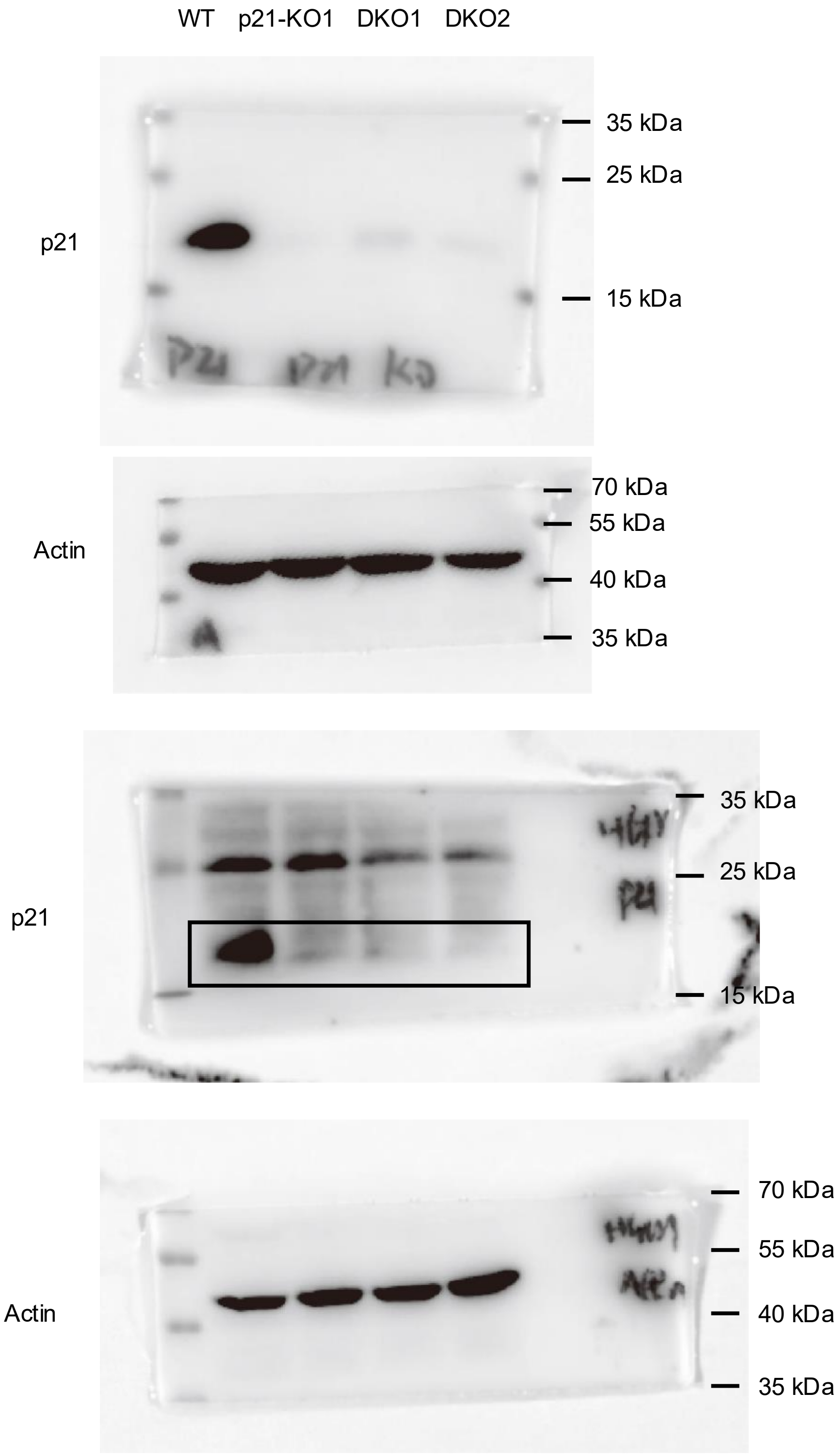

Supplement: Unedited blot and gel images [file jci-136-195538-s294.pdf]
